# Fear of childbirth among antenatal care attendees in a primary care setting in Kumasi, Ghana: Prevalence and parity-stratified determinants

**DOI:** 10.1371/journal.pone.0358457

**Published:** 2026-09-21

**Authors:** Juliana Ofosua Addo, Douglas Aninng Opoku, Julius Kwabena Karikari, Emmanuel Konadu, Mercy Addae, Francis Kwabena Kyei, Mawuse Kanfra, Obed Kwabena Offe Amponsah, Eric Adjei Boadu

**Affiliations:** 1 Department of Population, Family and Reproductive Health, School of Public Health, Kwame Nkrumah University of Science and Technology, Kumasi, Ghana; 2 HopeXchange Medical Centre, Kumasi, Ghana; 3 University Hospital, Kwame Nkrumah University of Science and Technology, Kumasi, Ghana; 4 Department of Epidemiology and Biostatistics, School of Public Health, Kwame Nkrumah University of Science and Technology, Kumasi, Ghana; 5 St. Dominic Catholic Hospital, Akwatia, Ghana; 6 Department of Pharmacy Practice, Faculty of Pharmacy and Pharmaceutical Sciences, Kwame Nkrumah University of Science and Technology, Kumasi, Ghana; Dilla University College of Health Sciences, ETHIOPIA

## Abstract

**Background:**

Previous studies on Fear of Childbirth (FoC) across sub-Saharan Africa have typically reported prevalence separately by parity or treated it as a covariate in multivariable analyses, with little attention given to identifying parity-specific determinants. However, first-time mothers and those with previous birth experiences may require different forms of antenatal support. To address this gap, we assessed the prevalence of FoC and identified parity-specific determinants among antenatal care attendees in Ghana to inform targeted interventions.

**Methods:**

A facility-based cross-sectional study was carried out among pregnant women attending antenatal care (ANC) at a primary healthcare facility in Kumasi. Data were collected using an interviewer-administered questionnaire. The primary outcome of the study, FoC, was assessed using the English version of the Wijma Delivery Expectation/Experience Questionnaire (W-DEQ). Additional data included sociodemographic and obstetric characteristics. Stratified multivariable linear regression models were applied to identify parity-specific determinants among the participants.

**Results:**

Of the 282 women who participated in the study, 22% had high-to-severe FoC. Nulliparous women (median: 46.0, IQR: 37.0–66.0) reported significantly higher median W-DEQ scores than parous women (median: 39.0, IQR: 32.0–58.5). In a parity-stratified analysis, among nulliparous women, factors such as a history of family abuse (adjusted Β = 16.32, 95% CI: 2.50 to 30.14) and late ANC booking (adjusted Β = 13.13, 95% CI: 1.43 to 24.84) were significantly associated with FoC. Among parous women, factors such as secondary education (adjusted Β = −7.63, 95% CI: −14.88 to −0.37), intimate partner violence (adjusted Β = 10.87, 95% CI: 0.69 to 21.04), unplanned pregnancy (adjusted Β = 8.38, 95% CI: 2.51 to 14.26), and strong family support (adjusted Β = −27.94, 95% CI: −38.77 to −17.11) were significantly associated with FoC.

**Conclusion:**

FoC is common among this cohort of pregnant women, and its determinants differed by parity and across parity-specific factors. The parity-stratified determinants underscore the need for parity-tailored psychosocial care at ANC, incorporating targeted screening and support procedures aligned with parity-associated risk pathways to enhance maternal well-being.

## Introduction

Fear of childbirth (FoC) is a well-documented psychological phenomenon affecting both maternal and infant health outcomes worldwide [1]. It involves persistent feelings of anxiety, worry, and psychological distress related to pregnancy, labour, and childbirth. It may manifest through emotional symptoms (e.g., mood swings, somatisation) and cognitive effects, including impaired decision-making [2–4]. FoC is associated with adverse consequences, including increased risk of maternal depressive symptoms, avoidance behaviours such as preference for caesarean section without medical indication, and undesirable birth outcomes [5–9]. A systematic review estimated the prevalence of FoC ranges between 4% and 43%, with an overall pooled prevalence of 14% worldwide [4]. Among pregnant women, FoC is influenced by an interplay of sociodemographic, psychosocial, and obstetric factors, including younger age, lower educational attainment, nulliparity, previous obstetric complications, and lack of social support [6,9,10]. While nulliparous women often report higher FoC due to inexperience and uncertainty, parous women may develop fears related to adverse past birth experiences [4,6].

Fear of childbirth remains a significant antenatal concern in sub-Saharan Africa, with prevalence studies from nearby countries reporting similar rates [9,11,12]. However, data on the prevalence and determinants of FoC in Ghana remain limited, and existing evidence suggests that cultural, mental health (e.g., anxiety and depression), and obstetric factors significantly shape women’s childbirth experiences [11]. Furthermore, the specific factors associated with FoC within parity groups remain underexplored. Although previous studies have highlighted the role of parity in shaping FoC [10,12], few have directly assessed parity-specific determinants in the region. Most of these studies either report prevalence separately or treat parity merely as a covariate in multivariable analyses [9,12–14], potentially masking unique risk profiles.

Notably, considering parity only as an adjustment variable may mask unique obstetric and psychosocial pathways by which FoC emerges in first-time mothers compared with women with previous birth experiences. This is because the factors contributing to FoC may differ across these parity groups, potentially limiting a more nuanced understanding of risk profiles and hindering the design of patient-centred antenatal counselling strategies. To the best of the authors’ knowledge, no study in Ghana has identified parity-stratified risk factors among nulliparous and parous women. This methodological approach provides an opportunity for identifying within-group risk factors to inform routine antenatal care (ANC) screening in resource-limited primary care settings.

Therefore, a stratified approach is essential to determine whether socio-demographic, obstetric, and psychosocial factors differ by parity, as first-time mothers and those with previous birth experiences may require different antenatal support or interventions. To address these gaps, we aimed to assess the prevalence of FoC and identify parity-specific determinants among ANC attendees in a primary healthcare facility in Kumasi, Ghana. Identifying parity-specific determinants may provide guidance for targeted psychosocial care and structured screening within routine ANC to reduce the magnitude of FoC and improve maternal well-being.

## Methods

### Study design, setting, and population

A facility-based cross-sectional study was conducted among pregnant women attending ANC at HopeXchange Medical Centre in Santasi, Kumasi, between January 20, 2025, and March 12, 2025. Santasi is a suburban area of Kumasi, the capital of the Ashanti Region of Ghana. HopeXchange Medical Centre is a faith-based health facility with the Christian Health Association of Ghana (CHAG). It is a network of Christian faith-based health facilities and health training institutions that works in partnership with the Ministry of Health to provide health services in Ghana. The facility provides comprehensive general and specialised medical services. The ANC clinic operates from Monday to Friday and is staffed by two doctors and ten midwives. Upon arrival, pregnant women present their ANC booklets and hospital cards at the records unit, after which they are assigned to a midwife for routine consultations. Physician consultations are provided as needed. The facility provides services to women from Santasi and neighbouring communities, and given its faith-based service profile, may receive clients from outside the immediate locality. The facility serves an antenatal population of approximately 5000 women. In 2024, the facility recorded more than 600 deliveries. The facility was selected as the study site due to its established ANC service, the number of pregnant women accessing care and suitability for recruitment within the defined study period. Importantly, the facility offers a suitable setting for assessing FoC among pregnant women accessing ANC services within a faith-based healthcare context in Kumasi.

The study population comprised all pregnant women who received ANC at the clinic during the study period. The inclusion criteria were pregnant women of any gestational age who were willing to provide informed consent. The exclusion criteria were pregnant women who were critically ill and required hospitalisation at the time of data collection.

The study included all pregnant women irrespective of parity. The study aimed to identify factors associated with FoC in the overall population and to conduct a parity-stratified analysis. This approach enables the identification of both general and parity-specific risk factors to guide targeted ANC counselling and interventions.

### Sample size estimation

The sample size was estimated using the single population proportion formula [13]: n = Z²P(1 − P)/E². Where *Z* represents the standard normal value at a 95% confidence interval (1.96), *E* is the error margin (0.05), and *P* is the estimated proportion of pregnant women with FoC (22%) from a previous study [14]. This yielded an initial sample size of 264. After adjusting for a 10% non-response rate, the final required sample size was 290 pregnant women.

### Recruitment procedure

Participants were recruited using a simple random sampling technique from a daily attendance list of all pregnant women who had registered for ANC by 10:00 am at the records unit. This time cut-off was operationally realistic, as a feasibility assessment conducted prior to data collection indicated that over 90% of attendees had registered by then. Unique identifiers were assigned, and participants were randomly selected using a lottery method without replacement. Selected pregnant women were contacted individually by research staff to determine their eligibility for inclusion in the study. This process was repeated daily until the estimated sample size was achieved (over 24 days, with an average recruitment of 12 participants per day).

### Data collection tools and procedures

Data collection was conducted by five research assistants, comprising three midwives and two physician assistants. Prior to data collection, the research assistants participated in a two-day training workshop facilitated by a biostatistician and the principal investigator. The training covered key areas, including quantitative data collection, the consent process, and the administration of the data collection tool.

An interviewer-administered questionnaire was used to collect data for the study. The questionnaire was developed after a review of relevant literature [15–21]. It was structured into three parts: 1) demographic characteristics, 2) clinical factors, and 3) FoC. The demographic variables included age, marital status, educational level, occupation, religion, head of household, perceived family support, experiencing intimate partner violence (IPV), and history of abuse in the family. These three psychosocial variables were measured using single-item self-report questions to reduce respondents’ burden and enhance clarity, given their sensitive nature in an ANC setting. Additionally, the clinical factors included ANC booking, whether the decision regarding ANC booking was influenced by another person, whether the current pregnancy was planned, parity, and history of abortion. ANC booking was categorised as late and early bookings, with late booking defined as initiation of ANC after the first trimester (>12 weeks of gestation) [22].

FoC, the primary outcome of the study, was measured with the English version of the Wijma Delivery Expectation/Experience Questionnaire (W-DEQ) [23]. The W-DEQ is a validated 33-item scale designed to measure women’s experiences or expectations of childbirth [23]. Each item is rated on a 5-point Likert scale with total scores ranging from 0 to 165. Although the W-DEQ is a continuous scale, prevalence was defined using a literature-based cut-off for the overall scores: low (≤ 37), moderate (38–65), high (66–84), or severe (≥ 85) levels of FoC [23]. Participants classified within the high or severe categories (≥66) were defined as FoC cases [24–26]. The W-DEQ has been widely used in other settings to measure FoC among pregnant women [11,20,27,28]. In the current study, the scale demonstrated high internal consistency (Cronbach's alpha = 0.975).

The questionnaire was pretested with 20 pregnant women at a similar health facility in another town. The data collected were analysed and used to amend the questionnaire before it was used in the final study. Specifically, the amendment affected the order of the questions, the rewording of ambiguous items, and the inclusion of a preamble to ensure participants understood the questions.

### Statistical analysis and data quality management

Data were exported to Stata version 16 for quality checks, coding, and statistical analysis. Descriptive statistics, such as frequencies and percentages, were used to describe categorical variables, while means with standard deviations and medians with interquartile ranges were used to describe continuous data, where applicable. Parity-stratified comparisons between demographic and clinical characteristics were examined using the chi-square test and independent sample t-test, where necessary. For descriptive purposes, the W-DEQ score, measured as a continuous variable, was categorised into three FoC levels. Severity categories were created and reported descriptively to provide a clinically meaningful context to evaluate the distribution of FoC levels, instead of being used for modelling or subgroup comparison. However, a multivariable linear regression analysis was used to identify factors associated with FoC using continuous W-DEQ scores. Variables with p-values < 0.20 in bivariable analyses, together with clinically and theoretically relevant variables identified from existing literature, were included in the multivariable models. We adopted this approach to ensure that both statistically significant and contextually important variables were considered in the adjusted models. To determine whether the factors associated with FoC differed by parity, stratified multivariable linear regression models were fitted to identify factors that were independently associated with nulliparous and parous women. We set the statistical significance threshold at p < 0.05, corresponding to a 95% confidence interval. Linear regression assumptions were assessed using residual diagnostics and were found to be adequately satisfied. The Variance Inflation Factor (VIF) was used to assess multicollinearity among independent variables for all the multivariable models (overall, nulliparous-specific, and parous-specific). The mean VIFs for the overall, nulliparous-specific, and parous-specific models were 2.44 (range: 1.17 to 4.80), 2.94 (range: 1.48 to 6.86), and 3.02 (range: 1.10 to 6.97), respectively. All mean VIFs were below the conventional threshold of 10, indicating no significant multicollinearity. However, some individual variables demonstrated relatively high VIF values. Specifically, in the nulliparous-specific model, tertiary education had a VIF of 6.86, and in the parous-specific model, occupation (formal: 6.97; informal: 6.08) had high VIFs. These variables were retained in the models due to their theoretical significance and their minimal to no effect on estimates of other variables.

### Ethical approval

This study was conducted in accordance with the ethical principles outlined in the Declaration of Helsinki. The Committee on Human Research, Publications, and Ethics (CHRPE), School of Medicine and Dentistry, Kwame Nkrumah University of Science and Technology (KNUST), Kumasi (CHRPE/AP/025/25) granted ethical approval for this study. Written permission was obtained from the management of the study site. Before recruitment, all study participants went through a mandatory informed consent process and signed an informed consent form. As part of the consent process, all study participants were provided with information about the study's objectives, significance, and potential risks of participation. One of the researchers (JOA) is employed at the study site. However, the researcher’s affiliation with the facility did not influence the conduct, analysis, interpretation or outcome of the study.

## Results

### Socio-demographic and clinical characteristics of the study participants stratified by parity status

Of the 290 women recruited, 282 participated in the study, yielding a response rate of 97.2%. Of these participants, 35.1% were nulliparous, while the remaining 64.9% were parous. The overall mean age of the study participants was 28 (±5.5) years, with nulliparous women (23.9 ± 3.9 years) being significantly younger than parous women (30.2 ± 5.0 years). Approximately 45.5% of the nulliparous women were single, compared to 8.7% of the parous women. While both groups had relatively high levels of tertiary education, parous women had a slightly higher level (48.1%) than nulliparous women (45.6%). Approximately half (50.8%) of parous women had an informal occupation, compared to 24.2% of nulliparous women. The level of family support was similar across the two groups of women (Table 1).

**Table 1:** Socio-demographic and clinical characteristics of the study participants stratified by parity.

Characteristics	Parity		Overall, N = 282	p-value
	Nulliparous, N = 99 (35.1)	Parous, N = 183 (64.9)		
Age (mean ±SD) ^*1*^	23.9 (±3.9)	30.2 (±5.0)	28 (±5.5)	**<0.001**
**Marital status**				**<0.001**
Single	45 (45.5)	16 (8.7)	61 (21.6)	
Married	38 (38.4)	126 (68.9)	164 (58.2)	
Co-habiting	16 (16.2)	41 (22.4)	57 (20.2)	
**Education level**				**0.004**
Basic	9 (9.1)	41 (22.4)	50 (17.7)	
Secondary	45 (45.5)	54 (29.5)	99 (35.1)	
Tertiary	45 (45.6)	88 (48.1)	133 (47.2)	
**Occupation**				**<0.001**
Unemployed	39 (39.4)	9 (4.9)	48 (17.0)	
Formal	36 (36.4)	81 (44.3)	117 (41.5)	
Informal	24 (24.2)	93 (50.8)	117 (41.5)	
**Religion**				0.397
Christian	77 (77.8)	150 (82.0)	227 (80.5)	
Muslim	22 (22.2)	33 (18.0)	55 (19.5)	
**Head of household**				**<0.001**
Partner	71 (71.7)	177 (96.7)	248 (87.9)	
Self	9 (9.1)	6 (3.3)	15 (5.3)	
Mother/Father	19 (19.2)	0 (0.0)	19 (6.7)	
**Experienced IPV**				0.056
Yes	9 (9.1)	32 (17.5)	41 (14.5)	
No	90 (90.9)	151 (82.5)	241 (85.5)	
**History of abuse in the family**				0.585
Yes	22 (22.2)	46 (25.1)	68 (24.1)	
No	77 (77.8)	137 (74.9)	214 (75.9)	
**ANC booking (weeks)**				0.384
Early [1–12]	71 (71.2)	122 (66.7)	193 (68.4)	
Late [13]+)	28 (28.3)	61 (33.3)	89 (31.6)	
**ANC booking depends on partner’s decision**				0.260
Yes	21 (21.2)	29 (15.9)	50 (17.7)	
No	78 (78.8)	154 (84.2)	232 (82.3)	
**Planned the current pregnancy**				0.493
Yes	45 (45.5)	91 (49.7)	136 (48.2)	
No	54 (54.6)	92 (50.3)	146 (51.8)	
**History of abortion**				**0.001**
Yes	36 (36.4)	34 (18.6)	70 (24.8)	
No	63 (63.6)	149 (81.4)	212 (75.2)	
**Family support**				0.746
Poor	25 (25.3)	40 (21.9)	65 (23.1)	
Moderate	33 (33.3)	68 (37.2)	101 (35.8)	
Strong	41 (41.4)	75 (41.0)	116 (41.1)	

^*1*^Continuous variables reported as mean (±SD); all others as n (%).

### Prevalence of FoC among study participants

**Fig 1:**
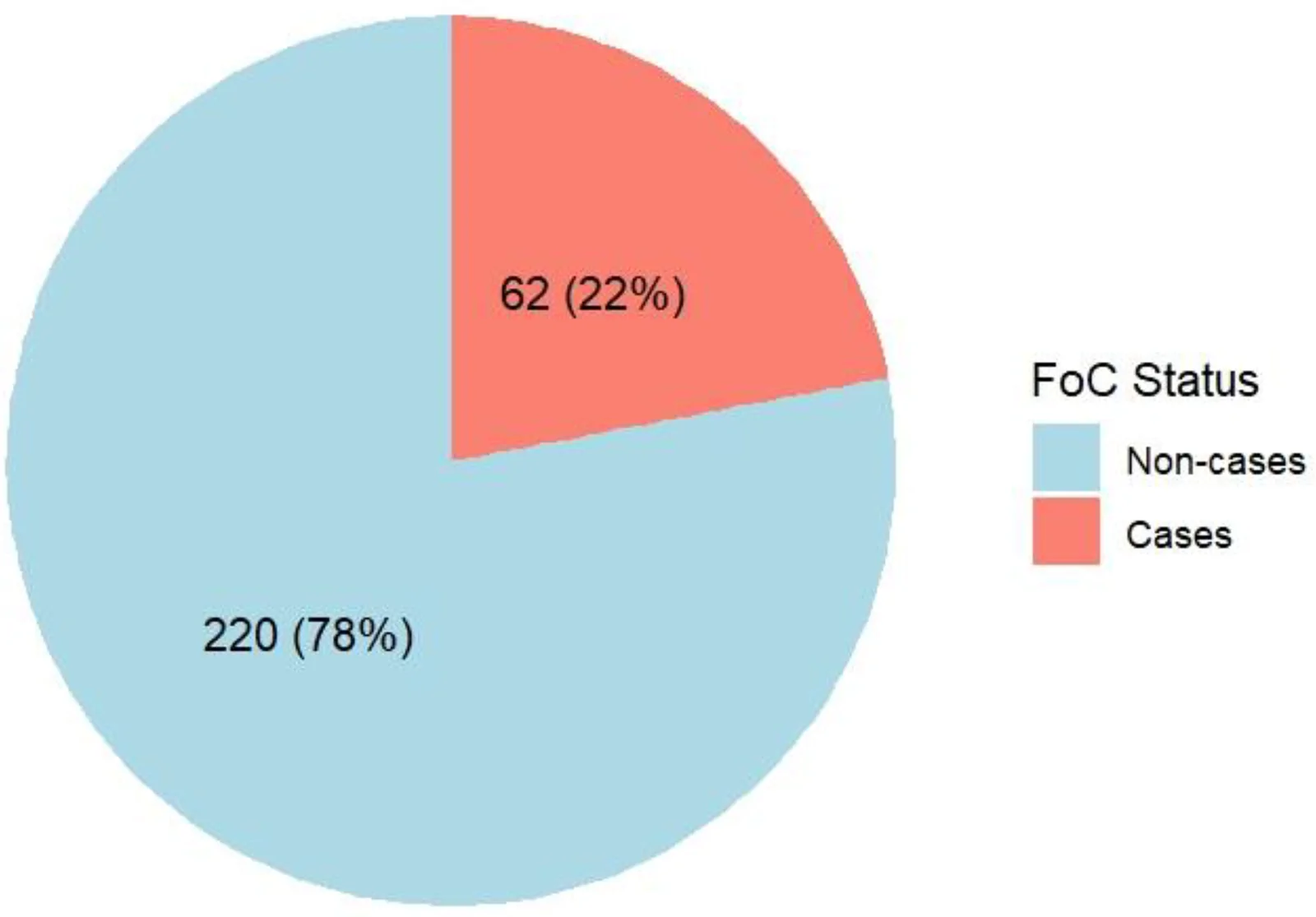
Prevalence of FoC among study participants.

Overall, 22.0% of the participants had high-to-severe FoC (W-DEQ score ≥66). (Fig 1). The median W-DEQ score among the study participants was 41 (interquartile range [IQR]: 33–61). When stratified by parity, nulliparous women (median: 46.0, IQR: 37.0–66.0) reported significantly higher median W-DEQ scores than parous women (median: 39.0, IQR: 32.0–58.5) (p = 0.004). Regarding the severity of FoC, nulliparous women reported significantly higher levels of FoC compared to parous women, with a greater proportion experiencing severe fears (16.2% vs 10.4%) (Table 2).

**Table 2:** Prevalence of FoC among study participants stratified by parity.

Characteristics	Parity		Overall, N = 282	p-value
	Nulliparous, N = 99	Parous, N = 183		
**W-DEQ**				
Median (IQR)	46.0 (37.0–66.0)	39.0 (32.0–58.5)	41 (33–61)	**0.004**
**Levels of FoC, n (%)**				
Low	28 (28.3)	83 (45.4)	111 (39.4)	**0.039**
Moderate	45 (45.5)	64 (35.0)	109 (38.7)	
High	10 (10.1)	17 (9.3)	27 (9.6)	
Severe	16 (16.2)	19 (10.4)	35 (12.4)	

IQR: Interquartile range.

### Factors associated with FoC among study participants

In the unadjusted model, factors such as age (β **=** −1.25, 95% CI: −1.74 to 0.77), being married (β **=** −27.13, 95% CI: −33.27 to −20.98), having formal employment (β **=** −25.34, 95% CI: −32.72 to −17.95), experiencing IPV (β **=** 29.42, 95% CI: 22.26 to 36.57), booking ANC late (β **=** 20.82, 95% CI: 15.32 to 26.33), not planning current pregnancy (β **=** 25.71, 95% CI: 20.99 to 30.43) and strong family support (β **=** −36.27, 95% CI: −42.18 to −30.36) were significantly associated with FoC. In the adjusted model, factors such as being in a cohabiting relationship (adjusted Β **=** −8.74, 95% CI: −15.23 to −2.25), having secondary (adjusted Β **=** −7.58, 95%CI: −13.58 to −1.58) and tertiary education (adjusted Β **=** −9.44, 95% CI: −17.65 to −1.23), having informal occupation (adjusted Β **=** −7.88, 95% CI: −14.83 to −0.92), having history of abuse in the family (adjusted Β **=** 10.38, 95% CI: 4.47 to 16.28), late ANC booking (adjusted Β **=** 6.32, 95% CI: 1.67 to 10.96), not planning the current pregnancy (adjusted Β **=** 8.84, 95% CI: 3.72 to13.96) and having moderate (adjusted Β **=** −13.10, 95% CI: −18.70 to −7.50): and strong (adjusted Β **=** −19.87, 95% CI: −26.53 to −13.22) family support remained significantly associated with FoC after adjusting for other covariates (Table 3).

**Table 3:** Linear regression analysis of factors associated with FoC among study participants.

Characteristics	β (95% CI)	p-value	Adjusted β (95% CI)	p-value
Age	−1.25 (−1.74, 0.77)	**<0.001**	−0.35 (−0.86, 0.16)	0.182
**Parity**				
Nulliparous	1.00		1.00	
Parous	−7.78 (−13.80, −1.75)	**0.012**	−3.35 (−8.81, 2.11)	0.228
**Marital status**				
Single	1.00		1.00	
Married	−27.13 (−33.27, −20.98)	**<0.001**	3.35 (−10.03, 3.33)	0.324
Co-habiting	−8.02 (−15.56, −0.47)	**0.038**	−8.74 (−15.23, −2.25)	**0.009**
**Education level**				
Basic	1.00		1.00	
Secondary	−11.94 (−19.08, −4.80)	**0.001**	−7.58 (−13.58, −1.58)	**0.014**
Tertiary	−29.65 (−36.48, −22.82)	**<0.001**	−9.44 (−17.65, −1.23)	**0.024**
**Occupation**				
Unemployed	1.00		1.00	
Formal	−25.34 (−32.72, −17.95)	**<0.001**	−5.65 (−12.61, 1.31)	0.111
Informal	−9.89 (−17.28, −2.51)	**0.009**	−7.88 (−14.83, −0.92)	**0.027**
**Religion**				
Christian	1.00		1.00	
Muslim	4.39 (−2.66, 11.44)	0.221	−3.50 (−8.73, 1.74)	0.190
**Experienced IPV**				
No	1.00		1.00	
Yes	29.42 (22.26, 36.57)	**<0.001**	6.59 (−0.64, 13.82)	0.074
**History of abuse in the family**				
No	1.00		1.00	
Yes	26.51 (20.75, 32.27)	**<0.001**	10.38 (4.47, 16.28)	**0.001**
**ANC booking (weeks)**				
Early (1–12	1.00		1.00	
Late [13]+)	20.82 (15.32, 26.33)	**<0.001**	6.32 (1.67, 10.96)	**0.008**
**Planned the current pregnancy**				
Yes	1.00		1.00	
No	25.71 (20.99, 30.43)	**<0.001**	8.84 (3.72, 13.96)	**0.001**
**History of abortion**				
No	1.00		1.00	
Yes	3.37 (−3.11, 9.84)	0.307	−2.14 (−6.82, 2.54)	0.369
**Family support**				
Poor	1.00		1.00	
Moderate	−20.44 (−26.50, −14.38)	**<0.001**	−13.10 (−18.70, −7.50)	**<0.001**
Strong	−36.27 (−42.18, −30.36)	**<0.001**	−19.87 (−26.53, −13.22)	**<0.001**

β = unstandardized regression coefficient; CI = confidence interval; 1:00 = reference category; Adjusted. β: adjusted regression coefficient.

In the parity-stratified analysis, among nulliparous women, factors such as having a history of family abuse (adjusted Β **=** 16.32, 95% CI: 2.50 to 30.14) and late ANC booking (adjusted Β **=** 13.13, 95% CI: 1.43 to 24.84) were significantly associated with FoC. Among parous women, factors such as having secondary education (adjusted Β **=** −7.63, 95% CI: −14.88 to −0.37), experiencing IPV (adjusted Β **=** 10.87, 95% CI: 0.69 to 21.04), not planning current pregnancy (adjusted Β **=** 8.38, 95% CI: 2.51 to 14.26), and having moderate (adjusted Β **=** −23.10, 95% CI: −31.91 to −14.30) and strong (adjusted Β **=** −27.94, 95% CI: −38.77 to −17.11) family support were significantly associated with FoC (Table 4).

**Table 4:** Stratified multivariable linear regression analysis of factors associated with FoC by parity status.

Characteristics	Nulliparous		Parous	
	Adjusted β (95% CI)	p-value	Adjusted β (95% CI)	p-value
Age	−0.69 (−3.00, 1.62)	0.556	−0.10 (−0.62, 0.42)	0.714
**Marital status**				
Single	1.00		1.00	
Married	−8.06 (−18.61, 2.49)	0.133	−0.96 (−10.94, 9.02)	0.850
Co-habiting	−4.34 (−14.40, 5.71)	0.393	−7.36 (−17.72, 3.01)	0.163
**Education level**				
Basic	1.00		1.00	
Secondary	−12.16 (−32.65, 8.33)	0.241	−7.63 (−14.88, −0.37)	**0.039**
Tertiary	−16.91 (−40.83, 7.01)	0.164	−8.54 (−20.72, 3.17)	0.152
**Occupation**				
Unemployed	1.00		1.00	
Formal	−1.24 (−10.59, 8.10)	0.792	−4.41 (−13.14, 4.33)	0.321
Informal	−3.27 (−13.79, 7.25)	0.538	−7.48 (−15.24, 0.29)	0.059
**Experienced IPV**				
No	1.00		1.00	
Yes	4.83 (−14.56, 24.22)	0.622	10.87 (0.69, 21.04)	**0.037**
**History of abuse in the family**				
No	1.00		1.00	
Yes	16.32 (2.50, 30.14)	**0.021**	6.22 (−1.67, 14.11)	0.121
**ANC booking (weeks)**				
Early [1–12]	1.00		1.00	
Late [13]+)	13.13 (1.43, 24.84)	**0.028**	3.20 (−2.17, 8.58)	0.241
**Planned the current pregnancy**				
Yes	1.00		1.00	
No	−3.69 (−15.07, 7.69)	0.521	8.38 (2.51, 14.26)	**0.005**
**History of abortion**				
No	1.00		1.00	
Yes	−4.05 (−12.36, 4.26)	0.335	4.56 (−1.75, 10.87)	0.155
**Family support**				
Poor	1.00		1.00	
Moderate	5.06 (−6.32, 16.44)	0.379	−23.10 (−31.91, −14.30)	**<0.001**
Strong	−9.06 (−19.52, 1.39)	0.088	−27.94 (−38.77, −17.11)	**<0.001**

β = unstandardized regression coefficient; CI = confidence interval; 1:00 = reference category; Adjusted β: adjusted regression coefficient.

## Discussions

This study sought to examine the prevalence and determinants of FoC among pregnant women attending ANC in a primary healthcare facility in Kumasi, Ghana, with a specific focus on parity (i.e., differences between nulliparous and parous women). We found that FoC is relatively common, with nulliparous women reporting higher fears than parous women, and approximately 22% experiencing high to severe FoC. Our findings highlight a critical need to screen for FoC during routine ANC, especially in settings with limited psychological support.

We found that FoC was substantial in this cohort. Our finding that approximately 22% of the participants experienced high to severe FoC (W-DEQ scores ≥66) is consistent with studies from Malawi (20%) and Australia (24%) [11,29], but lower than the rates reported in Kenya (30.1%), China (35.8%), and the Netherlands (33.9%) [10,25,30]. Additionally, the median W-DEQ score in our study was 41, comparable to a similar survey in Malawi (mean: 47), but lower than what has been documented in high-income countries [21,31,32]. However, direct comparison should be interpreted cautiously because the Malawi study reported mean rather than median scores. This variability may reflect differences in population characteristics, healthcare systems, cultural perceptions of childbirth, availability of psychosocial support, and methodological disparities, including skewness in the distribution of W-DEQ scores. This suggests that the FoC experience is not uniform and varies across populations.

Nulliparous women experienced significantly higher fears than parous women, consistent with a previous study conducted in Finland and other low- and middle-income countries [33–35]. A plausible explanation could be that first-time mothers lack previous birth experiences, leading to uncertainty and perceived higher pain risk. In contrast, parous women, having experienced childbirth, may develop coping mechanisms that reduce the fear.

Consistent with previous research, our findings demonstrate that nulliparous women were more likely to be younger, single, and experiencing childbirth for the first time [36]. These characteristics have been consistently associated with increased FoC. For instance, Duong *et al.* found that nulliparous women tend to have greater FoC due to the unpredictability and novelty of the experience [37]. Additionally, the association between a history of family abuse and higher FoC observed in our nulliparous subgroup is supported by earlier studies by Goldstein *et al.* and Van Parys *et al*. [38,39]. Addressing abuse-related trauma through antenatal counselling represents a critical intervention point to mitigate FoC. Another key finding in nulliparous women was the association between late ANC booking and increased FoC. Late ANC booking may limit access to early interventions, including education and support that could alleviate these fears. Research suggests that nulliparous women who engage in timely ANC are better prepared for childbirth, which subsequently helps to reduce their fear [40].

Among parous women, our findings revealed that higher educational attainment, particularly completion of secondary education, is associated with lower levels of FoC than a basic education, consistent with studies from Iran and Turkey [41,42]. This trend suggests that education enhances knowledge, confidence, and facilitates communication with healthcare providers [43–45]. The observed association between IPV, unplanned pregnancy, and FoC among parous women is well-established [46–49], with significant implications for maternal health. Our study contributes to this body of knowledge by demonstrating that this relationship persists even after adjusting for other factors, underscoring the need for psychosocial support and reproductive counselling.

Family support was inversely associated with FoC, with women reporting moderate and strong family support having significantly lower FoC scores than those reporting poor family support. This finding is consistent with evidence suggesting that social support may help reduce maternal anxiety and childbirth-related stress [50,51]. Women with family support may have emotional reassurance, practical assistance and a sense of security during pregnancy, potentially contributing to greater confidence and reduced fears [52]. Nonetheless, the cross-sectional nature of this study limits causal or temporal inferences. A bidirectional relationship between the two variables is also possible, where women experiencing greater FoC may be more likely to seek or receive support from family members. Hence, although the findings of this study suggest an association between stronger family support and lower FoC, the timing, nature and sufficiency of support may be relevant to understanding this relationship. This may be particularly relevant in the Ghanaian context, where extended family structures and community networks play a crucial role in shaping women’s health experiences. Hence, strengthening appropriate family involvement in ANC may represent a possibly important aspect of psychosocial support for women during pregnancy.

### Implications for practice and research

The findings of this study have important implications for maternal healthcare delivery in Ghana and similar low-resource settings. Our findings highlight the need for parity-specific interventions. Specifically, nulliparous women may benefit from structured antenatal education and early ANC booking, while psychosocial counselling, IPV screening, and family support may be helpful in parous mothers.

Our findings suggest that, in addition to screening for FoC, routine ANC programs should also integrate patient-centred strategies that account for the unique risk profiles within each parity group. Healthcare managers need to train professionals to identify at-risk populations, provide patient-centred education, and involve family members when necessary to promote social support. Incorporating FoC screening tools into routine ANC guidelines could help identify vulnerable women who could benefit from additional psychological or social support strategies, thereby enhancing maternal satisfaction and childbirth preparedness.

Future research should investigate the effectiveness of culturally tailored FoC-reduction interventions, including peer support groups, mobile-based health education tools, and trauma-informed care. Longitudinal studies are also needed to track changes in FoC across pregnancy and the postpartum period and to assess its influence on maternal outcomes.

### Strengths and limitations of the study

A key strength of this study is its stratified analytical approach, which enabled an in-depth comparison of FoC determinants across parity groups. This methodological decision enhances the utility of the findings for developing tailored interventions. The study also employed the W-DEQ, a widely validated tool for assessing FoC, thereby strengthening the study's internal validity.

However, several limitations must be noted. First, the study was conducted in a single primary healthcare facility, which may limit the generalisability of the findings to other regions or facility types in Ghana. Second, some variables, such as IPV, family abuse, and family support, were measured using a single-item self-report, which may reduce measurement breadth and precision that could have been examined with multiple items. Again, participants’ gestational age could have influenced their FoC. However, gestational age was not collected as a study variable and therefore could not be included in the model. Consequently, we acknowledge it as a limitation of the study. Finally, although the sample size was adequate for the primary analyses, it may not have been powered to detect minor effects or subgroup interactions.

Despite these limitations, the study provides valuable insights into the psychological experiences of pregnant women in a resource-constrained setting and identifies clear intervention points to address FoC.

## Conclusion

The current study demonstrates that FoC is a significant psychological burden among the study participants, with approximately one in five women reporting high or severe levels of fear. The determinants of FoC varied by parity: nulliparous women were more affected by abuse and late ANC booking, while parous women’s fear was influenced by education level, IPV, unplanned pregnancy, and perceived family support.

Our findings contribute to understanding FoC in low-resource settings by identifying parity-specific risk profiles that have previously gone unexplored in Ghana. These findings underscore the need for parity-tailored strategies to address FoC during ANC. Routine screening for FoC, early ANC engagement, psychosocial support, and family-inclusive care models may help reduce childbirth-related fear and improve maternal well-being. Future research should explore these strategies using longitudinal, interventional, and qualitative designs to guide policy and practice in Ghana and similar settings.

## Supporting information

S1 Data Datasets used for the analysis. (XLS)

## References

[1] Chen C, Hussein SZB, Nasri NWM, Yao J, Qin Y, Zhao Z, et al. Fear of childbirth among pregnant women: A concept analysis. J Adv Nurs. 2024;80(11):4476–87. doi: 10.1111/jan.16218 38738562

[2] Wigert H, Nilsson C, Dencker A, Begley C, Jangsten E, Sparud-Lundin C, et al. Women’s experiences of fear of childbirth: a metasynthesis of qualitative studies. Int J Qual Stud Health Well-being. 2020;15(1):1704484. doi: 10.1080/17482631.2019.1704484 31858891 PMC6968519

[3] Hofberg K, Ward MR. Fear of pregnancy and childbirth. Postgrad Med J. 2003;79(935):505–10, quiz 508–10. doi: 10.1136/pmj.79.935.505 13679545 PMC1742837

[4] O’Connell MA, Leahy-Warren P, Khashan AS, Kenny LC, O’Neill SM. Worldwide prevalence of tocophobia in pregnant women: systematic review and meta-analysis. Acta obstetricia et Gynecologica Scandinavica. 2017;96(8):907–20. 10.1111/aogs.13138 28369672

[5] Li B, Liu T, Ma D, Sun J, Liu J. Association of fear of childbirth and postpartum depression with perceived partner response during pregnancy. BMC Pregnancy Childbirth. 2025;25(1):211. doi: 10.1186/s12884-025-07332-6 40011837 PMC11863454

[6] Ali MH, Seif SA, Kibusi SM. The influence of fear during pregnancy, labour and delivery on birth outcome among post-delivery women: A case control study in Zanzibar. East Afr Health Res J. 2022;6(2):147–54. doi: 10.24248/eahrj.v6i2.693 36751688 PMC9887506

[7] Elgzar WT, Alshahrani MS, Ibrahim HA-F. Mode of delivery preferences: the role of childbirth fear among nulliparous women. Front Psychol. 2023;14:1221133. doi: 10.3389/fpsyg.2023.1221133 38034315 PMC10687373

[8] Rúger-Navarrete A, Vázquez-Lara JM, Antúnez-Calvente I, Rodríguez-Díaz L, Riesco-González FJ, Palomo-Gómez R, et al. Antenatal Fear of Childbirth as a Risk Factor for a Bad Childbirth Experience. Healthcare (Basel). 2023;11(3):297. doi: 10.3390/healthcare11030297 36766873 PMC9914781

[9] Massae AF, Larsson M, Pembe AB, Mbekenga C, Svanberg AS. Patterns and predictors of fear of childbirth and depressive symptoms over time in a cohort of women in the Pwani region, Tanzania. PLoS One. 2022;17(11):e0277004. doi: 10.1371/journal.pone.0277004 36327253 PMC9632885

[10] Onchonga D, MoghaddamHosseini V, Keraka M, Várnagy Á. Prevalence of fear of childbirth in a sample of gravida women in Kenya. Sex Reprod Healthc. 2020;24:100510. doi: 10.1016/j.srhc.2020.100510 32217359

[11] Khwepeya M, Lee GT, Chen S-R, Kuo S-Y. Childbirth fear and related factors among pregnant and postpartum women in Malawi. BMC Pregnancy Childbirth. 2018;18(1):391. doi: 10.1186/s12884-018-2023-7 30285754 PMC6171200

[12] Berhanu RD, Abathun AD, Negessa EH, Amosa LG. The magnitude and associated factors of childbirth fear among pregnant women attending antenatal care at public hospitals in Ethiopia: a cross-sectional study. BMC Pregnancy Childbirth. 2022;22(1):222. doi: 10.1186/s12884-022-04544-y 35305600 PMC8933614

[13] Charan J, Biswas T. How to calculate sample size for different study designs in medical research?. Indian J Psychol Med. 2013;35(2):121–6. doi: 10.4103/0253-7176.116232 24049221 PMC3775042

[14] Hildingsson I, Haines H, Karlström A, Nystedt A. Presence and process of fear of birth during pregnancy-Findings from a longitudinal cohort study. Women Birth. 2017;30(5):e242–7. doi: 10.1016/j.wombi.2017.02.003 28279636

[15] Phunyammalee M, Buayaem T, Boriboonhirunsarn D. Fear of childbirth and associated factors among low-risk pregnant women. J Obstet Gynaecol. 2019;39(6):763–7. doi: 10.1080/01443615.2019.1584885 31007101

[16] O’Connell MA, Leahy-Warren P, Kenny LC, O’Neill SM, Khashan AS. The prevalence and risk factors of fear of childbirth among pregnant women: A cross-sectional study in Ireland. Acta Obstet Gynecol Scand. 2019;98(8):1014–23. doi: 10.1111/aogs.13599 30821844

[17] Dencker A, Nilsson C, Begley C, Jangsten E, Mollberg M, Patel H, et al. Causes and outcomes in studies of fear of childbirth: A systematic review. Women Birth. 2019;32(2):99–111. doi: 10.1016/j.wombi.2018.07.004 30115515

[18] Räisänen S, Lehto SM, Nielsen HS, Gissler M, Kramer MR, Heinonen S. Fear of childbirth in nulliparous and multiparous women: a population-based analysis of all singleton births in Finland in 1997-2010. BJOG. 2014;121(8):965–70. doi: 10.1111/1471-0528.12599 24494605

[19] He D, Zhang J, Wang G, Huang Y, Li N, Zhu M, et al. Prevalence and factors associated with fear of childbirth in late pregnancy: a cross-sectional study. Front Public Health. 2025;13:1589568. doi: 10.3389/fpubh.2025.1589568 40520265 PMC12162985

[20] Dal Moro APM, Soecki G, de Fraga FS, Petterle RR, Rückl SZ. Fear of childbirth: prevalence and associated factors in pregnant women of a maternity hospital in southern Brazil. BMC Pregnancy Childbirth. 2023;23(1):632. doi: 10.1186/s12884-023-05948-0 37660013 PMC10474709

[21] Lukasse M, Schei B, Ryding EL, Bidens Study Group. Prevalence and associated factors of fear of childbirth in six European countries. Sex Reprod Healthc. 2014;5(3):99–106. doi: 10.1016/j.srhc.2014.06.007 25200969

[22] Oduro CA, Opoku DA, Osarfo J, Fuseini A, Attua AA, Owusu-Ansah E, et al. The burden and predictors of late antenatal booking in a rural setting in Ghana. Nurs Open. 2023;10(4):2182–91. doi: 10.1002/nop2.1467 36330845 PMC10006594

[23] Wijma K, Wijma B, Zar M. Psychometric aspects of the W-DEQ; a new questionnaire for the measurement of fear of childbirth. J Psychosom Obstet Gynaecol. 1998;19(2):84–97. doi: 10.3109/01674829809048501 9638601

[24] Massae AF, Larsson M, Leshabari S, Mbekenga C, Pembe AB, Svanberg AS. Predictors of fear of childbirth and depressive symptoms among pregnant women: a cross-sectional survey in Pwani region, Tanzania. BMC Pregnancy Childbirth. 2021;21(1):704. doi: 10.1186/s12884-021-04169-7 34666696 PMC8524824

[25] Yin A, Shi Y, Heinonen S, Räisänen S, Fang W, Jiang H, et al. The impact of fear of childbirth on mode of delivery, postpartum mental health and breastfeeding: A prospective cohort study in Shanghai, China. J Affect Disord. 2024;347:183–91. doi: 10.1016/j.jad.2023.11.054 38007102

[26] Jing Q, Jie J, Ke X, Liu Y, Xiumin D, Xiuchuan L, et al. Developmental trajectories of maternal fear of childbirth at different times: a prospective longitudinal study. BMC Womens Health. 2025;25(1):325. doi: 10.1186/s12905-025-03717-z 40616047 PMC12226856

[27] Adams SS, Eberhard-Gran M, Eskild A. Fear of childbirth and duration of labour: a study of 2206 women with intended vaginal delivery. BJOG. 2012;119(10):1238–46. doi: 10.1111/j.1471-0528.2012.03433.x 22734617

[28] Alehagen S, Wijma B, Wijma K. Fear of childbirth before, during, and after childbirth. Acta Obstet Gynecol Scand. 2006;85(1):56–62. doi: 10.1080/00016340500334844 16521681

[29] Toohill J, Fenwick J, Gamble J, Creedy DK. Prevalence of childbirth fear in an Australian sample of pregnant women. BMC Pregnancy Childbirth. 2014;14:275. doi: 10.1186/1471-2393-14-275 25123448 PMC4138382

[30] Veringa-Skiba IK, Bruin EI, Mooren B, Steensel FJAV, Bögels SM. Can a simple assessment of fear of childbirth in pregnant women predict requests and use of non-urgent obstetric interventions during labour?. Midwifery. 2021;97(May 2020):102969. 10.1016/j.midw.2021.102969 33691226

[31] Hall WA, Hauck YL, Carty EM, Hutton EK, Fenwick J, Stoll K. Childbirth fear, anxiety, fatigue, and sleep deprivation in pregnant women. J Obstet Gynecol Neonatal Nurs. 2009;38(5):567–76. doi: 10.1111/j.1552-6909.2009.01054.x 19883478

[32] Fenwick J, Gamble J, Nathan E, Bayes S, Hauck Y. Pre- and postpartum levels of childbirth fear and the relationship to birth outcomes in a cohort of Australian women. J Clin Nurs. 2009;18(5):667–77. doi: 10.1111/j.1365-2702.2008.02568.x 19239535

[33] Oladele OC, Akinwaare MO, Ogbeye GB, Oluseye O. Childbirth fear and birth-related mindset of first-time mothers who registered for antenatal care in public hospitals in Nigeria. Int J Childbirth. 2024;:IJC-2024-0027.R1. doi: 10.1891/ijc-2024-0027

[34] Mandar O, Idrees MB, Ahmed A, ALhabardi N, Hassan B, Adam I. Prevalence and associated factors of fear for childbirth among pregnant women in eastern Sudan. J Reprod Infant Psychol. 2023;41(3):319–29. doi: 10.1080/02646838.2021.1995598 34693830

[35] Rouhe H, Salmela-Aro K, Halmesmäki E, Saisto T. Fear of childbirth according to parity, gestational age, and obstetric history. BJOG. 2009;116(1):67–73. doi: 10.1111/j.1471-0528.2008.02002.x 19055652

[36] Geidam A. Pregnancy outcome among nulliparous women at the university of maiduguri teaching hospital, North Eastern Nigeria: A retrospective cohort study. BJMMR. 2014;4(21):3893–901. doi: 10.9734/bjmmr/2014/8399

[37] Duong HT, Hoyt AT, Carmichael SL, Gilboa SM, Canfield MA, Case A. Is maternal parity an independent risk factor for birth defects?. Birth Defects Research Part A: Clinical and Molecular Teratology. 2012;94:230–326. 10.1002/bdra.22889 PMC4476024 22371332

[38] Goldstein BL, Briggs-Gowan M, Grasso DJ. The effects of intimate partner violence and a history of childhood abuse on mental health and stress during pregnancy. J Fam Violence. 2021;36(3):337–46. doi: 10.1007/s10896-020-00149-1 34113060 PMC8186840

[39] Van Parys A-S, Deschepper E, Michielsen K, Galle A, Roelens K, Temmerman M, et al. Intimate partner violence and psychosocial health, a cross-sectional study in a pregnant population. BMC Pregnancy Childbirth. 2015;15:278. doi: 10.1186/s12884-015-0710-1 26554901 PMC4641387

[40] Bektas Pardes B, Guvenc G. The effects of antenatal education and telephone counseling on childbirth fear of nulliparous women and their attitudes toward childbirth: a randomized controlled trial. Rev Assoc Med Bras (1992). 2025;71(1):e20241147. doi: 10.1590/1806-9282.20241147 40105556 PMC11918862

[41] Soysal C, Ulaş Ö, Işıkalan MM, Bıyık İ, Taşçı Y, Keskin N. The changes in fear of childbirth in pregnancy during and before the COVID-19 pandemic. Sci Rep. 2024;14(1):11067. doi: 10.1038/s41598-024-61307-9 38744899 PMC11093970

[42] Barat S, Kordinejad B, Faramarzi M, Khafri S, Bouzari Z, Ebrahim E. Prevalence of fear of childbirth and its effective factors in pregnant women in Babol, Iran (2019- 2020): A cross-sectional study. Int J Women’s Health Reproduc Sci. 2022;11(1):25–32. doi: 10.15296/ijwhr.2023.05

[43] Ketema DB, Leshargie CT, Kibret GD, Assemie MA, Petrucka P, Alebel A. Effects of maternal education on birth preparedness and complication readiness among Ethiopian pregnant women: a systematic review and meta-analysis. BMC Pregnancy Childbirth. 2020;20(1):149. doi: 10.1186/s12884-020-2812-7 32143581 PMC7060625

[44] Amponsah E, Fusheini A, Adam A. Influence of information, education and communication on prenatal and skilled delivery in the Tano North District, Ghana: A cross-sectional study. Heliyon. 2021;7(6):e07245. doi: 10.1016/j.heliyon.2021.e07245 34189302 PMC8215216

[45] Greenaway ES, Leon J, Baker DP. Understanding the association between maternal education and use of health services in Ghana: exploring the role of health knowledge. J Biosoc Sci. 2012;44(6):733–47. doi: 10.1017/S0021932012000041 22377424 PMC3590019

[46] Adu-Bonsaffoh K, Tamma E, Seffah JD. Prevalence and determinants of unintended pregnancy among women receiving antenatal care services: A facility-based cross-sectional study in Ghana. GJHS. 2021;13(9):100. doi: 10.5539/gjhs.v13n9p100

[47] Alain DC, Nidelle TA, Evrard KN, Franck KF, Armand NNW, Sadrack A, et al. Domestic Violence During Pregnancy and Risk of Obstetrical Complications. ACRI. 2024;24(4):142–55. doi: 10.9734/acri/2024/v24i4670

[48] Eliason S, Baiden F, Yankey BA, Awusabo-Asare K. Determinants of unintended pregnancies in rural Ghana. BMC Pregnancy Childbirth. 2014;14:261. doi: 10.1186/1471-2393-14-261 25104039 PMC4132903

[49] Oğurlu M, Erbil N. The effect of intimate partner violence on fear of childbirth among pregnant women. J Interpers Violence. 2023;38(3–4):3737–55. doi: 10.1177/08862605221109915 35876023

[50] Pinar SE, Aksoy OD, Cesur B, Bilgic D, Daglar G, Guler E. Investigation of the relationship between fear of childbirth and social supports of pregnant women in the third trimester in Turkey. Clinical Experimental Obstetr Gynecol. 2017;44:767–71.

[51] Baheiraei A, Mirghafourvand M, Mohammadi E, Charandabi SM-A, Nedjat S. Social support for women of reproductive age and its predictors: a population-based study. BMC Womens Health. 2012;12:30. doi: 10.1186/1472-6874-12-30 22988834 PMC3675417

[52] Acoba EF. Social support and mental health: the mediating role of perceived stress. Front Psychol. 2024;15:1330720. doi: 10.3389/fpsyg.2024.1330720 38449744 PMC10915202

