# Supplementary material for: Fear of childbirth among antenatal care attendees in a primary care setting in Kumasi, Ghana: Prevalence and parity-stratified determinants
Source: PLoS One. 2026 Sep 21;21(9):e0358457. doi: 10.1371/journal.pone.0358457 (PMC13592637; doi:10.1371/journal.pone.0358457)
Supplement: Supplementary file 1 [file pone.0358457.s010.pdf]

AUTHORS' RESPONSES TO REVIEWER'S COMMENTS

Reviewer's comments	Authors' response
**Reviewer 4**	
The authors have thoroughly addressed the previous reviewers' comments and successfully resolved the technical and methodological issues in the revised manuscript. However some minor typographical errors might have introduced or missed during the revision process and hence suggesting some minor corrections.	We are grateful to you, the other reviewer and the editor for ensuring that the quality and rigour of our manuscript is improved. We strongly believe that your comments have been extremely helpful in improving the quality of the manuscript, and we appreciate them.
**Page 2, Line 34:** using hyphen (-) between identifying and parity-specific determinants "identifying-parity-specific determinants"<br>**Suggested correction:** identifying parity-specific determinants	We are grateful to the reviewer for drawing our attention to this. We statement in reference has been revised and it now reads as;<br><br>*Previous studies on Fear of Childbirth (FoC) across sub-Saharan Africa have typically reported prevalence separately by parity or treated it as a covariate in multivariable analyses, with little attention given to identifying parity-specific determinants*
**Line 34:** Misspelling of "Howerver"	This has been corrected in the revised manuscript, and it now reads as;<br><br>*However, first-time mothers and those with previous birth experiences may require different forms of antenatal support*
**Page 3, Line 67:** worry, and psychological distress related to pregnancy, labor, and delivery.<br>**Suggested correction:** Please replace the word "delivery" with "childbirth" to more accurately reflect the clinical context discussed in this section.	We are grateful to the reviewer for this comment, and in line with that, the statement in reference has been revised to read as;<br><br>*It involves persistent feelings of anxiety, worry, and psychological distress related to pregnancy, labour, and childbirth*
**Page 6, Line 170:** spacing between the word "analyzedand"	This has been done as recommended. It now reads as;<br><br>*The data collected were analysed and used to amend the questionnaire before it was used in the final study.*
