# Supplementary material for: Fear of childbirth among antenatal care attendees in a primary care setting in Kumasi, Ghana: Prevalence and parity-stratified determinants
Source: PLoS One. 2026 Sep 21;21(9):e0358457. doi: 10.1371/journal.pone.0358457 (PMC13592637; doi:10.1371/journal.pone.0358457)
Supplement: Supplementary file 2 [file pone.0358457.s012.pdf]

AUTHORS' RESPONSES TO REVIEWER'S COMMENTS

**Reviewer's comments**	**Authors' response**
**Reviewer 3**	
Overall, this is a valuable and potentially publishable study. Addressing the comments and recommendations provided below would substantially improve the scientific clarity, methodological transparency, and overall quality of the manuscript.	We are grateful to the reviewers for their time, and we believe that their comments were extremely valuable to improving the rigour of the manuscript. We have addressed all comments raised by the reviewers and provided point-by-point responses.
**Main Corrections Made in abstract**	
• Corrected "firsttime" → "first-time"<br>• Improved flow and academic wording<br>• Standardized statistical reporting:<br>• "adj. β" → "adjusted β"<br>• "95%CI" → "95% CI"<br>• Corrected punctuation and spacing<br>• Standardized W-DEQ formatting<br>• Reduced repetition in the conclusion<br>• Improved readability and journal-style tone	We are grateful to the reviewer for these important observations. We have addressed all these comments in the revised manuscript to improve the readability and journal-style tone.
Major Issues to Correct<br>1. Spacing and Formatting Errors<br>There are several missing spaces throughout the manuscript.<br>Examples<br>• "parityspecific" → parity-specific<br>• "singleitem" → single-item<br>• "33item" → 33-item<br>• "nulliparousspecific" → nulliparous-specific<br>• "parousspecific" → parous-specific<br>These should be corrected throughout the document.	We are grateful to the reviewer for these insightful comments. We respectfully note that we corrected all the cited spacing in the previous revision and that it has already been consistently applied throughout the manuscript. We have rechecked the manuscript again to ensure that these corrections remain correctly implemented.
**Introduction**	
Paragraph 1<br>Original<br>It can manifest through emotional symptoms such as mood swings, somatisation, and cognitive effects like impaired decision-making<br>Suggestion<br>It may manifest through emotional symptoms such as mood swings and somatisation, as well as cognitive effects including impaired decision-making.<br>Reason:<br>• More formal academic wording<br>• Better sentence rhythm	We are grateful to the reviewer for these important observations. These sentences have been revised to reflect his/her suggestions.
Paragraph 2<br>Original<br>However, direct prevalence and determinants data for Ghana are limited	We are grateful to the reviewer for these important observations. These sentences have been revised to reflect his/her suggestions.

Suggestion<br>However, data on the prevalence and determinants of FoC in Ghana remain limited.<br>More natural scientific expression.<br>Original<br>cultural and mental health factors – such as anxiety and depression – and obstetric history<br>Suggestion<br>cultural, mental health (e.g., anxiety and depression), and obstetric factors<br>Cleaner academic structure.	
Paragraph 3<br>Original<br>may mask unique obstetric and psychosocial routes<br>Suggestion<br>may mask unique obstetric and psychosocial pathways<br>"Pathways" is more appropriate in epidemiological writing.<br>Original<br>women with previous birth experience<br>Suggestion<br>women with previous birth experiences<br>Grammatically smoother.	We are grateful to the reviewer for these important observations. These phrases have been corrected in the revised manuscript
Final Paragraph<br>Original<br>Identifying parity-specific evidence provides guidance<br>Suggestion<br>Identifying parity-specific determinants may provide guidance<br>More precise, scientifically.	We are grateful to the reviewer for this important observation. This phrase have been corrected in the revised manuscript
Methods	
Study Design Section<br>Original<br>between 20th January 2025 and 12th March 2025<br>Suggested journal style<br>between January 20, 2025, and March 12, 2025<br>Most international journals prefer this format.<br>Original<br>operates Monday to Friday<br>Suggestion<br>operates from Monday to Friday. Minor grammar improvement.	We appreciate the reviewer's observations, and in line with them, the sentences in question have been revised as suggested. Thank you
Sample Size Estimation<br>Important Statistical Issue<br>Original<br>(= (^2 ()(1 −))/^2)<br>Problem<br>The formula notation is not standard.	We appreciate the reviewer's observations, and in line with them, the formula for the sample size estimation has been formatted as suggested. Thank you

Recommended Version<br>Use:<br>n=Z2P(1−P)E2n=E2Z2P(1−P)<br>Or in plain text:<br>The sample size was estimated using the single population proportion formula:<br>$n = Z^2P(1-P)/E^2$<br>Also:<br>• “s” should be “n”<br>• Better mathematical presentation needed for journal publication<br>Recruitment Procedure<br>Excellent overall.	
Small improvement<br>averaging 12 participants per day<br>Could become:<br>with an average recruitment of 12 participants per day	We appreciate the reviewer's observation, and in line with it, the sentence in question has been revised as suggested. Thank you
Data Collection Tools and Procedures<br>Original<br>The investigators developed the questionnaire after reviewing relevant literature<br>Suggestion<br>The questionnaire was developed after a review of relevant literature<br>More concise and formal.	We appreciate the reviewer's observation, and in line with it, the sentence in question has been revised as suggested. Thank you
Important Consistency Issue<br>Original<br>experiencing intimate partner violence (IPV)<br>Later you use:<br>intimate partner violence<br>Better to define abbreviation once and consistently use:<br>• intimate partner violence (IPV)<br>• then IPV afterward	We appreciate the reviewer's observation. We have used ‘IPV’ consistently throughout the manuscript after first defining the abbreviation in the revised manuscript.
Original<br>measured using singleitem self-report questions<br>Correct<br>measured using single-item self-report questions	We are grateful to the reviewer for this observation. We respectfully note that we corrected all the cited spacing in the previous revision and that it has already been consistently applied throughout the manuscript. We have rechecked the manuscript again to ensure that this correction remains correctly implemented.
W-DEQ Section<br>Very strong.<br>Minor Improvement<br>Original<br>with minimum and maximum scores of 0 and 165<br>Suggested	We appreciate the reviewer's observations, and in line with them, the sentences in question have been revised as suggested. Thank you

with total scores ranging from 0 to 165<br>Cleaner wording.	
Original<br>Participants who scored high or severe categories<br>Suggested<br>Participants classified within the high or severe categories	We appreciate the reviewer's observations, and in line with them, the sentences in question have been revised as suggested. Thank you
Statistical Analysis Section<br>This section is strong statistically.<br>Important Clarity Improvement<br>Original<br>We adjusted for both significant ($p < 0.2$) and clinically relevant variables<br>Suggested<br>Variables with p-values < 0.20 in bivariable analyses, together with clinically relevant variables identified from existing literature, were included in the multivariable models.<br>This is more standard in epidemiological reporting.	We appreciate the reviewer's important observation. We have revised the statement in question based on the reviewer's suggestion.
Original<br>theoretical effects on FoC<br>Suggested<br>theoretical relevance to FoC<br>More appropriate wording.	We appreciate the reviewer for this comment. The sentence in question has been revised as;<br><br>*Variables with p-values < 0.20 in bivariable analyses, together with clinically and theoretically relevant variables identified from existing literature, were included in the multivariable models.*
VIF Section<br>This is detailed and transparent, which reviewers usually appreciate.<br>However, this sentence is too long.<br>Original<br>All mean VIFs were below the conventional threshold...<br>Break into two sentences.<br>Suggested<br>All mean VIFs were below the conventional threshold of 10, indicating no substantial multicollinearity. .<br>Then continue with the examples.	We appreciate the reviewer's observations, and in line with them, the sentences in question have been revised as suggested. Thank you
Ethical Approval<br>Strong section.<br>Minor Style Improvement<br>Original<br>Written approval was sought from the study site's management.<br>Suggested<br>Written permission was obtained from the management of the study site.<br>More formal.	We appreciate the reviewer's important observation. We have revised the statement in question based on the reviewer's suggestion.

Important Journal-Level Recommendations<br>1. Add Subheading Consistency<br>Ensure all subheadings follow one style consistently:<br>• Sentence case OR title case<br>Currently mixed.<br>Example:<br>• "Study design, setting, and population"<br>• "Sample size estimation"<br>These are sentence case (good). Keep all the same.	We are grateful to the reviewer for these observations, and in line with that, we have ensured that all subheadings follow one style consistently
2. Clarify ANC Booking Definition<br>You mention:<br>late ANC booking<br>But no operational definition is given.<br>Reviewers may ask:<br>• What gestational age defines "late"?<br>You should define this in Methods.<br>Example:<br>Late ANC booking was defined as initiation of ANC after the first trimester (>12 weeks gestation).<br>(Only if that matches your study definition.)	We are grateful to the reviewer for this comment. We have provided an operational definition for late ANC booking in the revised manuscript. It reads as;<br><br>ANC booking was categorised as late and early bookings, with late booking defined as initiation of ANC after the first trimester (>12 weeks of gestation) (22).
3. Consider Mentioning Model Assumptions<br>Since you used linear regression, reviewers may ask about:<br>• normality<br>• homoscedasticity<br>• residual checks<br>Even one sentence helps:<br>Linear regression assumptions were assessed using residual diagnostics and were adequately satisfied.	We are grateful to the reviewer for these insightful comments. We checked these assumptions and have included a statement in the statistical analysis in the results. It reads as;<br>*Linear regression assumptions were assessed using residual diagnostics and were found to be adequately satisfied*
IMPORTANT ISSUES TO CORRECT<br>1. Remove Line Numbers from the Main Text<br>Examples:<br>• "229"<br>• "230"<br>• "271"<br>• "275"<br>These should not appear inside the manuscript text unless journal submission format specifically requires tracked line numbering.	We appreciate the reviewer's comment. However, the inclusion of continuous line numbers in the manuscript follows the journal's submission requirements. According to the PLOS ONE author guidelines, manuscripts should include both page numbers and continuous line numbering to facilitate the peer-review process. Therefore, the line numbers have been retained in the revised manuscript. Thank you.
2. Table Formatting Problems<br>Several table rows lost formatting.<br>Example:<br>Current<br>Unemployed 1.00 1.00 Formal -1.24...<br>Correct<br>This should appear as separate rows:<br>Occupation Nulliparous Adj. β (95% CI) p-value<br>Parous Adj. β (95% CI) p-value	We are grateful to the reviewer for these comments. We note that the affected variables were already presented on separate rows in the submitted tables. We have reformatted the tables to ensure alignment in the revised manuscript to improve clarity.

Unemployed 1.00 1.00<br>Formal -1.24 (-10.59, 8.10) 0.792 -4.41 (-13.14, 4.33) 0.321<br>Informal -3.27 (-13.79, 7.25) 0.538 -7.48 (-15.24, 0.29) 0.059<br>This formatting issue must be corrected before submission.	
Results	
Opening Paragraph<br>Original<br>A total of 282 of the 290 women recruited participated in the study<br>Suggested<br>Of the 290 women recruited, 282 participated in the study<br>Cleaner journal style.	We appreciate the reviewer's important observation. We have revised the statement in question based on the reviewer's suggestion.
Original<br>Of this, 35.1% were nulliparous<br>Suggested<br>Of these participants, 35.1% were nulliparous<br>Better grammar.	We appreciate the reviewer's important observation. We have revised the statement in question based on the reviewer's suggestion.
Original<br>A little over half (50.8%)<br>Suggested<br>Approximately half (50.8%)<br>More academic wording.<br>Prevalence Section	We appreciate the reviewer's important observation. We have revised the statement in question based on the reviewer's suggestion.
Original<br><br>Overall, the prevalence of FoC among the study participants was 22.0% (≥66)<br><br>Suggested<br><br>Overall, 22.0% of the participants had high-to-severe FoC (W-DEQ score ≥66).<br><br>Much clearer.	We appreciate the reviewer's important observation. We have revised the statement in question based on the reviewer's suggestion.
Original<br>significantly higher median W-DEQ scores<br>Good.<br>However, you should specify the statistical test. Example:<br>...reported significantly higher median W-DEQ scores than parous women (p = 0.004).	We appreciate the reviewer's important observation. We have revised the statement in question based on the reviewer's suggestion.
Table 2 Problems<br>The percentages are incomplete.<br>Example:<br>(28.3)	We thank the reviewer for this observation. We clarify that Table 2 presents categorical variables as *n (%)*, where the frequencies are provided first followed by the corresponding percentages in brackets. We note that this

(45.4)<br>The frequencies are missing.<br>Should be:<br>• 28 (28.3%)<br>• etc.<br>Reviewers will definitely notice this.	format has been consistently applied throughout the table (e.g., "28 (28.3)"), with the header indicating "n (%)" to guide interpretation. To improve clarity, we have reviewed the table formatting to ensure that both frequencies and percentages are presented clearly and consistently aligned across all variables.
Regression Results Section<br>IMPORTANT STATISTICAL REPORTING ISSUE<br><br>Current<br>β: -1.25, 95%CI: -1.74, 0.77<br>This is incorrect because:<br><br>• CI needs "to" or parentheses format<br><br>Correct format: β = -1.25, 95% CI: -1.74 to -0.77 OR<br>β = -1.25 (95% CI: -1.74, -0.77)<br>Currently the upper limit appears positive because of punctuation confusion. This issue appears multiple times.	We are grateful to the reviewer for these observations. We have revised these sections of the manuscript based on the reviewer's suggestions and addressed all the issues and ensured that they are consistent throughout the work. Thank you.
Another Important Error<br>Current: planning current pregnancy (β: 25.71...)<br>But later: not planning the current pregnancy (adj. β: 8.84...)<br>These contradict each other.<br>Likely correction:<br>• unadjusted model should refer to: not planning the current pregnancy<br><br>This inconsistency must be corrected.<br>Family Support Result<br>Current<br>having moderate (adj. β: -13.10, 95%CI: 18.70, -7.50):<br>Incorrect CI order.<br>Should be: adjusted β = -13.10, 95% CI: -18.70 to -7.50<br>Another Grammar Issue	We are grateful to the reviewer for these observations. We have revised these sections of the manuscript based on the reviewer's suggestions.
Current: family's support<br>Use: family support throughout manuscript. Avoid apostrophe.	We are grateful to the reviewer for this observation, and in line with it, we have revised it to "family support". Thank you.
Table 3 Review<br>Very strong overall.<br>Small consistency fixes. Use one reporting style consistently:	We are grateful to the reviewer for the comment, and in line with that, we have used 'adjusted β' consistently throughout the manuscript

Either Adj. β OR adjusted β Choose one style.<br>Current: 1:00 = reference category<br>Correct: 1.00 = reference category	
Table 4 Review<br>Strong scientifically.<br>Grammar issue<br>Current: family support was significantly associated. Plural subject problem.<br>Correct: family support were significantly associated OR better:<br>moderate and strong family support were significantly associated with FoC.	We are grateful to the reviewer for these observations. We have revised these sections of the manuscript based on the reviewer's suggestions. It now reads as; moderate and strong family support were significantly associated with FoC.
**Discussion**	
This section is strong and thoughtful.<br>Opening Paragraph. Excellent summary.<br>Small improvement:<br>Current: underpin a critical need<br>Suggested: highlight a critical need. More natural wording.	We are grateful to the reviewer for these observations. We have revised these sections of the manuscript based on the reviewer's suggestions.
Paragraph Comparing Prevalence<br>Current: median WDEQ score Should be: median W-DEQ score	We appreciate the reviewer for this observation. WDEQ has been written as W-DEQ, but because the spelling extends to the next line, it is appearing as WDEQ. Thank you
Consistency issue. Important Statistical Issue<br>Current: median W-DEQ score in our study was 41, comparable to Malawi (mean: 47) Median and mean are not directly comparable.<br>Suggested:<br>...although direct comparison should be interpreted cautiously because the Malawi study reported mean rather than median scores.<br>Very important for reviewers/statisticians.	We appreciate the reviewer's important observation. We have added a caution in the revised manuscript regarding that comparison and note that it should be interpreted with caution, given that the Malawi study reported mean rather than median scores.
Nulliparous Explanation Paragraph Very good.<br>IPV Paragraph Excellent interpretation.<br>Family Support Paragraph: Scientifically strong but repetitive.<br>You mention: importance of family support, social networks, supportive environments several times.<br>You can shorten by ~25% without losing meaning.	We are grateful to the reviewers for these insightful comments. We have reduced the discussion on the family support paragraph and removed all repetitions without losing meaning.
IMPLICATIONS SECTION<br>Strong practical implications. Grammar Issue<br>Current: they should also integrate patient-centred strategies<br>Problem: Who is “they”?<br>Suggested: routine ANC programs should also integrate patient-centred strategies	We are grateful to the reviewer for this comment. We have revised the statement in question, and it now reads as;<br>Our findings suggest that, in addition to screening for FoC, routine ANC programs should also integrate patient-centred strategies that account for the unique risk profiles within each parity group.
LIMITATIONS SECTION<br>Very good and transparent.	Thank you for your comment

**Conclusion**	
Your conclusion is scientifically sound and appropriately balanced.<br>Main Strength: You avoided overstating causality, which is excellent for a cross-sectional study.<br>IMPORTANT ISSUES TO CORRECT<br>Typographical Error<br>Current: parityspecific risk profiles<br>Correct: parity-specific risk profiles	We appreciate the reviewer's comments. 'Parity-specific' was written as such, but it extends to the next line, and that is why it appears that there was no '–'
Repetitive Wording<br>Current: These findings underscore the need for parity-specific strategies in addressing FoC during ANC.<br>Suggested: These findings underscore the need for parity-tailored strategies to address FoC during ANC.<br>More concise and smoother.	We are grateful to the reviewer for this observation. We have revised the section of the manuscript based on the reviewer's suggestions and removed the repetition.
Slight Overstatement<br>Current: could collectively reduce childbirth-related fear and promote better maternal health outcomes. Since your study is observational, avoid implying intervention effectiveness too strongly. Better: may help reduce childbirth-related fear and improve maternal well-being.	We are grateful to the reviewer for this observation. We have revised the section of the manuscript based on the reviewer's suggestions and removed the repetition
ACKNOWLEDGEMENT REVIEW<br>Major Typographical Error<br>Current: employeesmanagement and staff. This appears to be a copy-paste duplication error.<br>Correct Version: We would like to thank the management and staff of HopeXchange Medical Centre, the study participants, and the research assistants for their valuable contributions to this study.	We are grateful to the reviewer for this observation. We have revised the section of the manuscript based on the reviewer's suggestions and removed the repetition
REFERENCES REVIEW<br>Your references are strong overall, current, and relevant. However, several important formatting and consistency issues need correction.<br>MAJOR REFERENCE ISSUES<br>Duplicate Reference<br>References 11 and 28 are duplicates. Both are: Khwepeya M, Lee GT... You must remove one duplicate reference and renumber accordingly.<br><br>2. Typographical Error in Reference 22 Current: Wjma K.<br>Correct: Wijma K.. Very important because this is the main W-DEQ reference.<br>3. Broken Reference. Reference 48<br>Current:<br>261&partnerID=40&md5=32f6a2614	We are very grateful to the reviewer for these important observations. We have addressed all the issues that were raised by the reviewer, which include removing duplicates, ensuring consistency, capitalisation consistency, etc.<br><br>Regarding the supplementary requirement, it is a journal requirement. According to PLOS ONE, authors are expected to share the data used for the analysis either in a repository or by adding it to the files for submission.

00d8df1c8fb50cd1fad5f96<br>This is clearly accidental database export text and must be deleted.<br>4. Broken Supporting Information Section<br>Current: Datasets used f<br>Incomplete sentence. Correct or remove.<br>Example: Supporting information. The datasets used and/or analysed during the current study are available from the corresponding author upon reasonable request.<br>(Only if true.)<br><br>5. Inconsistent Journal Formatting<br>Examples: "BMC Womens Women's Health" "Front Public Heal." "Women and Birth." "J Obstet Gynaecol (Lahore)." Journal abbreviations should follow one indexing style consistently (usually PubMed/NLM).<br><br>6. Inconsistent Use of Page Ranges<br>Examples: "763– 7", "121–6" Need consistent formatting.<br><br>Example: 763–767, 121–126 depending on journal style.<br><br>7. Capitalization Inconsistency<br>Example: The Influence of Fear During Pregnancy Some references use sentence case, others title case. Most journals require sentence case.<br>Example: Correct: The influence of fear during pregnancy, labour and delivery on birth outcomes among post-delivery women.<br>3. W-DEQ Formatting Always use: W-DEQ Not: WDEQ	
**Reviewer 4**	
I would like to thank the authors for their response and the effort they have put in to revise the manuscript and also they have addressed all the concerns raised in the review.	We are grateful to you for your time and for providing us with comments we believe have strongly enhanced the rigour of our work.
