# Supplementary material for: Fear of childbirth among antenatal care attendees in a primary care setting in Kumasi, Ghana: Prevalence and parity-stratified determinants
Source: PLoS One. 2026 Sep 21;21(9):e0358457. doi: 10.1371/journal.pone.0358457 (PMC13592637; doi:10.1371/journal.pone.0358457)
Supplement: Supplementary file 3 [file pone.0358457.s013.pdf]

AUTHORS' RESPONSES TO REVIEWER'S COMMENTS

Reviewer's comments	Authors' response
**Reviewer 4**	
I have evaluated the clean copy of the revised manuscript and feel that the author have addressed all the issues/concerns raised by the reviewer/ reviewers. I would like to thank the authors for their constructive response and the effort they have put in to revise the manuscript. I feel that the manuscript meets the standards of the journal and hence recommend it for acceptance.	We appreciate the reviewer for these remarks. We believe that the reviewers' comments have helped improve the quality of the work.
There are a few minor font size inconsistencies in line 69,133,134 and 176 (clean copy) which can be resolved in the final proof reading.	We are grateful to the reviewer for drawing our attention to this. We have corrected the inconsistencies in the font size in lines 69, 133, 134, and 176 as suggested.
**Reviewer 5**	
The article provides evidence on associated factors of FoC in a resources-limited setting. The findings may help to improve health care to pregnant women and contribute to reduce maternal mortality. I made some comments that need to be addressed to improve the quality of the manuscript. My main concern is the identification of family support as a risk factor for FoC.	We appreciate the reviewer for these remarks. Particularly, we are also grateful to all reviewers for making time to review this work, as we believe their comments have improved the quality of the manuscript.<br><br>We have addressed all comments raised, including the identification of family support as a risk factor for FoC.
Line 68-69- emotional symptoms such as mood swings and somatisation, as well as cognitive effects, 69 including impaired decision-making (2–4).<br><br>Suggestion - emotional symptoms (e.g., mood swings, somatisation) and cognitive effects including impaired decision-making (2–4).	We are grateful to the reviewer for this suggestion. The sentence in reference has been revised to read as;<br><br>*It may manifest through emotional symptoms (e.g., mood swings, somatisation) and cognitive effects, including impaired decision-making (2–4).*
Line 117 - Complete the description of HopeXchange Medical Centre. 600 deliveries represent 12% of the population and around 50 per month.<br>How many women were received during ANC1 in 2024.? How many cases for curative consultation?<br>This Private not-for-profit surely attracts women outside the estimated population.<br>Are there some reasons why this health facility was selected?	We thank the reviewer for this important comment. We have provided additional useful context for understanding the study setting. However, the facility records available to the research team did not provide a reliable figure for the number of women who registered for ANC1 or the number of curative consultations received in 2024. We have therefore not provided estimates for these indicators to avoid introducing potentially inaccurate information. The statement that portrays 5000 ANC utilisation in 2024 has also been revised as it only refers to estimates for deliveries in 2024
Line 131 … a 10% non-response rate, the final required sample size was 290 pregnant women.<br>Replace with :	We thank the reviewer for this helpful suggestion. Although the estimated sample size after allowing for a 10% non-response

…a 10% non-response rate, the final required sample size was 290 pregnant women (Figure 1).	rate was 290 pregnant women, 282 participants were ultimately recruited and participated in the study. The final number of participants was reported separately in line 222 and is reflected in Figure 1. We therefore believe that adding the citation to Figure 1 to the sample size estimation statement may be misleading, as Figure 1 presents the participant flow based on the 282 women who participated in the study rather than the initially estimated sample size of 290. We have consequently retained the statement in its current form.
Ethical approval<br>Do some researchers work in the HopeXchange Medical Centre facility as health care provider? If yes, the authors should indicate	We are grateful to the reviewer for this comment. Yes, one of the authors works at the study site, and this has been reported in the revised manuscript. It reads as;<br><br>*One of the researchers (JOA) is employed at the study site. However, the reseracher's affiliation with the facility did not influence the conduct, analysis, interpretation or outcome of the study.*
**Page 10**, Figure Prevalence of FoC among study participants<br>- Indicate the number of the Figure (e.g **Figure 1**)<br>- Cite in text<br>- As there are only two items, Remove the legend and insert directly Cases and Non-cases directly in the Figure	We thank the reviewer for this helpful comment. In accordance with the recommendation, the figure has been numbered as Figure 1 and is cited appropriately in the text. We also attempted to incorporate the labels "Cases" and "Non-cases" directly into the figure and remove the legend. However, the direct labelling reduced the clarity and readability of the figure. We have therefore retained the legend to ensure clear interpretation of the figure.
Page 29 - Family support was inversely associated with FoC, consistent with evidence highlighting the protective role of social support against maternal anxiety and childbirth-related stress (50,51). Women with strong support systems tend to feel more empowered, informed, and emotionally secure, which may reduce fears and anxiety (52). This finding underscores the importance of psychosocial supports in shaping childbirth experiences and suggests that integrating family oriented approaches into ANC services could help reduce FoC and improve maternal…<br><br>Comment - This means that by providing family support, women will be more empowered,	We appreciate the reviewer for this comment. Looking at the results again, with poor family support used as the reference category, compared with women reporting poor family support, those reporting moderate family support had lower FoC scores ($\beta = -23.10$, 95% CI: −31.91 to −14.30, $p < 0.001$), while those reporting strong family support also had lower FoC scores ($\beta = -27.94$, 95% CI: −38.77 to −17.11, $p < 0.001$). Thus, our findings indicate an inverse association between family support and FoC, rather than higher FoC among women receiving family support. However, we also agree with the reviewer regarding the potential directionality of this relationship.

informed, and emotionally secure, which may reduce fears and anxiety.<br>This is Iin contradiction with the results showed that women with family support have more FoC. Maybe, there is a bias that family support is mostly provided to women who have already FoC. Therefore, family support will not be considered as an associated factor. Could the authors explain and discuss more this factor?	Importantly, given the cross-sectional design of this study, we can establish that the exposure preceded the outcome. The discussion has been revised based on these. We have subsequently acknowledged them in the revised discussion.
Page 17 - Line 10369 : generalizability. This is US and the authors used the British English. Therefore, the spelling should be **generalisability**	We are grateful to the reviewer for this comment. We have corrected the spelling as suggested.
