# Supplementary material for: Fear of childbirth among antenatal care attendees in a primary care setting in Kumasi, Ghana: Prevalence and parity-stratified determinants
Source: PLoS One. 2026 Sep 21;21(9):e0358457. doi: 10.1371/journal.pone.0358457 (PMC13592637; doi:10.1371/journal.pone.0358457)
Supplement: Supplementary file 4 [file pone.0358457.s002.docx]

**Overall Evaluation**

The manuscript addresses an important maternal-health issue and adds value by examining parity-specific determinants, an angle that is genuinely under-explored in sub-Saharan Africa.

The study is generally well-structured, and the authors make good use of a validated instrument (W-DEQ). The analysis is adequate for a cross-sectional design, and the results are presented clearly.

However, there are several significant issues in methodology, clarity of writing, conceptual framing, organization, and interpretation that need attention before the manuscript reaches publication quality.

**Major Issues**

**Abstract**

- Method- what is the interest of the researcher? Is it to compare between parties? If so, does the researcher consider it in the design stage? It seems at the analysis level. If the researcher thinks that there is a difference in FoC based on parity, why not use a comparative sample?
- Conclusion- the recommendation is not aligned with the result. The determinants were not considered in the recommendation.

**1. Introduction**

- The introduction repeats similar ideas and includes citations that are not always well contextualized.
- The “gap” section is too long and still doesn’t clearly justify why parity-specific analysis is essential for Ghana or how it connects to previous evidence.

**2. Methods**

**Study design, setting, and population**

- Line 103-Satasi vs line 104-Santasi----check it.

**Sample size estimation**

- The sampling frame is unclear. Were all women attending ANC during the study days eligible? What about their time? Are they expected to stay in the clinic until 10:00 am? Is it feasible? It requires strong justification, but it is not convincing.
- Using a list “as of 10:00 a.m.” may introduce selection bias.
- Sample size calculation- the researcher uses proportion, but it is about the mean of Foc. What is the reason for choosing proportion rather than mean?
- It is not clear how many samples were calculated for nullipara and multipara.

**Data collection tools and procedures**

- The researchers mention that the tool was pretested on 20 samples, but didn’t indicate the justification for selecting 20 samples, as most suggested using at least 30 individuals for pretest. There is no information on which aspect of the tool was amended after the preset**.**

**Measurement**

- IPV, family support, and family abuse are measured with single-item questions. This should be acknowledged and justified earlier.

**Statistical analysis**

- The outcome variable is categorized into three, but the researcher used multivariable linear regression. Why not multinomial logistic regression or ordinal logistic regression?
- Authors say they included “significant and clinically relevant variables,” but the criteria are not defined.
- It's unclear whether multicollinearity was checked.
- Important variables like gestational age are missing from the model.

**W-DEQ scoring**

- Severity levels are stated, but the manuscript uses both continuous and categorical outputs. The rationale for mixed reporting should be clarified.

**3. Results**

- Response rate is not indicated in either the abstract or in the document.
- In several places, interpretation borders on causal language despite the cross-sectional design.
- What does the P-value in Table 1 and Table 2 indicate, and what is its importance?

**4. Discussion**

- The discussion is long, sometimes repetitive, and occasionally drifts into general maternal-health topics not directly tied to the findings.
- Some evidence is misinterpreted (e.g., the claim that parous women often report higher FoC contradicts the study’s results and global literature).

**5. Writing & Language**

There are recurring language and style issues:

- Long, multi-clause sentences reduce clarity.
- Occasional typos (e.g., “Wima” instead of “Wijma,” “Satasi” vs “Santasi” variation).
- Inconsistent tense and inconsistent formatting.
- Occasional grammar issues.
