# Supplementary material for: Fear of childbirth among antenatal care attendees in a primary care setting in Kumasi, Ghana: Prevalence and parity-stratified determinants
Source: PLoS One. 2026 Sep 21;21(9):e0358457. doi: 10.1371/journal.pone.0358457 (PMC13592637; doi:10.1371/journal.pone.0358457)
Supplement: Supplementary file 5 [file pone.0358457.s004.docx]

I have reviewed the manuscript entitled *Fear of childbirth in Ghana: Prevalence and within parity-specific determinants among antenatal care attendees in a health facility in Kumasi, Ghana*. The study addresses a relevant research question within the scope of PLOS ONE and presents data that may be of interest to the broader scientific community. The manuscript is generally well structured; however, several issues related to methodological clarity, data analysis, reporting, and language require attention to ensure transparency, reproducibility, and adherence to PLOS ONE standards. The comments below are intended to be constructive and to help strengthen the manuscript.”

***Comments***

*line 1: Fear of childbirth in Ghana: Prevalence and within parity-specific determinants among antenatal care attendees in a health facility in Kumasi, Ghana (“within parity-specific determinants” is grammatically awkward).* ***Suggested correction:***

*Fear of childbirth in Ghana: Prevalence and parity-specific determinants among antenatal care attendees in a health facility in Kumasi*

***ABSTRACT***

***Background***

*Line 34: Previous studies on Fear of Childbirth (FoC) across sub-Saharan Africa have typically reported prevalence separately by parity or treated it as a covariate in multivariable analyses, with little attention given to identifying within-parity-specific determinants (“within-parity-specific” is repetitive and awkward)*
***Suggested correction:***

*with little attention given to identifying parity-specific determinants.*

*line 34-35 : Yet, first-time mothers and those with previous birth experiences may require different forms of antenatal support (“Yet,” at the beginning is acceptable but slightly informal).* ***Suggested correction:***

*However, first-time mothers and those with previous birth experiences may require different forms of antenatal support.*

***Methods***

*line 43 : Stratified multivariable linear regression models were applied separately to identify parity-specific determinants among the participants(Clear, but “applied separately” is redundant)* ***Suggested correction:***

*Stratified multivariable linear regression models were applied to identify parity-specific determinants.*

***Results***

*line 51: independently associated with FOC (Inconsistent capitalization (FoC vs FOC)*

***Suggested correction:***

*independently associated with FoC.*

*(Apply this correction* ***throughout the manuscript****.)*

***Conclusion***

*differed by parity and within parity-specific factors.*

***Comment:*** *line 57 : Redundant and grammatically awkward.*

***Suggested correction:***

*differed by parity and across parity-specific factors.*

***INTRODUCTION***

*line 67 : worry and psychological distress related to pregnancy, labour and delivery(comma consistency)*

***Suggested correction:***

*worry, and psychological distress related to pregnancy, labour, and delivery.*

*Line 68 somatizations (British spelling is acceptable, but be consistent).*

***Suggested correction (optional):***

*somatization*

*line 76 : An interplay of sociodemographic, psychosocial and obstetric factors( Missing comma).*
***Suggested correction:***

*sociodemographic, psychosocial, and obstetric factors*

*Line 88-89 : few of have directly assessed the parity-specific determinants(Grammatical error or missing word).*
***Suggested correction:***

*few have directly assessed parity-specific determinants*

***METHODS***

***Study design***

***Original:***

*Line 113 : Pregnant women who looked critically ill(“looked” is subjective and informal)*

***Suggested correction:***

*Pregnant women who were critically ill*

***Sample size***

*By adjusting for a contingency rate of 10%*

***Comment:*** *line 120 : “contingency rate” is unclear.*

***Suggested correction:***

*By adjusting for a 10% non-response rate*

***Sampling***

*records units
line 125: records units (Plural unnecessary).*

***Suggested correction:***

*records unit*

***Data collection tools***

*Line 159 : The data collected was analyzed(Subject–verb agreement.)*
***Suggested correction:***

*The data collected were analyzed*

*Line 147 : the decision of ANC booking is influenced by who(Sentence fragment / unclear phrasing.)*
***Suggested correction:***

*whether the decision regarding ANC booking was influenced by another person*

***W-DEQ description***

*Line 150-151: 33- item scale(Spacing error)*

***Suggested correction:***

*33-item scale*

***STATISTICAL ANALYSIS***

*Line 165-166 : Parity-stratified comparison between demographic and clinical characteristics(“comparison” should be plural or rephrased)*
***Suggested correction:***

*Parity-stratified comparisons of demographic and clinical characteristics*

*Line 171 : factors that each independently associated(Missing verb).*

***Suggested correction:***

*factors that were independently associated*

***RESULTS***

***Participant characteristics***

*Line 190-191 : parous women (23.9 ± 3.9 years) being significantly older than nulliparous women (30.2 ± 5.0 years) ; (Values are reversed (logical error).*
***Suggested correction:***

*nulliparous women (23.9 ± 3.9 years) being significantly younger than parous women (30.2 ± 5.0 years)*

***Tables***

*ANC booking (weeks) Early (1 – 12 (Missing closing parenthesis.*
***Suggested correction:***

*Early (1–12)*

*38.70 (Inconsistent decimal places and missing parenthesis).*

***Suggested correction:***

*38.7)*

***DISCUSSION***

*Line 277 : skewedness in the distribution(Nonstandard word)*
***Suggested correction:***

*skewness in the distribution*

*Line 292-293 : Compared to nulliparous women, parous women often report higher levels of FoC(This contradicts earlier statements)*
***Suggested correction:***

*Compared to parous women, nulliparous women often report higher levels of FoC*

***IMPLICATIONS***

*Line 332 : prevalence of fear FOC(Typographical error)
.****Suggested correction:***

*prevalence of FoC*

***CONCLUSION***

*Line 377 : Routine screening for FOC(Capitalization inconsistency)*
***Suggested correction:***

*Routine screening for FoC*

***ACKNOWLEDGEMENT***

*Line 384 : Medical Center's management(Spelling consistency with earlier “Centre”)*

***Suggested correction:***

*Medical Centre’s management*

***OVER ALL LANGUAGE ASSESSMENT***

***Strengths***

- *Academic tone is appropriate*
- *Sentence structure is strong*
- *Logical flow is excellent*
- *Journal-ready with minor edits*

***Main issues to fix***

1. *Inconsistent use of* ***FoC vs FOC***
2. *Minor grammatical slips (missing words, spacing)*
3. *A few* ***logic inconsistencies*** *(age, parity comparison)*
4. *Redundant phrasing (“within parity-specific”)*
