# Supplementary material for: Fear of childbirth among antenatal care attendees in a primary care setting in Kumasi, Ghana: Prevalence and parity-stratified determinants
Source: PLoS One. 2026 Sep 21;21(9):e0358457. doi: 10.1371/journal.pone.0358457 (PMC13592637; doi:10.1371/journal.pone.0358457)
Supplement: Supplementary file 6 [file pone.0358457.s005.docx]

**AUTHORS' RESPONSES TO REVIEWER’S COMMENTS**

| **Reviewer’s comments** | **Authors’ response** |
| --- | --- |
| The manuscript addresses an important maternal-health issue and adds value by examining parity-specific determinants, an angle that is genuinely under-explored in sub-Saharan Africa. The study is generally well-structured, and the authors make good use of a validated instrument (W-DEQ). The analysis is adequate for a cross-sectional design, and the results are presented clearly.  However, there are several significant issues in methodology, clarity of writing, conceptual framing, organization, and interpretation that need attention before the manuscript reaches publication quality. | We are very grateful to the reviewer for investing his time to review this work. It is worth noting that the reviewer has provided insightful comments that will enhance the quality of the work.  We have addressed all issues raised by the reviewer in the revised manuscript.  We have provided a point-by-point response to each issue raised by the reviewer below. |
| **Abstract** | |
| Method- what is the interest of the researcher? Is it to compare between parties? If so, does the researcher consider it in the design stage? It seems at the analysis level. If the researcher thinks that there is a difference in FoC based on parity, why not use a comparative sample? | We are grateful to the reviewer for this key observation. Our main aim was to explore the prevalence of FoC and identify within parity-specific determinants among antenatal care attendees. The aim of the stratification was not to compare FoC between the two groups, but rather to identify the factors associated with FoC within each parity group. The rationale for this analysis was that the general interventions which are currently practised at antenatal care may not be generally effective across all parity groups. Hence, understanding the factors associated with FoC within each parity group can inform targeted or specific interventions at antenatal care. We have provided clarity under the **Study Design, Setting And Population** in the revised manuscript.  *The study population consisted of all pregnant women who accessed ANC at the clinic during the study period. Pregnant women who looked critically ill and required hospitalization were excluded from the study. Though the study included all women irrespective of their parity status, the rationale was not to compare the factors associated with FoC between parity groups. This method enables the identification of factors that may vary depending on parity and provides guidance for focused ANC interventions.* |
| Conclusion- the recommendation is not aligned with the result. The determinants were not considered in the recommendation | We appreciate the reviewer's careful observation. The conclusion has been revised to include the determinants, as recommended by the reviewer.  *FoC is common among this cohort of pregnant women, and its determinants differ by parity status. The parity-stratified determinants underscore the need for parity-tailored psychosocial care at ANC, incorporating targeted screening and support procedures aligned with parity-associated risk pathways to enhance maternal well-being.* |
| **Introduction** | |
| The introduction repeats similar ideas and includes citations that are not always well contextualized | We appreciate the reviewer for his insightful comments on the introduction. We have revised the introduction by avoiding the repetition of similar ideas and also ensured that all citations are well contextualized, as recommended by the reviewer. |
| The “gap” section is too long and still doesn’t clearly justify why parity-specific analysis is essential for Ghana or how it connects to previous evidence | The gap section of the manuscript has been revised to clarify the need for the parity-specific analysis and how it connects to the literature. The introduction has been improved and now highlights the evidence gap and rationale for the study. |
| Methods |  |
| **Study design, setting, and population:** Line 103-Satasi vs line 104-Santasi----check it. | We appreciate the reviewer's attention to detail. This is a typo, and we have corrected it in the revised manuscript. The correct spelling is “Santasi” |
| **Sample size estimation:** The sampling frame is unclear. Were all women attending ANC during the study days eligible? What about their time? Are they expected to stay in the clinic until 10:00 am? Is it feasible? It requires strong justification, but it is not convincing. Using a list “as of 10:00 a.m.” may introduce selection bias. Sample size calculation- the researcher uses proportion, but it is about the mean of Foc. What is the reason for choosing proportion rather than mean? It is not clear how many samples were calculated for nullipara and multipara | We are grateful to the reviewer for this observation. We have provided clarity in the revised manuscript. https://drive.google.com/drive/folders/13YKud2GspuPrVqwQqj038R-Fv7393bsp?usp=drive_link  *On each data collection day, a list of all pregnant women who had presented and registered for ANC by 10:00 am was retrieved from the records units. A feasibility assessment conducted prior to data collection found that over 90% of pregnant women attending the clinic had registered and were already in the care process, indicating that the cut-off time was operationally feasible. All attendees typically report to the clinic between 7:00 am and 7:30 am, with clinical services starting at 8:30 am. ANC services usually last between 3 – 4 hours, covering registration, triage, midwife/clinical review, ultrasound scanning, laboratory investigation, and drug dispensing. As a result, by 10:00 am, most attendees were still progressing through the care process. The 10:00 am list was therefore used solely for daily recruitment, after which participants were randomly selected from it. This approach ensured equitable selection of both early and late arrivers across the clinic flow.*  *Again, we aimed to estimate the prevalence and parity-specific determinants of FoC. Hence, the single population proportion formula was used to estimate the required sample size. Although the W-DEQ is a continuous scale, prevalence was defined using a literature-based cut-off for the overall scores: low (≤ 37), moderate (38 – 65), high (66 – 84), or severe (≥ 85) levels of FoC (22). Participants who scored high or severe categories (≥66) were classified as FoC cases (23–25).*  We have provided clarity in the revised manuscript. We have also provided clarity on how many samples were calculated for nullipara and multipara in the revised manuscript. Below are the revised sections in the manuscript.  *The sampling frame comprised all pregnant women who accessed ANC services at the clinic during the study period. On each data collection day, a list of all pregnant women who had presented and registered for ANC by 10:00 am was retrieved from the records units. This cut-off was selected based on prior feasibility assessment showing that over 90% of pregnant women typically report to the clinic by this time, ensuring that the majority of the population is included while handling day-to-day recruitment logistics.*  *Despite measuring FoC as a continuous variable, this formula provides a conservative estimate adequate for prevalence and parity-stratified analysis.*  *We estimated the sample size for the whole cohort, without pre-specifying for nulliparous and parous women. The stratification was done at the analysis stage, enabling parity-specific examination among the recruited sample.* |
| **Data collection tools and procedures:** The researchers mention that the tool was pretested on 20 samples, but didn’t indicate the justification for selecting 20 samples, as most suggested using at least 30 individuals for pretest. There is no information on which aspect of the tool was amended after the preset | We pretested the tool on 20 pregnant women in another facility, with the decision guided by the limited number of pregnant women within the time frame for the pretest. While in the literature some recommend 30 individuals, pretesting on 20 participants is deemed acceptable for identifying issues related to clarity, comprehension and the flow of the tool. We have also provided in the revised manuscript where the amendment was affected in the tool.  *Specifically, the amendment affected the order of the questions, the rewording of ambiguous items, and the inclusion of a preamble to ensure participants understood the questions.* |
| **Measurement:** IPV, family support, and family abuse are measured with single-item questions. This should be acknowledged and justified earlier | We thank the reviewer for these insightful comments. We acknowledge the use of single-item self-report questions to measure IPV, family support, and family abuse. We adopted this approach to reduce the burden on respondents while ensuring that sensitive issues like these could be addressed quickly and clearly at the ANC setting. We also acknowledged it as a limitation in the study.  *These three psychosocial variables were measured using single-item self-report questions to reduce respondents’ burden and enhance clarity, particularly given their sensitive nature in an ANC setting.*  *Second, some variables, such as IPV, family abuse and family support, were measured using a single-item self-report, which may reduce measurement breadth and precision that could have been examined with multiple items* |
| **Statistical analysis** | |
| The outcome variable is categorized into three, but the researcher used multivariable linear regression. Why not multinomial logistic regression or ordinal logistic regression? | We appreciate the reviewer’s careful observation. We categorized the W-DEQ scores into three levels for descriptive purposes and used the continuous W-DEQ scores for the multivariable analysis and not the categorical variable. Multivariable linear regression analysis was therefore the most suitable as it maintains the full variability and statistical power of the continuous measure.  Particularly in a moderately small sample, categorizing the result for analysis—for example, by applying multinomial or ordinal logistic regression—can result in information loss and decreased power. This has been provided in the revised manuscript.  *For descriptive purposes, the primary outcome, W-DEQ scores, measured as a continuous variable, was categorized into three levels of FoC. However, a multivariable linear regression analysis was used to identify factors associated with FoC using continuous W-DEQ scores.* |
| Authors say they included “significant and clinically relevant variables,” but the criteria are not defined. | We appreciate the reviewer for this comment. We adjusted for both significant (p < 0.2) and clinically relevant variables, based on the existing literature and theoretical effects on FoC, in the multivariable linear regression models. We adopted this approach to ensure that both statistically significant and contextually important variables were considered in the adjusted models. This has been provided in the methods of the revised manuscript. Specifically under statistical analysis |
| It's unclear whether multicollinearity was checked. | We checked for multicollinearity using the Variance Inflation Factor. We have reported it under statistical analysis in the revised manuscript.  *The Variance Inflation Factor (VIF) was used to assess multicollinearity among independent variables for all the multivariable models (overall, nulliparous-specific and parous-specific). The mean VIF for the overall, nulliparous-specific and parous-specific models were 2.44 (range: 1.17 to 4.80), 2.94 (range: 1.48 to 6.86) and 3.02 (range: 1.10 to 6.97), respectively. All the mean VIF values were below the conventional threshold of 10, indicating no significant multicollinearity; however, some individual variables had higher VIFs. Specifically, in the nulliparous-specific model, tertiary education had a VIF of 6.86, and in the parous-specific model, occupation (formal: 6.97; informal: 6.08) had high VIFs. These variables were retained in the models due to their theoretical significance and because they had little to no effect on the estimates of other variables.* |
| Important variables like gestational age are missing from the model. | We thank the reviewer for this observation and we agree with the reviewer on this. It is possible that this variable could have impacted participants FoC. However, gestational age was not collected as a study variable. We have thus acknowledged it as a limitation of the study. |
| **W-DEQ scoring:** Severity levels are stated, but the manuscript uses both continuous and categorical outputs. The rationale for mixed reporting should be clarified | We appreciate the reviewer for this observation. Clarity has been provided in the revised manuscript.  *Severity categories were created and reported descriptively to provide clinically meaningful context to evaluate the distribution of FoC levels, instead of being used for modelling or sub-group comparison.* |
| **Results** |  |
| Response rate is not indicated in either the abstract or in the document. | We have reported it in the revised manuscript in the results of the main document.  *A total of 282 of the 290 women recruited participated in the study, yielding a response rate of 97.2%.* |
| In several places, interpretation borders on causal language despite the cross-sectional design. | We appreciate the reviewer’s comment on this. We have revised phrases like ‘independently associated with’, which could be interpreted as a causal link, to ‘significantly associated with’, which conveys statistical associations throughout the results. |
| What does the P-value in Table 1 and Table 2 indicate, and what is its importance? | We are grateful to the reviewer for this observation. The p-values in Tables 1 and 2 were used to determine whether participants’ characteristics and W-DEQ scores differed significantly between the two parity groups. |
| **Discussion** | |
| The discussion is long, sometimes repetitive, and occasionally drifts into general maternal-health topics not directly tied to the findings. | We are grateful to the reviewer for his/her careful review of the discussion. We have revised the discussion to avoid repetition and to avoid drifting into general maternal health topics. We ensured that all the discussions were tied to the study findings. |
| Some evidence is misinterpreted (e.g., the claim that parous women often report higher FoC contradicts the study’s results and global literature). | Again, we are grateful to the reviewer for his/her attention to detail. We have corrected the misinterpretation that parous women often report higher FoC as it was instead the opposite. The revised manuscript now interpret nulliparous women as having higher levels of FoC than parous women. |
| **Writing & Language** |  |
| There are recurring language and style issues: Long, multi-clause sentences reduce clarity. Occasional typos (e.g., “Wima” instead of “Wijma,” “Satasi” vs “Santasi” variation). Inconsistent tense and inconsistent formatting. Occasional grammar issues | Again, we are very grateful to the reviewer for his/her attention to detail. We have corrected all language and style issues. ‘Satasi’ has now been corrected to ‘Santasi’. |
