# Supplementary material for: Fear of childbirth among antenatal care attendees in a primary care setting in Kumasi, Ghana: Prevalence and parity-stratified determinants
Source: PLoS One. 2026 Sep 21;21(9):e0358457. doi: 10.1371/journal.pone.0358457 (PMC13592637; doi:10.1371/journal.pone.0358457)
Supplement: Supplementary file 7 [file pone.0358457.s006.docx]

**Manuscript number: PONE-D-25-51554R2**

**Manuscript Title:** Fear of childbirth among antenatal care attendees in a primary care setting in Kumasi, Ghana: Prevalence and parity-stratified determinants

**Recommendation:** Minor revision

The authors have thoroughly addressed the previous reviewers’ comments and successfully resolved the technical and methodological issues in the revised manuscript. However some minor typographical errors might have introduced or missed during the revision process and hence suggesting some minor corrections.

**Page 2, Line 34:** using hyphen (-) between identifying and parity-specific determinants “identifying-parity-specific determinants”

**Suggested correction:** identifying parity-specific determinants

**Line 34:** Misspelling of “Howerver”

**Page 3, Line 67:** worry, and psychological distress related to pregnancy, labor, and delivery.

**Suggested correction:** Please replace the word “delivery” with “childbirth” to more accurately reflect the clinical context discussed in this section.

**Page 6, Line 170:** spacing between the word “analyzedand”
