# Supplementary material for: Fear of childbirth among antenatal care attendees in a primary care setting in Kumasi, Ghana: Prevalence and parity-stratified determinants
Source: PLoS One. 2026 Sep 21;21(9):e0358457. doi: 10.1371/journal.pone.0358457 (PMC13592637; doi:10.1371/journal.pone.0358457)
Supplement: Supplementary file 8 [file pone.0358457.s007.docx]

**AUTHORS' RESPONSES TO REVIEWER’S COMMENTS**

| **Reviewer’s comments** | **Authors’ response** |
| --- | --- |
| **Reviewer 2** | |
| The title of study is not suitable with the substances of the manuscript | We are grateful to the reviewer for this thoughtful comment. The title of the study has been revised to read as;  *Fear of childbirth among antenatal care attendees in a primary care setting in Kumasi, Ghana: Prevalence and parity-stratified determinants* |
| The novelty of study need to be explained detail | We are grateful to the reviewer for drawing our attention to this. The novelty of the study has been explained in detail in the revised manuscript. It now reads as;  *Notably, considering parity only as an adjustment variable may mask unique obstetric and psychosocial routes by which FoC emerges in first-time mothers compared with women with previous birth experience. This is because the factors contributing to FoC may differ across these parity groups, potentially limiting a more nuanced understanding of risk profiles and hindering the design of patient-centered antenatal counselling strategies. To the best of the authors’ knowledge, no study in Ghana has identified parity-stratified risk factors among nulliparous and parous women. This methodological approach provides an opportunity for identifying within-group risk factors to inform routine antenatal care (ANC) screening in resource-limited primary care settings.* |
| Keywords: please add them from a term mesh, put them, and locate them alphabetically. | We are grateful to the reviewer for this comment and in line with that the keywords have been revised as follows;  *Keywords: Antenatal care; Fear; Ghana; Parity; Pregnancy; Risk factors* |
| The study context is not well described, and the gap or state of the art also is not clear as well as the method | We are grateful to the reviewer for this observation. The introduction has been revised to clearly describe the study context, existing knowledge gap, and methodological rationale. Specifically, we indicated that no study in Ghana has identified parity-stratified risk factors among nulliparous and parous women, providing insight into parity-specific risk profiles, which can guide patient-centered antenatal intervention in low-resource settings |
| The suggestion is that it is better if method of this study is in qualitative design | We are grateful to the reviewer for this comment. While qualitative design could have offered the opportunity to explore their subjective experiences of childbirth fear in detail, our aim was to quantify the prevalence and also identify parity-specific determinants among antenatal care attendees in a primary care setting. Given this, a cross-sectional design was therefore more appropriate. Future studies could complement our findings using a qualitative study approach. We have included it as a study recommendation in the revised manuscript.  *Future research should explore these strategies using longitudinal, interventional, and qualitative designs to guide policy and practice in Ghana and similar settings.* |
| The sample size calculation is unclear. What are the criteria for inclusion and exclusion in the sample study? How the researchers recruit the respondents? | We are grateful to the reviewer for these observations. In response, we have revised the sample size calculation to improve clarity in the manuscript. Statements on the inclusion and exclusion criteria have been added under the subheading “Study design, setting, and population.” Additionally, we created a separate subheading, “Participants’ recruitment,” to clearly describe the recruitment process, rather than including it within the “Sample size estimation and sampling technique” section as in the original submission. The recruitment procedure has been elaborated to ensure transparency and reproducibility. |
| It is unusual to find p-values in tables 1 and 2, especially on the characteristic data | We appreciate the reviewer's comment. We understand that it is unusual to find p-values in Tables 1 and 2, especially on the characteristics data, we prefer to maintain it in its original form with the p-values. This is because the p-values provide useful insights into statistically significant differences between nulliparous and parous women, which are directly relevant to our study’s objectives. For example, they highlight where socio-demographic or obstetric characteristics differ by parity, helping to contextualize subsequent analyses and interpretation of fear of childbirth (FoC) outcomes.  As illustrated in our discussion, one notable finding among nulliparous women was the association between late ANC booking and increased FoC. Late ANC booking may limit access to early interventions, including education and psychosocial support, that can reduce fear. Evidence suggests that nulliparous women who engage in timely ANC are better prepared for childbirth, which subsequently helps to reduce FoC (41). Retaining p-values in these tables allows readers to see the statistical basis for such parity-specific observations and supports the narrative in the discussion section |
| The research relates the findings to previous work is so simple, need more references as well as their implications for practice need to be explore more | We are grateful to the reviewer for this observation. We have provided one or two additional references in the discussion and also improved the study's implications for practice. Specifically, we have included the statement below in the section on “implications for practice and research.”  *Our findings suggest that, in addition to routine ANC programs that screen for FoC, they should also integrate patient-centered strategies that account for the unique risk profiles within each parity group. There is a need for healthcare managers to train professionals to identify at-risk populations, provide patient-centered education, and involve family members when necessary to promote social support. Incorporating FoC screening tools into routine ANC guidelines could help identify vulnerable women who could benefit from additional psychological or social support strategies, thereby enhancing maternal satisfaction and childbirth preparedness.* |
| Need more explain significance of results, and how they contribute to the overall state of knowledge or how they advance knowledge as well | We appreciate the reviewer for these comments. We have revised the manuscript to reflect this.  *Another critical factor was the inverse association between family support and FoC, underscoring the broader literature emphasizing the protective role of social support on maternal anxiety and childbirth-related stress (50,51). Women with strong support systems tend to feel more empowered, informed, and emotionally secure, which often results in reduced levels of fear and anxiety (52). This finding underpins the importance of social networks in improving childbirth experiences in Ghana and advances knowledge of the psychosocial factors that influence FoC in a limited-resource setting. The protective role of family support increases the understanding of how social variables shape FoC. This highlights the importance of integrating family-oriented approaches into ANC services to reduce FoC and improve maternal well-being. This finding is particularly relevant in the Ghanaian context, where extended family structures and community networks play a crucial role in shaping women’s health experiences. Encouraging family involvement in ANC and advocating for supportive environments may be essential to reducing FoC.* |
| **Reviewer 3** | |
| The study addresses a relevant research question within the scope of PLOS ONE and presents data of potential interest. However, several issues related to methodological clarity, data analysis, reporting, and language require attention to ensure transparency, reproducibility, and adherence to PLOS ONE standards. | We are very grateful to the reviewer for investing his/her time to review this work. It is worth noting that the reviewer has provided insightful comments that will enhance the quality of the work.  We have addressed all issues raised by the reviewer in the revised manuscript.  We have provided a point-by-point response to each issue raised by the reviewer below. |
| **TITLE** | |
| *line 1: Fear of childbirth in Ghana: Prevalence and within parity-specific determinants among antenatal care attendees in a health facility in Kumasi, Ghana (“within parity-specific determinants” is grammatically awkward).* ***Suggested correction:***  *Fear of childbirth in Ghana: Prevalence and parity-specific determinants among antenatal care attendees in a health facility in Kumasi* | We are grateful to the reviewer for this observation. The title has been revised to remove redundancy and improve clarity. It now reads:  *Fear of childbirth among antenatal care attendees in a primary care setting in Kumasi, Ghana: Prevalence and parity-stratified determinants* |
| **ABSTRACT** | |
| *Line 34: Previous studies on Fear of Childbirth (FoC) across sub-Saharan Africa have typically reported prevalence separately by parity or treated it as a covariate in multivariable analyses, with little attention given to identifying within-parity-specific determinants (“within-parity-specific” is repetitive and awkward)* ***Suggested correction:***  *with little attention given to identifying parity-specific determinants.* | We are grateful to the reviewer for this observation. This phrase has been corrected in the revised manuscript |
| *line 34-35 : Yet, first-time mothers and those with previous birth experiences may require different forms of antenatal support (“Yet,” at the beginning is acceptable but slightly informal).* ***Suggested correction:***  *However, first-time mothers and those with previous birth experiences may require different forms of antenatal support* | Following the reviewer’s suggestion, “Yet” has been replaced with “However” to improve academic tone. |
| *line 43 : Stratified multivariable linear regression models were applied separately to identify parity-specific determinants among the participants(Clear, but “applied separately” is redundant)* ***Suggested correction:***  *Stratified multivariable linear regression models were applied to identify parity-specific determinants.* | The phrase has been revised. The sentence now reads:  *“Stratified multivariable linear regression models were applied to identify parity-specific determinants.”* |
| *line 51: independently associated with FOC (Inconsistent capitalization (FoC vs FOC).* ***Suggested correction:***  *independently associated with FoC. (Apply this correction* ***throughout the manuscript****.)* | Capitalization has been standardized to “FoC” throughout the manuscript |
| ***Conclusion***  *differed by parity and within parity-specific factors.*  ***Comment:*** *line 57 : Redundant and grammatically awkward.*  ***Suggested correction:***  *differed by parity and across parity-specific factors.* | This has been revised to “across parity-specific factors” to improve grammatical accuracy. |
| **INTRODUCTION** | |
| *line 67 : worry and psychological distress related to pregnancy, labour and delivery(comma consistency)*  ***Suggested correction:***  *worry, and psychological distress related to pregnancy, labour, and delivery.* | The sentence has been revised to ensure consistent punctuation: “worry, and psychological distress related to pregnancy, labour, and delivery.” |
| *Line 68 somatizations (British spelling is acceptable, but be consistent).*  ***Suggested correction (optional):***  *somatization* | Spelling has been standardized to “somatization” throughout the manuscript. |
| *line 76 : An interplay of sociodemographic, psychosocial and obstetric factors( Missing comma).* ***Suggested correction:***  *sociodemographic, psychosocial, and obstetric factors* | The sentence has been revised to read: “sociodemographic, psychosocial, and obstetric factors.” |
| *Line 88-89 : few of have directly assessed the parity-specific determinants(Grammatical error or missing word).* ***Suggested correction:***  *few have directly assessed parity-specific determinants* | This has been corrected to “few have directly assessed parity-specific determinants in the region |
| **METHODS** | |
| *Line 113 : Pregnant women who looked critically ill(“looked” is subjective and informal).* ***Suggested correction:***  *Pregnant women who were critically ill* | We appreciate the reviewer's attention to detail. The phrase has been revised to “were critically ill” to ensure objective and professional language. |
| *By adjusting for a contingency rate of 10%*  ***Comment:*** *line 120 : “contingency rate” is unclear.*  ***Suggested correction:***  *By adjusting for a 10% non-response rate* | We are grateful for this comment. This has been revised to “10% non-response rate |
| *records units line 125: records units (Plural unnecessary).*  ***Suggested correction:***  *records unit* | This has been corrected to “records unit.” |
| *Line 159 : The data collected was analyzed(Subject–verb agreement.)* ***Suggested correction:***  *The data collected were analyzed* | We are grateful to the reviewer. The sentence has been revised to reflect the reviewer’s recommendation. |
| *Line 147 : the decision of ANC booking is influenced by who(Sentence fragment / unclear phrasing.)* ***Suggested correction:***  *whether the decision regarding ANC booking was influenced by another person* | We thank the reviewer for these comments. The sentence has been revised to clarify whether the decision regarding ANC booking was influenced by another person. It now reads as;  *Additionally, the clinical factors included ANC booking, whether the decision regarding ANC booking was influenced by another person....* |
| ***W-DEQ description***  *Line 150-151: 33- item scale(Spacing error)*  ***Suggested correction:***  *33-item scale* | This has been corrected to “33-item scale.” |
| **STATISTICAL ANALYSIS** | |
| (Data analysis): Subject–verb agreement error (“data was”). | We appreciate the reviewer’s careful observation. This has been corrected to “The data collected were analyzed.” |
| *Line 165-166 : Parity-stratified comparison between demographic and clinical characteristics(“comparison” should be plural or rephrased)* ***Suggested correction:***  *Parity-stratified comparisons of demographic and clinical characteristics* | We are grateful to the reviewer for his/her attention to detail. This correction has been effected in the revised manuscript. |
| *Line 171 : factors that each independently associated(Missing verb).*  ***Suggested correction:***  *factors that were independently associated* | We appreciate the reviewer's comment. This has been corrected to “factors that were independently associated.” In the revised manuscript |
| **RESULTS** | |
| *Line 190-191 : parous women (23.9 ± 3.9 years) being significantly older than nulliparous women (30.2 ± 5.0 years) ; (Values are reversed (logical error).* ***Suggested correction:***  *nulliparous women (23.9 ± 3.9 years) being significantly younger than parous women (30.2 ± 5.0 years)* | We thank the reviewer for identifying this error. The values have been corrected, with nulliparous women now accurately reported as younger than parous women. |
| *ANC booking (weeks) Early (1 – 12 (Missing closing parenthesis.* ***Suggested correction:***  *Early (1–12)*  *38.70 (Inconsistent decimal places and missing parenthesis).*  ***Suggested correction:***  *38.7)* | We are grateful to the reviewer for this observation. The values in the table have been revised to correct missing parentheses and standardize decimal formatting. |
| **Discussion** |  |
| *Line 277 : skewedness in the distribution(Nonstandard word)* ***Suggested correction:***  *skewness in the distribution* | This has been corrected to the standard statistical term “skewness.” |
| *Line 292-293 : Compared to nulliparous women, parous women often report higher levels of FoC(This contradicts earlier statements)* ***Suggested correction:***  *Compared to parous women, nulliparous women often report higher levels of FoC* | We are grateful to the reviewer for his/her attention to detail. We have corrected the misinterpretation that parous women often report higher FoC, as it was, instead, the opposite. The revised manuscript now interprets nulliparous women as having higher levels of FoC than parous women. |
| *Line 332 : prevalence of fear FOC(Typographical error) .****Suggested correction:***  *prevalence of FoC* | This has been corrected to “FoC.” |
| *Line 377 : Routine screening for FOC(Capitalization inconsistency)* ***Suggested correction:***  *Routine screening for FoC* | Capitalization has been standardized to “FoC” throughout the manuscript |
| **ACKNOWLEDGEMENT** | |
| *Line 384 : Medical Center's management(Spelling consistency with earlier “Centre”)*  ***Suggested correction:***  *Medical Centre’s management* | Spelling has been standardized to “Medical Centre’s management,” consistent with earlier usage. |
