# Supplementary material for: Fear of childbirth among antenatal care attendees in a primary care setting in Kumasi, Ghana: Prevalence and parity-stratified determinants
Source: PLoS One. 2026 Sep 21;21(9):e0358457. doi: 10.1371/journal.pone.0358457 (PMC13592637; doi:10.1371/journal.pone.0358457)
Supplement: Supplementary file 9 [file pone.0358457.s008.docx]

**Manuscript number: PONE-D-25-51554R2**

**Manuscript Title:** Fear of childbirth among antenatal care attendees in a primary care setting in Kumasi, Ghana: Prevalence and parity-stratified determinants

**Recommendation:** Accept

I would like to thank the authors for their constructive response and the effort they have put in to revise the manuscript.

The authors have addressed all the concerns raised in the review and also have made additional refinements on several sections of the text which have made the manuscript much better and more intelligible.

I feel that the manuscript now meets the standards of the journal and hence, recommend it for acceptance.
