# Supplementary material for: Fear of childbirth among antenatal care attendees in a primary care setting in Kumasi, Ghana: Prevalence and parity-stratified determinants
Source: PLoS One. 2026 Sep 21;21(9):e0358457. doi: 10.1371/journal.pone.0358457 (PMC13592637; doi:10.1371/journal.pone.0358457)
Supplement: Supplementary file 10 [file pone.0358457.s009.docx]

PLOS One

Fear of childbirth among antenatal care attendees in a primary care setting in Kumasi,

Ghana: Prevalence and parity-stratified determinants

--Manuscript Draft--

| Manuscript Number: | PONE-D-25-51554R3 |
| --- | --- |
| Article Type: | Research Article |
| Full Title: | Fear of childbirth among antenatal care attendees in a primary care setting in Kumasi, Ghana: Prevalence and parity-stratified determinants |
| Short Title: | Parity-stratified determinants of fear of childbirth |
| Corresponding Author: | Douglas Aninng Opoku, MPH  Kwame Nkrumah University of Science and Technology College of Health Sciences Kumasi, Ashanti Region GHANA |
| Keywords: | Antenatal Care; Fear; Ghana; parity; pregnancy; Risk Factors |
| Abstract: | Background: Previous studies on Fear of Childbirth (FoC) across sub-Saharan Africa have typically reported prevalence separately by parity or treated it as a covariate in multivariable analyses, with little attention given to identifying parity-specific determinants. However, first-time mothers and those with previous birth experiences may require different forms of antenatal support. To address this gap, we assessed the prevalence of FoC and identified parity-specific determinants among antenatal care attendees in Ghana to inform targeted interventions.  Methods: A facility-based cross-sectional study was carried out among pregnant women attending antenatal care at a primary healthcare facility in Kumasi. Data were collected using an interviewer-administered questionnaire. The primary outcome of the  study, FoC, was assessed using the English version of the Wijma Delivery Expectation/Experience Questionnaire (W-DEQ). Additional data included sociodemographic and obstetric characteristics. Stratified multivariable linear regression models were applied to identify parity-specific determinants among the participants.  Results: Of the 282 women who participated in the study, 22% had high-to-severe FoC. Nulliparous women (median: 46.0, IQR: 37.0–66.0) reported significantly higher median W-DEQ scores than parous women (median: 39.0, IQR: 32.0–58.5). In a paritystratified analysis, among nulliparous women, factors such as a history of family abuse (adj. β: 16.32, 95%CI: 2.50, 30.14) and late ANC booking (adj. β: 13.13, 95%CI: 1.43, 24.84) were significantly associated with FoC. Among parous women, factors such as secondary education (adj. β: -7.63, 95%CI: -14.88, -0.37), intimate partner violence (adj. β: 10.87, 95%CI: 0.69, 21.04), unplanned pregnancy (adj. β: 8.38, 95%CI: 2.51, 14.26), and strong family support (adj. β: -27.94, 95%CI: -38.77, -17.11) were significantly associated with FoC.  Conclusion: FoC is common among this cohort of pregnant women, and its determinants differed by parity and across parity-specific factors. The parity-stratified determinants underscore the need for parity-tailored psychosocial care at ANC, incorporating targeted screening and support procedures aligned with parityassociated risk pathways to enhance maternal well-being. |
| Order of Authors: | Juliana Ofosua Addo, MPH |
|  | Douglas Aninng Opoku, MPH |
|  | Julius Kwabena Karikari, Mphil |
|  | Emmanuel Konadu, Mphil |
|  | Mercy Addae, Mphil |
|  | Francis Kwabena Kyei, MPH |
|  | Mawuse Kanfra, FGCP |
|  | Obed Kwabena Offe Amponsah, PhD |
|  | Eric Adjei Boadu, PhD |

| Response to Reviewers: | Comment: The authors have thoroughly addressed the previous reviewers’ comments and successfully resolved the technical and methodological issues in the revised manuscript. However some minor typographical errors might have introduced or missed during the revision process and hence suggesting some minor corrections.  Response: We are grateful to you, the other reviewer and the editor for ensuring that the quality and rigour of our manuscript is improved. We strongly believe that your comments have been extremely helpful in improving the quality of the manuscript, and we appreciate them.  Comment: Page 2, Line 34: using hyphen (-) between identifying and parity-specific determinants “identifying-parity-specific determinants”  Suggested correction: identifying parity-specific determinants  Response: We are grateful to the reviewer for drawing our attention to this. We statement in reference has been revised and it now reads as;  Previous studies on Fear of Childbirth (FoC) across sub-Saharan Africa have typically reported prevalence separately by parity or treated it as a covariate in multivariable analyses, with little attention given to identifying parity-specific determinants  Comment: Line 34: Misspelling of “Howerver”  Response: This has been corrected in the revised manuscript, and it now reads as;  However, first-time mothers and those with previous birth experiences may require different forms of antenatal support  Comment: Page 3, Line 67: worry, and psychological distress related to pregnancy, labor, and delivery.  Suggested correction: Please replace the word “delivery” with “childbirth” to more accurately reflect the clinical context discussed in this section.  Response: We are grateful to the reviewer for this comment, and in line with that, the statement in reference has been revised to read as;  It involves persistent feelings of anxiety, worry, and psychological distress related to pregnancy, labour, and childbirth  Comment: Page 6, Line 170: spacing between the word “analyzedand”  Response: This has been done as recommended. It now reads as;  The data collected were analysed and used to amend the questionnaire before it was used in the final study. |
| --- | --- |
| Additional Information: |  |
| Question | Response |
| Financial Disclosure  Enter a financial disclosure statement that describes the sources of funding for the work included in this submission. Review the [submission guidelines](http://journals.plos.org/plosone/s/submission-guidelines#loc-financial-disclosure-statement) for detailed requirements. View published research articles from [PLOS ONE](https://journals.plos.org/plosone/) for specific examples.  This statement is required for submission | The author(s) received no specific funding for this work. |

| and will appear in the published article if the submission is accepted. Please make sure it is accurate. |  |
| --- | --- |
| Funded studies  Enter a statement with the following details:   - Initials of the authors who received each award - Grant numbers awarded to each author - The full name of each funder - URL of each funder website - Did the sponsors or funders play any role in the study design, data collection and analysis, decision to publish, or preparation of the manuscript? |  |
| Did you receive funding for this work? |  |
| Competing Interests  Use the instructions below to enter a competing interest statement for this submission. On behalf of all authors, disclose any [competing interests](http://journals.plos.org/plosone/s/competing-interests) that could be perceived to bias this work—acknowledging all financial support and any other relevant financial or nonfinancial competing interests.  This statement is required for submission and will appear in the published article if the submission is accepted. Please make sure it is accurate and that any funding sources listed in your Funding Information later in the submission form are also declared in your Financial Disclosure statement.  View published research articles from [PLOS ONE](http://journals.plos.org/plosone/) for specific examples. | The authors have declared that no competing interests exist. |

| NO authors have competing interests  Enter: The authors have declared that no competing interests exist.  Authors with competing interests  Enter competing interest details beginning with this statement:  I have read the journal's policy and the authors of this manuscript have the following competing interests: [insert competing interests here] |  |
| --- | --- |
| * typeset |  |
| Data Availability  Authors are required to make all data underlying the findings described fully available, without restriction, and from the time of publication. PLOS allows rare exceptions to address legal and ethical concerns. See the [PLOS Data Policy](http://journals.plos.org/plosone/s/data-availability) and [FAQ](http://journals.plos.org/plosone/s/data-availability#loc-faqs-for-data-policy) for detailed information.  A Data Availability Statement describing where the data can be found is required at submission. Your answers to this question constitute the Data Availability Statement and will be published in the article, if accepted. | Yes - all data are fully available without restriction |
| Important: Stating ‘data available on request from the author’ is not sufficient. If your data are only available upon request, select ‘No’ for the first question and explain your exceptional situation in the text box. |  |
| Do the authors confirm that all data underlying the findings described in their manuscript are fully available without restriction? |  |
| Additional data availability information: |  |
| Ethics Statement  Enter an ethics statement for this submission. This statement is required if | This study was conducted with strict adherence to the ethical principles outlined in the  Declaration of Helsinki. The Committee on Human Research, Publications, and Ethics (CHRPE), School of Medicine and Dentistry, Kwame Nkrumah University of Science and Technology (KNUST), Kumasi (CHRPE/AP/025/25) granted ethical approval for |

| the study involved:   - Human participants - Human specimens or tissue - Vertebrate animals or cephalopods - Vertebrate embryos or tissues - Field research   Write "N/A" if the submission does not require an ethics statement.  General guidance is provided below. Consult the [submission guidelines](http://journals.plos.org/plosone/s/submission-guidelines#loc-guidelines-for-specific-study-types) for detailed instructions. Make sure that all information entered here is included in the Methods section of the manuscript. | this study. Written approval was sought from the management of the study site. Before recruitment, all study participants went through a mandatory informed consent process and signed an informed consent. As part of the consent process, all the study participants were provided with information about the objective of the study, its significance, and the possible risks of involvement. |
| --- | --- |

| Format for specific study types  Human Subject Research (involving human participants and/or tissue)   - Give the name of the institutional review board or ethics committee that approved the study - Include the approval number and/or a statement indicating approval of this research - Indicate the form of consent obtained (written/oral) or the reason that consent was not obtained (e.g. the data were analyzed anonymously)   Animal Research (involving vertebrate animals, embryos or tissues)   - Provide the name of the Institutional Animal Care and Use Committee (IACUC) or other relevant ethics board that reviewed the study protocol, and indicate whether they approved this research or granted a formal waiver of ethical approval - Include an approval number if one was obtained - If the study involved non-human primates, add additional details about animal welfare and steps taken to ameliorate suffering - If anesthesia, euthanasia, or any kind of animal sacrifice is part of the study, include briefly which substances and/or methods were applied   Field Research  Include the following details if this study involves the collection of plant, animal, or other materials from a natural setting:   - Field permit number - Name of the institution or relevant body that granted permission |  |
| --- | --- |
|  |  |
| <strong>Data Availability</strong><br> <br>Before publication, Authors are required to make fully available and without restriction all data underlying their findings. Sharing the data underlying your findings increases the visibility, credibility, and impact of your work, while supporting transparency and future scientific discovery. Please see our <a  href="https://journals.plos.org/plosone/s/d ata-availability" target="_blank">PLOS Data Policy page</a> for detailed information on this policy.</p> <br>A <strong>Data Availability | All relevant data are within the manuscript and its Supporting Information files |

| Statement</strong>, detailing where the data can be accessed, is required at first submission. Insert your Data Availability  Statement in the box below.</p>  <br>Please see the <a  href="https://journals.plos.org/plosone/s/s ubmission-guidelines#loc-data-reporting" target="_blank">data reporting</a> section of our submission guidelines for instructions on what you need to include in your Data Availability Statement. Examples of acceptable Data Availability  Statements are included here for reference:</p>  <ol>  <li>The minimal data set [and accompanying code, where generated] is available at [repository name] via [DOI hyperlink]</li>  <li>No data was generated by this study. The following existing data sources were used: [dataset name] from [dataset location] available via [web address or  DOI hyperlink].</li>  <li>For Study Protocols: No datasets were generated or analysed during the current study. All relevant data from this study will be made available upon study completion.</li>  </ol>  <br><i>PLOS allows rare exemptions to address legal and ethical concerns. If you have legal or ethical restrictions, please use the box below to detail these in full sentences for the Journal team to consider.</i></p>  <br>This statement will be typeset if the manuscript is accepted for publication.</p> |  |
| --- | --- |

Manuscript	[Click here to access/download;Manuscript;REVISED](https://www2.cloud.editorialmanager.com/pone/download.aspx?id=44408700&guid=610d5d61-0744-4187-a047-7465210518f7&scheme=1)

[MANUSCRIPT_ CLEAN COPY.docx](https://www2.cloud.editorialmanager.com/pone/download.aspx?id=44408700&guid=610d5d61-0744-4187-a047-7465210518f7&scheme=1)

# 1 Fear of childbirth among antenatal care attendees in a primary care setting in Kumasi, 2 Ghana: Prevalence and parity-stratified determinants

3

4 **Authors;** Juliana Ofosua Addo*^1,2^, Douglas Aninng Opoku*^3,4^, Julius Kwabena Karikari^3^, 5 Emmanuel Konadu^3^, Mercy Addae^3,4^, Francis Kwabena Kyei^1^, Mawuse Kanfra^5^, Obed 6 Kwabena Offe Amponsah^6^, & Eric Adjei Boadu^1^

7

# 8 Affiliations;

9 ^1^Department of Population, Family and Reproductive Health, School of Public Health, Kwame
10 Nkrumah University of Science and Technology, Kumasi, Ghana
11 ^2^HopeXchange Medical Centre, Kumasi, Ghana
12 ^3^University Hospital, Kwame Nkrumah University of Science and Technology, Kumasi, Ghana
13 ^4^Department of Epidemiology and Biostatistics, School of Public Health, Kwame Nkrumah
14 University of Science and Technology, Kumasi, Ghana
15 ^5^St. Dominic Catholic Hospital, Akwatia, Ghana
16 ^6^Department of Pharmacy Practice, Faculty of Pharmacy and Pharmaceutical Sciences, Kwame 17 Nkrumah University of Science and Technology, Kumasi, Ghana

## 18

19 *Corresponding author: Douglas Aninng Opoku: douglasopokuaninng@gmail.com; Juliana 20 Ofosuaa Addo: julianaaddo@gmail.com

21

## 22 23

24	25	26	27	28	29	30

# Abstract

**Background:** Previous studies on Fear of Childbirth (FoC) across sub-Saharan Africa have typically reported prevalence separately by parity or treated it as a covariate in multivariable analyses, with little attention given to identifying parity-specific determinants. However, firsttime mothers and those with previous birth experiences may require different forms of antenatal support. To address this gap, we assessed the prevalence of FoC and identified parity-specific determinants among antenatal care attendees in Ghana to inform targeted interventions.

**Methods:** A facility-based cross-sectional study was carried out among pregnant women attending antenatal care at a primary healthcare facility in Kumasi. Data were collected using an interviewer-administered questionnaire. The primary outcome of the study, FoC, was assessed using the English version of the Wijma Delivery Expectation/Experience Questionnaire (W-DEQ). Additional data included sociodemographic and obstetric characteristics. Stratified multivariable linear regression models were applied to identify parity-specific determinants among the participants.

**Results:** Of the 282 women who participated in the study, 22% had high-to-severe FoC. Nulliparous women (median: 46.0, IQR: 37.0–66.0) reported significantly higher median WDEQ scores than parous women (median: 39.0, IQR: 32.0–58.5). In a parity-stratified analysis, among nulliparous women, factors such as a history of family abuse (adj. β: 16.32, 95%CI: 2.50, 30.14) and late ANC booking (adj. β: 13.13, 95%CI: 1.43, 24.84) were significantly associated with FoC. Among parous women, factors such as secondary education (adj. β: -7.63, 95%CI: -14.88, -0.37), intimate partner violence (adj. β: 10.87, 95%CI: 0.69, 21.04), unplanned pregnancy (adj. β: 8.38, 95%CI: 2.51, 14.26), and strong family support (adj. β: -27.94, 95%CI: -38.77, -17.11) were significantly associated with FoC.

**Conclusion:** FoC is common among this cohort of pregnant women, and its determinants differed by parity and across parity-specific factors. The parity-stratified determinants underscore the need for parity-tailored psychosocial care at ANC, incorporating targeted screening and support procedures aligned with parity-associated risk pathways to enhance maternal well-being.

Keywords: Antenatal care; Fear; Ghana; Parity; Pregnancy; Risk factors

# Introduction

Fear of childbirth (FoC) is a well-documented psychological phenomenon affecting both maternal and infant health outcomes worldwide (1). It involves persistent feelings of anxiety, worry, and psychological distress related to pregnancy, labour, and childbirth. It can manifest through emotional symptoms such as mood swings, somatisation, and cognitive effects like impaired decision-making (2–4). FoC is associated with adverse consequences, including increased risk of maternal depressive symptoms, avoidance behaviours such as preference for caesarean section without medical indication, and undesirable birth outcomes (5–9). A systematic review estimated the prevalence of FoC ranges between 4% and 43%, with an overall pooled prevalence of 14% worldwide (4). Among pregnant women, FoC is influenced by an interplay of sociodemographic, psychosocial, and obstetric factors, including younger age, lower educational attainment, nulliparity, previous obstetric complications, and lack of social support (6,9,10). While nulliparous women often report higher FoC due to inexperience and uncertainty, parous women may develop fears related to adverse past birth experiences (4,6).

Fear of childbirth remains a significant antenatal concern in sub-Saharan Africa, with prevalence studies from nearby countries reporting similar rates (9,11,12). However, direct prevalence and determinants data for Ghana are limited, and existing evidence suggests that cultural and mental health factors – such as anxiety and depression – and obstetric history significantly shape women’s childbirth experiences (11). Furthermore, the specific factors associated with FoC within parity groups remain underexplored. Although previous studies have highlighted the role of parity in shaping FoC (10,12), few have directly assessed parityspecific determinants in the region. Most of these studies either report prevalence separately or treat parity merely as a covariate in multivariable analyses (9,12–14), potentially masking unique risk profiles.

Notably, considering parity only as an adjustment variable may mask unique obstetric and psychosocial routes by which FoC emerges in first-time mothers compared with women with previous birth experience. This is because the factors contributing to FoC may differ across these parity groups, potentially limiting a more nuanced understanding of risk profiles and hindering the design of patient-centred antenatal counselling strategies. To the best of the authors’ knowledge, no study in Ghana has identified parity-stratified risk factors among nulliparous and parous women. This methodological approach provides an opportunity for identifying within-group risk factors to inform routine antenatal care (ANC) screening in resource-limited primary care settings.

Therefore, a stratified approach is essential to determine whether socio-demographic, obstetric, and psychosocial factors differ by parity, as first-time mothers and those with previous birth experiences may require different antenatal support or interventions. To address these gaps, we aimed to assess the prevalence of FoC and identify parity-specific determinants among ANC attendees in a primary healthcare facility in Kumasi, Ghana. Identifying parity-specific evidence provides guidance for targeted psychosocial care and structured screening within routine ANC to reduce the magnitude of FoC and improve maternal well-being.

**Methods**

# Study design, setting, and population

A facility-based cross-sectional study was conducted among pregnant women attending ANC at HopeXchange Medical Centre in Santasi, Kumasi, between 20^th^ January 2025 and 12^th^ March 2025. Santasi is a suburban area of Kumasi, the capital of the Ashanti Region in Ghana. HopeXchange Medical Centre is a faith-based facility that provides comprehensive general and specialised medical services. The ANC clinic operates Monday to Friday and is staffed by two doctors and ten midwives. Upon arrival, pregnant women present their ANC booklets and hospital cards at the records unit, after which they are assigned to a midwife for routine consultations. Physician consultations are provided as needed. The facility serves an antenatal population of approximately 5000 women and recorded over 600 deliveries in 2024.

The study population comprised all pregnant women who received ANC at the clinic during the study period. The inclusion criteria were pregnant women of any gestational age who were willing to provide informed consent. The exclusion criteria were pregnant women who were critically ill and required hospitalisation at the time of data collection.

The study included all pregnant women irrespective of parity. The study aimed to identify factors associated with FoC in the overall population and to conduct a parity-stratified analysis. This approach enables the identification of both general and parity-specific risk factors to guide targeted ANC counselling and interventions.

# Sample size estimation

The sample size was estimated using the single population proportion formula (13), (𝑠 = (𝑍^2 (𝑃)(1 − 𝑃))/𝐸^2). Where *Z* represents the standard normal value at a 95% confidence interval (1.96), *E* is the error margin (0.05), and *P* is the estimated proportion of pregnant women with FoC (22%) from a previous study (14). This yielded an initial sample size of 264. After adjusting for a 10% non-response rate, the final required sample size was 290 pregnant women.

# Recruitment procedure

Participants were recruited using a simple random sampling technique from a daily attendance list of all pregnant women who had registered for ANC by 10:00 am at the records unit. This time cut-off was operationally realistic, as a feasibility assessment conducted prior to data collection indicated that over 90% of attendees had registered by then. Unique identifiers were assigned, and participants were randomly selected using a lottery method without replacement. Selected pregnant women were contacted individually by research staff to determine their eligibility for inclusion in the study. This process was repeated daily until the estimated sample size was achieved (over 24 days, averaging 12 participants per day).

# Data collection tools and procedures

Data collection was conducted by five research assistants, comprising three midwives and two physician assistants. Prior to data collection, the research assistants participated in a two-day training workshop facilitated by a biostatistician and the principal investigator. The training covered key areas, including quantitative data collection, the consent process, and the administration of the data collection tool.

An interviewer-administered questionnaire was used to collect data for the study. The investigators developed the questionnaire after reviewing relevant literature (15–21). It was structured into three parts: 1) demographic characteristics, 2) clinical factors, and 3) FoC. The demographic variables included age, marital status, educational level, occupation, religion, head of household, perceived family support, experiencing intimate partner violence (IPV), and history of abuse in the family. These three psychosocial variables were measured using singleitem self-report questions to reduce respondents’ burden and enhance clarity, given their sensitive nature in an ANC setting. Additionally, the clinical factors included ANC booking, whether the decision regarding ANC booking was influenced by another person, whether the current pregnancy was planned, parity, and history of abortion.

FoC, the primary outcome of the study, was measured with the English version of the Wijma Delivery Expectation/Experience Questionnaire (W-DEQ) (22). The W-DEQ is a validated 33item scale designed to measure women’s experiences or expectations of childbirth (22). Each item is rated on a 5-point Likert scale with minimum and maximum scores of 0 and 165, respectively. Although the W-DEQ is a continuous scale, prevalence was defined using a literature-based cut-off for the overall scores: low (≤ 37), moderate (38 – 65), high (66 – 84), or severe (≥ 85) levels of FoC (22). Participants who scored high or severe categories (≥66) were classified as FoC cases (23–25). The W-DEQ has been widely used in other settings to measure FoC among pregnant women (11,20,26,27). In the current study, the scale demonstrated high internal consistency (Cronbach's alpha = 0.975).

The questionnaire was pretested with 20 pregnant women at a similar health facility in another town. The data collected were analysed and used to amend the questionnaire before it was used in the final study. Specifically, the amendment affected the order of the questions, the rewording of ambiguous items, and the inclusion of a preamble to ensure participants understood the questions.

# Statistical analysis and data quality management

Data were exported to Stata version 16 for quality checks, coding, and statistical analysis. Descriptive statistics, such as frequencies and percentages, were used to describe categorical variables, while means with standard deviations and medians with interquartile ranges were used to describe continuous data, where applicable. Parity-stratified comparisons between demographic and clinical characteristics were examined using the chi-square test and independent sample t-test, where necessary. For descriptive purposes, the W-DEQ score, measured as a continuous variable, was categorised into three FoC levels. Severity categories were created and reported descriptively to provide a clinically meaningful context to evaluate the distribution of FoC levels, instead of being used for modelling or subgroup comparison.

However, a multivariable linear regression analysis was used to identify factors associated with FoC using continuous W-DEQ scores. We adjusted for both significant (p < 0.2) and clinically relevant variables, based on the existing literature and theoretical effects on FoC, in the multivariable linear regression models. We adopted this approach to ensure that both statistically significant and contextually important variables were considered in the adjusted models. To determine whether the factors associated with FoC differed by parity, stratified multivariable linear regression models were fitted to identify factors that were independently associated with nulliparous and parous women. We set the statistical significance threshold at p < 0.05, corresponding to a 95% confidence interval. The Variance Inflation Factor (VIF) was used to assess multicollinearity among independent variables for all the multivariable models (overall, nulliparous-specific, and parous-specific). The mean VIFs for the overall, nulliparousspecific, and parous-specific models were 2.44 (range: 1.17 to 4.80), 2.94 (range: 1.48 to 6.86), and 3.02 (range: 1.10 to 6.97), respectively. All mean VIFs were below the conventional threshold of 10, indicating no significant multicollinearity; however, some individual variables had high VIFs. Specifically, in the nulliparous-specific model, tertiary education had a VIF of 6.86, and in the parous-specific model, occupation (formal: 6.97; informal: 6.08) had high VIFs. These variables were retained in the models due to their theoretical significance and their minimal to no effect on estimates of other variables.

# Ethical approval

This study was conducted in accordance with the ethical principles outlined in the Declaration of Helsinki. The Committee on Human Research, Publications, and Ethics (CHRPE), School of Medicine and Dentistry, Kwame Nkrumah University of Science and Technology (KNUST), Kumasi (CHRPE/AP/025/25) granted ethical approval for this study. Written approval was sought from the study site's management. Before recruitment, all study participants went through a mandatory informed consent process and signed an informed consent form. As part of the consent process, all study participants were provided with information about the study's objectives, significance, and potential risks of participation.

**Results**

# Socio-demographic and clinical characteristics of the study participants stratified by parity status

A total of 282 of the 290 women recruited participated in the study, yielding a response rate of 97.2%. Of this, 35.1% were nulliparous, while the remaining 64.9% were parous. The overall mean age of the study participants was 28 (±5.5) years, with nulliparous women (23.9 ± 3.9 years) being significantly younger than parous women (30.2 ± 5.0 years). Approximately 45.5% of the nulliparous women were single, compared to 8.7% of the parous women. While both groups had relatively high levels of tertiary education, parous women had a slightly higher level (48.1%) than nulliparous women (45.6%). A little over half (50.8%) of parous women had an informal occupation, compared to 24.2% of nulliparous women. The level of family support was similar across the two groups of women (Table 1).

# Table 1: Socio-demographic and clinical characteristics of the study participants stratified by parity

**Characteristics	Parity	Overall,	p-value Nulliparous,	Parous,**

|  | **N = 99 (35.1)** | **N = 183 (64.9)** | **N = 282** |  |
| --- | --- | --- | --- | --- |
| Age (mean ±SD) *^1^* | 23.9 (±3.9) | 30.2 (±5.0) | 28 (±5.5) | **<0.001** |
| **Marital status** |  |  |  | **<0.001** |
| Single | 45 (45.5) | 16 (8.7) | 61 (21.6) |  |
| Married | 38 (38.4) | 126 (68.9) | 164 (58.2) |  |
| Co-habiting | 16 (16.2) | 41 (22.4) | 57 (20.2) |  |
| **Education level** |  |  |  | **0.004** |
| Basic | 9 (9.1) | 41 (22.4) | 50 (17.7) |  |
| Secondary | 45 (45.5) | 54 (29.5) | 99 (35.1) |  |
| Tertiary | 45 (45.6) | 88 (48.1) | 133 (47.2) |  |
| **Occupation** |  |  |  | **<0.001** |
| Unemployed | 39 (39.4) | 9 (4.9) | 48 (17.0) |  |
| Formal | 36 (36.4) | 81 (44.3) | 117 (41.5) |  |
| Informal | 24 (24.2) | 93 (50.8) | 117 (41.5) |  |
| **Religion** |  |  |  | 0.397 |
| Christian | 77 (77.8) | 150 (82.0) | 227 (80.5) |  |
| Muslim | 22 (22.2) | 33 (18.0) | 55 (19.5) |  |
| **Head of household** |  |  |  | **<0.001** |
| Partner | 71 (71.7) | 177 (96.7) | 248 (87.9) |  |
| Self | 9 (9.1) | 6 (3.3) | 15 (5.3) |  |
| Mother/Father | 19 (19.2) | 0 (0.0) | 19 (6.7) |  |
| **Experienced intimate partner violence** |  |  |  | 0.056 |
| Yes | 9 (9.1) | 32 (17.5) | 41 (14.5) |  |
| No | 90 (90.9) | 151 (82.5) | 241 (85.5) |  |
| **History of abuse in the family** |  |  |  | 0.585 |
| Yes | 22 (22.2) | 46 (25.1) | 68 (24.1) |  |
| No | 77 (77.8) | 137 (74.9) | 214 (75.9) |  |
| **ANC booking (weeks)** |  |  |  | 0.384 |
| Early (1 – 12) | 71 (71.2) | 122 (66.7) | 193 (68.4) |  |
| Late (13+) | 28 (28.3) | 61 (33.3) | 89 (31.6) |  |
| **ANC booking depends on partner’s decision** |  |  |  | 0.260 |
| Yes | 21 (21.2) | 29 (15.9) | 50 (17.7) |  |
| No | 78 (78.8) | 154 (84.2) | 232 (82.3) |  |
| **Planned the current pregnancy** |  |  |  | 0.493 |
| Yes | 45 (45.5) | 91 (49.7) | 136 (48.2) |  |
| No | 54 (54.6) | 92 (50.3) | 146 (51.8) |  |
| **History of abortion** |  |  |  | **0.001** |

| Yes | 36 (36.4) | 34 (18.6) | 70 (24.8) |  |
| --- | --- | --- | --- | --- |
| No | 63 (63.6) | 149 (81.4) | 212 (75.2) |  |
| **Family’s support** |  |  |  | 0.746 |
| Poor | 25 (25.3) | 40 (21.9) | 65 (23.1) |  |
| Moderate | 33 (33.3) | 68 (37.2) | 101 (35.8) |  |
| Strong | 41 (41.4) | 75 (41.0) | 116 (41.1) |  |

229	*^1^*Continuous variables reported as mean (±SD); all others as n (%)

230

## 231 **Prevalence of FoC among study participants**

232 Overall, the prevalence of FoC among the study participants was 22.0% (≥66) (Figure 1). The 233 median W-DEQ score among the study participants was 41 (interquartile range [IQR]: 33 – 234 61). When stratified by parity, nulliparous women (median: 46.0, IQR: 37.0–66.0) reported 235 significantly higher median W-DEQ scores than parous women (median: 39.0, IQR: 32.0– 236 58.5). Regarding the severity of FoC, nulliparous women reported significantly higher levels 237 of FoC compared to parous women, with a greater proportion experiencing severe fears (16.2% 238 vs 10.4%) (Table 2).

239

240	**Table 2: Prevalence of FoC among study participants stratified by parity**

# Characteristics Nulliparous, Parous,

**Parity**

**Overall,**

**p**

**-**

**value**

|  | | **N = 99** | **N = 183** | **N = 282** |  |
| --- | --- | --- | --- | --- | --- |
| **W-DEQ** Median (IQR) | | 46.0 (37.0–66.0) | 39.0 (32.0–58.5) | (33 – 61) | **0.004** |
| **Levels of FoC, n (%)** Low | | (28.3) | (45.4) | (39.4) | **0.039** |
| Moderate | | (45.5) | (35.0) | (38.7) |  |
| High | | (10.1) | (9.3) | (9.6) |  |
| Severe | | (16.2) | (10.4) | (12.4) |  |
| IQR: Interquartile range |  |  |  |  |  |

## 241 242

## 243

Figure: Prevalence of FoC among study participants

# Factors associated with FoC among study participants

In the unadjusted model, factors such as age (β**:** -1.25, 95%CI: -1.74, 0.77), being married (β**:** -27.13, 95%CI: -33.27, -20.98), having formal employment (β**:** -25.34, 95%CI: -32.72, -17.95), experiencing intimate partner violence (β**:** 29.42, 95% CI: 22.26, 36.57), booking ANC late (β**:** 20.82, 95%CI: 15.32, 26.33), planning current pregnancy (β**:** 25.71, 95%CI: 20.99, 30.43) and strong family’s support (β**:** -36.27, 95%CI: -42.18, -30.36) were significantly associated with FoC. In the adjusted model, factors such as being in a cohabiting relationship (adj. β**:** -8.74, 95%CI: -15.23, -2.25), having secondary (adj. β**:** -7.58, 95%CI: -13.58, -1.58) and tertiary education (Adj. β**:** -9.44, 95%CI: -17.65, -1.23), having informal occupation (adj. β**:** -7.88, 95%CI: -14.83, -0.92), having history of abuse in the family (adj. β**:** 10.38, 95%CI: 4.47, 16.28), late ANC booking (adj. β**:** 6.32, 95%CI: 1.67, 10.96), not planning the current pregnancy (adj. β**:** 8.84, 95%CI: 3.72, 13.96) and having moderate (adj. β**:** -13.10, 95%CI: 18.70, -7.50): and strong (adj. β**:** -19.87, 95%CI: -26.53, -13.22) family’s support remained significantly associated with FoC after adjusting for other covariates (Table 3).

In the parity-stratified analysis, among nulliparous women, factors such as having a history of family abuse (adj. β**:** 16.32, 95%CI: 2.50, 30.14) and late ANC booking (adj. β**:** 13.13, 95%CI: 1.43, 24.84) were significantly associated with FoC. Among parous women, factors such as having secondary education (adj. β**:** -7.63, 95%CI: -14.88, -0.37), experiencing intimate partner violence (adj. β**:** 10.87, 95%CI: 0.69, 21.04), not planning current pregnancy (adj. β**:** 8.38, 95%CI: 2.51, 14.26), and having moderate (adj. β**:** -23.10 (-31.91, -14.30) and strong (adj. β**:**

-27.94, 95%CI: -38.77, -17.11) family support was significantly associated with FoC (Table 4).

| **Characteristics** | **β (95% CI)** | **p-value** | **Adj. β (95% CI)** | **p-value** |
| --- | --- | --- | --- | --- |
| Age | -1.25 (-1.74, 0.77) | **<0.001** | -0.35 (-0.86, 0.16) | 0.182 |
| Parity |  |  |  |  |
| Nulliparous | 1.00 |  | 1.00 |  |
| Parous | -7.78 (-13.80, -1.75) | **0.012** | -3.35 (-8.81, 2.11) | 0.228 |
| **Marital status** |  |  |  |  |
| Single | 1.00 |  | 1.00 |  |
| Married | -27.13 (-33.27, -20.98) | **<0.001** | 3.35 (-10.03, 3.33) | 0.324 |
| Co-habiting | -8.02 (-15.56, -0.47) | **0.038** | -8.74 (-15.23, -2.25) | **0.009** |
| **Education level** |  |  |  |  |
| Basic | 1.00 |  | 1.00 |  |
| Secondary | -11.94 (-19.08, -4.80) | **0.001** | -7.58 (-13.58, -1.58) | **0.014** |
| Tertiary | -29.65 (-36.48, -22.82) | **<0.001** | -9.44 (-17.65, -1.23) | **0.024** |
| **Occupation** |  |  |  |  |
| Unemployed | 1.00 |  | 1.00 |  |
| Formal | -25.34 (-32.72, -17.95) | **<0.001** | -5.65 (-12.61, 1.31) | 0.111 |
| Informal | -9.89 (-17.28, -2.51) | **0.009** | -7.88 (-14.83, -0.92) | **0.027** |
| **Religion** |  |  |  |  |
| Christian | 1.00 |  | 1.00 |  |
| Muslim | 4.39 (-2.66, 11.44) | 0.221 | -3.50 (-8.73, 1.74) | 0.190 |
| **Experienced intimate partner violence** |  |  |  |  |
| No | 1.00 |  | 1.00 |  |
| Yes | 29.42 (22.26, 36.57) | **<0.001** | 6.59 (-0.64, 13.82) | 0.074 |
| **History of abuse in the family** |  |  |  |  |
| No | 1.00 |  | 1.00 |  |
| Yes | 26.51 (20.75, 32.27) | **<0.001** | 10.38 (4.47, 16.28) | **0.001** |
| **ANC booking (weeks)** |  |  |  |  |
| Early (1 – 12 | 1.00 |  | 1.00 |  |
| Late (13+) | 20.82 (15.32, 26.33) | **<0.001** | 6.32 (1.67, 10.96) | **0.008** |
| **Planned the current pregnancy** |  |  |  |  |
| Yes | 1.00 |  | 1.00 |  |
| No | 25.71 (20.99, 30.43) | **<0.001** | 8.84 (3.72, 13.96) | **0.001** |
| **History of abortion** |  |  |  |  |
| No | 1.00 |  | 1.00 |  |
| Yes | 3.37 (-3.11, 9.84) | 0.307 | -2.14 (-6.82, 2.54) | 0.369 |
| **Family’s support** |  |  |  |  |
| Poor | 1.00 |  | 1.00 |  |
| Moderate | -20.44 (-26.50, -14.38) | **<0.001** | -13.10 (-18.70, -7.50) | **<0.001** |
| Strong | -36.27 (-42.18, -30.36) | **<0.001** | -19.87 (-26.53, -13.22) | **<0.001** |

# Table 3: Linear regression analysis of factors associated with FoC among study participants

271 β = unstandardized regression coefficient; CI = confidence interval; 1:00 = reference category;
272 Adj. β: adjusted regression coefficient
273 274

275 **Table 4: Stratified multivariable linear regression analysis of factors associated with FoC** 276 **by parity status**

# Characteristics Nulliparous Parous

**Adj. β (95% CI)	p-value	Adj. β (95% CI)	p-value**

Age	-0.69 (-3.00, 1.62)	0.556	-0.10 (-0.62, 0.42)	0.714

# Marital status

Single	1.00	1.00

Married	-8.06 (-18.61, 2.49)	0.133	-0.96 (-10.94, 9.02)	0.850

Co-habiting	-4.34 (-14.40, 5.71)	0.393	-7.36 (-17.72, 3.01)	0.163

# Education level

Basic	1.00	1.00

Secondary	-12.16 (-32.65, 8.33)	0.241	-7.63 (-14.88, -0.37)	**0.039**

Tertiary	-16.91 (-40.83, 7.01)	0.164	-8.54 (-20.72, 3.17)	0.152

# Occupation

Unemployed 1.00 1.00 Formal -1.24 (-10.59, 8.10) 0.792 -4.41 (-13.14, 4.33) 0.321

Informal	-3.27 (-13.79, 7.25)	0.538	-7.48 (-15.24, 0.29)	0.059

# Experienced intimate partner violence

No	1.00	1.00

Yes	4.83 (-14.56, 24.22)	0.622	10.87 (0.69, 21.04)	**0.037**

# History of abuse in the family

No	1.00	1.00

Yes	16.32 (2.50, 30.14)	**0.021**	6.22 (-1.67, 14.11)	0.121

# ANC booking (weeks)

Early (1 – 12)	1.00	1.00

Late (13+)	13.13 (1.43, 24.84)	**0.028**	3.20 (-2.17, 8.58)	0.241

# Planned the current pregnancy

Yes	1.00	1.00

No	-3.69 (-15.07, 7.69)	0.521	8.38 (2.51, 14.26)	**0.005**

# History of abortion

No	1.00	1.00

Yes	-4.05 (-12.36, 4.26)	0.335	4.56 (-1.75, 10.87)	0.155

# Family’s support

Poor	1.00	1.00

Moderate	5.06 (-6.32, 16.44)	0.379	-23.10 (-31.91, -14.30)	**<0.001**

## Strong -9.06 (-19.52, 1.39) 0.088 -27.94 (-38.77, -17.11) **<0.001**

277 β = unstandardized regression coefficient; CI = confidence interval; 1:00 = reference category;
278 Adj. β: adjusted regression coefficient
279 280	281

# Discussions

This study sought to examine the prevalence and determinants of FoC among pregnant women attending ANC in a primary healthcare facility in Kumasi, Ghana, with a specific focus on parity (i.e., differences between nulliparous and parous women). We found that FoC is relatively common, with nulliparous women reporting higher fears than parous women, and approximately 22% experiencing high to severe FoC. Our findings underpin a critical need to screen for FoC during routine ANC, especially in settings with limited psychological support.

We found that FoC was substantial in this cohort. Our finding that approximately 22% of the participants experienced high to severe FoC (W-DEQ scores ≥66) is consistent with studies from Malawi (20%) and Australia (24%) (28,29), but lower than the rates reported in Kenya (30.1%), China (35.8%), and the Netherlands (33.9%) (10,24,30). Additionally, the median WDEQ score in our study was 41, comparable to a similar survey in Malawi (mean: 47), but lower than what has been documented in high-income countries (21,31,32). This variability may reflect differences in population characteristics, healthcare systems, cultural perceptions of childbirth, availability of psychosocial support, and methodological disparities, including skewness in the distribution of W-DEQ scores. This suggests that the FoC experience is not uniform and varies across populations.

Nulliparous women experienced significantly higher fears than parous women, consistent with a previous study conducted in Finland and other low- and middle-income countries (33–35). A plausible explanation could be that first-time mothers lack previous birth experience, leading to uncertainty and perceived higher pain risk. In contrast, parous women, having experienced childbirth, may develop coping mechanisms that reduce the fear.

Consistent with previous research, our findings demonstrate that nulliparous women were more likely to be younger, single, and experiencing childbirth for the first time (36). These characteristics have been consistently associated with increased FoC. For instance, Duong *et al.* found that nulliparous women tend to have greater FoC due to the unpredictability and novelty of the experience (37). Additionally, the association between a history of family abuse and higher FoC observed in our nulliparous subgroup is supported by earlier studies by Goldstein *et al.* and Van Parys *et al*. (38,39). Addressing abuse-related trauma through antenatal counselling represents a critical intervention point to mitigate FoC. Another key finding in nulliparous women was the association between late ANC booking and increased FoC. Late

ANC booking may limit access to early interventions, including education and support that could alleviate these fears. Research suggests that nulliparous women who engage in timely ANC are better prepared for childbirth, which subsequently helps to reduce their fear (40).

Among parous women, our findings revealed that higher educational attainment, particularly completion of secondary education, is associated with lower levels of FoC than a basic education, consistent with studies from Iran and Turkey (41,42). This trend suggests that education enhances knowledge, confidence, and facilitates communication with healthcare providers (43–45). The observed association between IPV, unplanned pregnancy, and FoC among parous women is well-established (46–49), with significant implications for maternal health. Our study contributes to this body of knowledge by demonstrating that this relationship persists even after adjusting for other factors, underscoring the need for psychosocial support and reproductive counselling.

Another critical factor was the inverse association between family support and FoC, underscoring the broader literature emphasising the protective role of social support on maternal anxiety and childbirth-related stress (50,51). Women with strong support systems tend to feel more empowered, informed, and emotionally secure, which often results in reduced levels of fear and anxiety (52). This finding underscores the importance of social networks in improving childbirth experiences in Ghana and advances understanding of the psychosocial factors that influence FoC in resource-limited settings. The protective role of family support increases the understanding of how social variables shape FoC. This highlights the importance of integrating family-oriented approaches into ANC services to reduce FoC and improve maternal well-being*.* This finding is particularly relevant in the Ghanaian context, where extended family structures and community networks play a crucial role in shaping women’s health experiences. Encouraging family involvement in ANC and advocating for supportive environments may be essential to reducing FoC.

# Implications for Practice and Research

The findings of this study have important implications for maternal healthcare delivery in Ghana and similar low-resource settings. Our findings highlight the need for parity-specific interventions. Specifically, nulliparous women may benefit from structured antenatal education and early ANC booking, while psychosocial counselling, IPV screening, and family support may be helpful in parous mothers.

Our findings suggest that, in addition to routine ANC programs that screen for FoC, they should also integrate patient-centred strategies that account for the unique risk profiles within each parity group. Healthcare managers need to train professionals to identify at-risk populations, provide patient-centred education, and involve family members when necessary to promote social support. Incorporating FoC screening tools into routine ANC guidelines could help identify vulnerable women who could benefit from additional psychological or social support strategies, thereby enhancing maternal satisfaction and childbirth preparedness.

Future research should investigate the effectiveness of culturally tailored FoC-reduction interventions, including peer support groups, mobile-based health education tools, and traumainformed care. Longitudinal studies are also needed to track changes in FoC across pregnancy and postpartum, and its influence on maternal outcomes.

# Strengths and Limitations of the Study

A key strength of this study is its stratified analytical approach, which enabled an in-depth comparison of FoC determinants across parity groups. This methodological decision enhances the utility of the findings for developing tailored interventions. The study also employed the W-DEQ, a widely validated tool for assessing FoC, thereby strengthening the study's internal validity.

However, several limitations must be noted. First, the study was conducted in a single primary healthcare facility, which may limit the generalizability of the findings to other regions or facility types in Ghana. Second, some variables, such as IPV, family abuse, and family support, were measured using a single-item self-report, which may reduce measurement breadth and precision that could have been examined with multiple items. Again, participants' gestational age could have influenced their FoC. However, gestational age was not collected as a study variable and therefore could not be included in the model. Consequently, we acknowledge it as a limitation of the study. Finally, although the sample size was adequate for the primary analyses, it may not have been powered to detect minor effects or subgroup interactions.

Despite these limitations, the study provides valuable insights into the psychological experiences of pregnant women in a resource-constrained setting and identifies clear intervention points to address FoC.

# Conclusion

The current study highlights that FoC is a significant psychological burden among pregnant women in Kumasi, Ghana, with approximately one in five women reporting high or severe levels of fear. The determinants of FoC varied by parity: nulliparous women were more affected by abuse and late ANC booking, while parous women’s fear was influenced by education level, intimate partner violence, unplanned pregnancy, and perceived family support.

Our findings contribute to understanding FoC in low-resource settings by identifying parityspecific risk profiles that have previously gone unexplored in Ghana. These findings underscore the need for parity-specific strategies in addressing FoC during ANC. Routine screening for FoC, early ANC engagement, psychosocial support, and family-inclusive care models could collectively reduce childbirth-related fear and promote better maternal health outcomes. Future research should explore these strategies using longitudinal, interventional, and qualitative designs to guide policy and practice in Ghana and similar settings.

# Acknowledgement

For their important contributions to this study, we would especially like to thank the management and staff of the HopeXchange Medical Centre, the participants, and our research assistants.

# Author Contributions

Conceptualisation: JOA, DAO & EAB

Data curation: JOA, DAO, MA, EK, FKK, & JKK

Formal analysis: DAO, MA, EK, & JKK

Methodology: DAO, EAB, JOA, MK, OKOA, & FKK

Project administration: JOA, DAO, MK, OKOA, & EAB

Supervision: EAB

Validation: DAO, MA, EK, MK, JOA, OKOA, FKK, EAB & JKK

Writing – original draft: DAO, MA, EK, & JKK

Writing – review and editing: JOA, DAO, MK, OKOA, JKK, MA, EK, FKK & EAB

**Competing interest**

The authors have declared that no competing interests exist.

**Funding**

The authors received no specific funding for this work.

# References

1. Chen C, Hussein SZB, Nasri NWM, Yao J, Qin Y, Zhao Z, et al. Fear of childbirth among pregnant women: A concept analysis. J Adv Nurs. 2024;80(11):4476–87.
2. Wigert H, Nilsson C, Dencker A, Begley C, Jangsten E, Sparud-Lundin C, et al. Women’s experiences of fear of childbirth: a metasynthesis of qualitative studies. Int J Qual Stud Health Well-being. 2020;15(1).
3. Hofberg K, Ward MR. Fear of pregnancy and childbirth. Postgrad Med J. 2003;79(935):505–10.
4. O’Connell MA, Leahy-Warren P, Khashan AS, Kenny LC, O’Neill SM. Worldwide prevalence of tocophobia in pregnant women: systematic review and meta-analysis. Acta Obstet Gynecol Scand. 2017;96(8):907–20.
5. Li B, Liu T, Ma D, Sun J, Liu J. Association of fear of childbirth and postpartum depression with perceived partner response during pregnancy. BMC Pregnancy Childbirth. 2025;25(1):1–9.
6. Ali MH, Seif SA, Kibusi SM. The Influence of Fear During Pregnancy, Labour and Delivery on Birth Outcome Among Post-Delivery Women: A Case Control Study in Zanzibar. East African Heal Res J. 2022;6(2):147–54.
7. Elgzar WT, Alshahrani MS, Ibrahim HAF. Mode of delivery preferences: the role of childbirth fear among nulliparous women. Front Psychol. 2023;14(November):1–10.
8. Rúger-Navarrete, A., Vázquez-Lara, J.M., Antúnez-Calvente, I., Rodríguez-Díaz, L., Riesco-González, F.J., Palomo-Gómez, R., Gómez-Salgado, J. and Fernández-Carrasco, FJ. Antenatal fear of childbirth as a risk factor for a bad childbirth experience. Healthcare. 2023;11(3):1–13.
9. Massae AF, Larsson M, Pembe AB, Mbekenga C, Svanberg AS. Patterns and predictors of fear of childbirth and depressive symptoms over time in a cohort of women in the Pwani region, Tanzania. PLoS One. 2022;17(11 November).
10. Onchonga D, MoghaddamHosseini V, Keraka M, Várnagy Á. Prevalence of fear of childbirth in a sample of gravida women in Kenya. Sex Reprod Healthc. 2020;24:100510.
11. Khwepeya M, Lee GT, Chen SR, Kuo SY. Childbirth fear and related factors among pregnant and postpartum women in Malawi. BMC Pregnancy Childbirth.

2018;18(1):391.

12. Berhanu RD, Abathun AD, Negessa EH, Amosa LG. The magnitude and associated factors of childbirth fear among pregnant women attending antenatal care at public hospitals in Ethiopia: a cross-sectional study. BMC Pregnancy Childbirth. 2022;22(1).
13. Charan J, Biswas T. How to calculate sample size for different study designs in medical research? Vol. 35, Indian Journal of Psychological Medicine. 2013. p. 121–6.
14. Hildingsson I, Haines H, Karlström A, Nystedt A. Presence and process of fear of birth during pregnancy — Findings from a longitudinal cohort study. Women and Birth. 2017;(2016):2–7.
15. Phunyammalee M, Buayaem T, Boriboonhirunsarn D. Fear of childbirth and associated factors among low-risk pregnant women. J Obstet Gynaecol (Lahore). 2019;39(6):763–

7.

16. O’Connell MA, Leahy‐Warren P, Kenny LC, O’Neill SM, Khashan AS. The prevalence and risk factors of fear of childbirth among pregnant women: A cross‐sectional study in Ireland. Acta Obstet Gynecol Scand. 2019;98(8):1014–23.
17. Dencker A, Nilsson C, Begley C, Jangsten E, Mollberg M, Patel H, et al. Causes and outcomes in studies of fear of childbirth: A systematic review. Women and Birth. 2019;32(2):99–111.
18. Räisänen S, Lehto SM, Nielsen HS, Gissler M, Kramer MR, Heinonen S. Fear of childbirth in nulliparous and multiparous women: a population‐based analysis of all singleton births in Finland in 1997–2010. BJOG An Int J Obstet Gynaecol. 2014;121(8):965–70.
19. He D, Zhang J, Wang G, Huang Y, Li N, Zhu M, et al. Prevalence and factors associated with fear of childbirth in late pregnancy: a cross-sectional study. Front Public Heal.

2025;13:1589568.

20. Dal Moro APM, Soecki G, de Fraga FS, Petterle RR, Rückl SZ. Fear of childbirth: prevalence and associated factors in pregnant women of a maternity hospital in southern Brazil. BMC Pregnancy Childbirth. 2023;23(1):1–12.
21. Lukasse M, Schei B, El R. Prevalence and associated factors of fear of childbirth in six European countries. Sex Reprod Healthc. 2014;5(3):99–106.
22. Wjma K. Psychometric aspects of the W-DEQ ; a new questionnaire for the measurement of fear of childbirth. J Psychosom Obstet Gynecol. 1998;19(2):84–97.
23. Massae AF, Larsson M, Leshabari S, Mbekenga C, Pembe AB, Svanberg AS. Predictors of fear of childbirth and depressive symptoms among pregnant women: a cross-sectional survey in Pwani region, Tanzania. BMC Pregnancy Childbirth. 2021;21(1):1–13.
24. Fang W, Jiang H, Chen A. The impact of fear of childbirth on mode of delivery, postpartum mental health and breastfeeding: A prospective cohort study in Shanghai, China. J Affect Disord. 2024;347(May 2023):183–91.
25. Jing Q, Jie J, Ke X, Liu Y, Xiumin D, Xiuchuan L, et al. Developmental trajectories of maternal fear of childbirth at different times: a prospective longitudinal study. BMC Women's Health. 2025;25:325.
26. Adams SS, Eberhard-Gran M, Eskild A. Fear of childbirth and duration of labour: A study of 2206 women with intended vaginal delivery. BJOG An Int J Obstet Gynaecol.

2012;119(10):1238–46.

27. Alehagen S, Wijma B, Wijma K. Fear of childbirth before, during, and after childbirth. Acta Obstet Gynecol Scand. 2006;85(1):56–62.
28. Khwepeya M, Lee GT, Chen SRSR, Kuo SYSY. Childbirth fear and related factors among pregnant and postpartum women in Malawi. BMC Pregnancy Childbirth. 2018;18(1):391.
29. Toohill J, Fenwick J, Gamble J, Creedy DK. Prevalence of childbirth fear in an Australian sample of pregnant women. BMC Pregnancy Childbirth. 2014;14:275.
30. Veringa-Skiba IK, Bruin EI De, Mooren B, Steensel FJA Van, Bögels SM. Can a simple assessment of fear of childbirth in pregnant women predict requests and use of nonurgent obstetric interventions during labour?. Midwifery. 2021;97(May 2020):102969.
31. Hall WA, Hauck YL, Carty EM, Hutton EK, Fenwick J, Stoll K. Childbirth fear, anxiety, fatigue, and sleep deprivation in pregnant women. J Obstet Gynecol Neonatal Nurs. 2009;38(5):567–76.
32. Fenwick J, Gamble J, Nathan E, Bayes S, Hauck Y. Pre-and postpartum levels of childbirth fear and the relationship to birth outcomes in a cohort of Australian women. J Clin Nurs. 2009;18(5):667–77.
33. Oladele OC, Akinwaare MO, Ogbeye GB, Oluseye O. Childbirth fear and birth-related mindset of first-time mothers who registered for antenatal care in public hospitals in Nigeria. Int J Childbirth. 2024;14(4).
34. Mandar O, Idrees MB, Ahmed A, ALhabardi N, Hassan B, Adam I. Prevalence and associated factors of fear for childbirth among pregnant women in eastern Sudan. J Reprod Infant Psychol. 2023;41:319–29.
35. Rouhe H, Salmela-Aro K, Halmesmäki E, Saisto T. Fear of childbirth according to parity, gestational age, and obstetric history. BJOG An Int J Obstet Gynaecol. 2009;116(1):67–73.
36. Geidam A. Pregnancy outcome among nulliparous women at the University of Maiduguri Teaching Hospital, North Eastern Nigeria: a retrospective cohort study. Br J Med Med Res. 2014;4:3893–901.
37. Duong HT, Hoyt AT, Carmichael SL, Gilboa SM, Canfield MA, Case A, et al. Is maternal parity an independent risk factor for birth defects? Birth Defects Res. 2012;94:230–6.
38. Goldstein BL, Briggs-Gowan MJ, Grasso DJ. The effects of intimate partner violence and a history of childhood abuse on mental health and stress during pregnancy. J Fam Violence. 2021;36(3):337–46.
39. Van Parys AS, Deschepper E, Michielsen K, Galle A, Roelens K, Temmerman M, et al. Intimate partner violence and psychosocial health, a cross-sectional study in a pregnant population. BMC Pregnancy Childbirth. 2015;15:278.
40. Bektas Pardes B, Guvenc G. The effects of antenatal education and telephone counseling on childbirth fear of nulliparous women and their attitudes toward childbirth: a randomized controlled trial. Rev Assoc Med Bras. 2025;71:e20241147.
41. Soysal C, Ulaş Ö, Işıkalan MM, Bıyık İ, Taşçı Y, Keskin N. The changes in fear of childbirth in pregnancy during and before the COVID-19 pandemic. Sci Rep. 2024;14:11067.
42. Barat S, Kordinejad B, Faramarzi M, Khafri S, Bouzari Z, Ebrahim E. Prevalence of fear of childbirth and its effective factors in pregnant women in Babol, Iran (2019-2020): a cross-sectional study. Int J Women’s Heal Reprod Sci. 2023;11(1):25–32.
43. Ketema DB, Leshargie CT, Kibret GD, Assemie MA, Petrucka P, Alebel A. Effects of maternal education on birth preparedness and complication readiness among Ethiopian pregnant women: A systematic review and meta-analysis. BMC Pregnancy Childbirth. 2020;20(1):1–9.
44. Amponsah E, Fusheini A, Adam A. Influence of information, education and communication on prenatal and skilled delivery in the Tano North District, Ghana: A cross-sectional study. Heliyon. 2021;7(6):e07245.
45. Greenaway ES, Leon J, Baker DP. Understanding the association between maternal education and use of health services in Ghana: Exploring the role of health knowledge. J Biosoc Sci. 2012;44(6):733–47.
46. Adu-Bonsaffoh K, Tamma E, Seffah JD. Prevalence and determinants of unintended pregnancy among women receiving antenatal care services: a facility-based crosssectional study in Ghana. Glob J Health Sci. 2021;13(9):100.
47. Alain DC, Nidelle TA, Evrard KN, Franck KF, Armand NNW, Sadrack A, et al. Domestic violence during pregnancy and risk of obstetrical complications. African Clin Res Innov. 2024;24:142–55.
48. Eliason S, Baiden F, Yankey BA, Awusabo-Asare K. Determinants of unintended pregnancies in rural Ghana. BMC Pregnancy and Childbirth. 2014;14(1). 261&partnerID=40&md5=32f6a261400d8df1c8fb50cd1fad5f96
49. Oğurlu M, Erbil N. The effect of intimate partner violence on fear of childbirth among pregnant women. J Interpers Violence. 2023;38(3–4):3737–55.
50. Pinar SE, Aksoy OD, Cesur B, Bilgic D, Daglar G, Guler E. Investigation of the relationship between fear of childbirth and social supports of pregnant women in the third trimester in Turkey. Clin Exp Obstet Gynecol. 2017;44:767–71.
51. Baheiraei A, Mirghafourvand M, Mohammadi E, Charandabi SMA, Nedjat S. Social
558 support for women of reproductive age and its predictors: a population-based study.
559 BMC Women's Health. 2012;12:30.
560 52.	Acoba EF. Social support and mental health: the mediating role of perceived stress.
561 Front Psychol. 2024;15(February):1–12.

562

## 563 **Supporting information**

564	Datasets used for the analysis.

### 565

dataset for analysis

[Click here to access/downloa](https://www2.cloud.editorialmanager.com/pone/download.aspx?id=44434977&guid=01b63c1d-4902-46f9-b581-02c88c483cb7&scheme=1)

[d](https://www2.cloud.editorialmanager.com/pone/download.aspx?id=44434977&guid=01b63c1d-4902-46f9-b581-02c88c483cb7&scheme=1)

[Supporting Informatio](https://www2.cloud.editorialmanager.com/pone/download.aspx?id=44434977&guid=01b63c1d-4902-46f9-b581-02c88c483cb7&scheme=1)

[n](https://www2.cloud.editorialmanager.com/pone/download.aspx?id=44434977&guid=01b63c1d-4902-46f9-b581-02c88c483cb7&scheme=1)

[foc.xl](https://www2.cloud.editorialmanager.com/pone/download.aspx?id=44434977&guid=01b63c1d-4902-46f9-b581-02c88c483cb7&scheme=1)

[s](https://www2.cloud.editorialmanager.com/pone/download.aspx?id=44434977&guid=01b63c1d-4902-46f9-b581-02c88c483cb7&scheme=1)

Revised Manuscript with Track Changes

## 1 **Fear of childbirth among antenatal care attendees in a primary care setting in Kumasi,** 2 **Ghana: Prevalence and parity-stratified determinants**

3

4 **Authors;** Juliana Ofosua Addo*^1,2^, Douglas Aninng Opoku*^3,4^, Julius Kwabena Karikari^3~~,4~~^,
5 Emmanuel Konadu^3~~,4~~^, Mercy Addae^3,4^, Francis Kwabena Kyei^1^, Mawuse Kanfra^5^, Obed 6 Kwabena Offe Amponsah^6^, & Eric Adjei Boadu^1^

7

## 8 **Affiliations;**

9 ^1^Department of Population, Family and Reproductive Health, School of Public Health, Kwame
10 Nkrumah University of Science and Technology, Kumasi, Ghana
11 ^2^HopeXchange Medical Centre, Kumasi, Ghana
12 ^3^University Hospital, Kwame Nkrumah University of Science and Technology, Kumasi, Ghana
13 ^4^Department of Epidemiology and Biostatistics, School of Public Health, Kwame Nkrumah
14 University of Science and Technology, Kumasi, Ghana
15 ^5^St. Dominic Catholic Hospital, Akwatia, Ghana
16 ^6^Department of Pharmacy Practice, Faculty of Pharmacy and Pharmaceutical Sciences, Kwame 17 Nkrumah University of Science and Technology, Kumasi, Ghana

### 18

19 *Corresponding author: Douglas Aninng Opoku: douglasopokuaninng@gmail.com; Juliana 20 Ofosuaa Addo: julianaaddo@gmail.com

21

### 22 23

24	25	26	27	28	29	30

# Abstract

**Background:** Previous studies on Fear of Childbirth (FoC) across sub-Saharan Africa have typically reported prevalence separately by parity or treated it as a covariate in multivariable analyses, with little attention given to ~~identifying-parity-specific~~identifying parity-specific determinants. ~~Howerver~~However, first-time mothers and those with previous birth experiences may require different forms of antenatal support. To address this gap, we assessed the prevalence of FoC and identified parity-specific determinants among antenatal care attendees in Ghana to inform targeted interventions.

**Methods:** A facility-based cross-sectional study was carried out among pregnant women attending antenatal care at a primary healthcare facility in Kumasi. Data were collected using an interviewer-administered questionnaire. The primary outcome of the study, FoC, was assessed using the English version of the Wijma Delivery Expectation/Experience Questionnaire (W-DEQ). Additional data included sociodemographic and obstetric characteristics. Stratified multivariable linear regression models were applied to identify parity-specific determinants among the participants.

**Results:** Of the 282 women who participated in the study, 22% had high-to-severe FoC. Nulliparous women (median: 46.0, IQR: 37.0–66.0) reported significantly higher median WDEQ scores than parous women (median: 39.0, IQR: 32.0–58.5). In a parity-stratified analysis, among nulliparous women, factors such as a history of family abuse (adj. β: 16.32, 95%CI: 2.50, 30.14) and late ANC booking (adj. β: 13.13, 95%CI: 1.43, 24.84) were significantly associated with FoC. Among parous women, factors such as secondary education (adj. β: -7.63, 95%CI: -14.88, -0.37), intimate partner violence (adj. β: 10.87, 95%CI: 0.69, 21.04), unplanned pregnancy (adj. β: 8.38, 95%CI: 2.51, 14.26), and strong family support (adj. β: -27.94, 95%CI: -38.77, -17.11) were significantly associated with FoC.

**Conclusion:** FoC is common among this cohort of pregnant women, and its determinants differed by parity and across parity-specific factors. The parity-stratified determinants underscore the need for parity-tailored psychosocial care at ANC, incorporating targeted screening and support procedures aligned with parity-associated risk pathways to enhance maternal well-being.

Keywords: Antenatal care; Fear; Ghana; Parity; Pregnancy; Risk factors

# Introduction

Fear of childbirth (FoC) is a well-documented psychological phenomenon affecting both maternal and infant health outcomes worldwide (1). It involves persistent feelings of anxiety, worry, and psychological distress related to pregnancy, ~~labor~~labour, and ~~delivery~~childbirth. It can manifest through emotional symptoms such as mood swings, ~~somatization~~somatisation, and cognitive effects like impaired decision-making (2–4). FoC is associated with adverse consequences, including increased risk of maternal depressive symptoms, avoidance behaviours such as preference for caesarean section without medical indication, and undesirable birth outcomes (5–9). A systematic review estimated the prevalence of FoC ranges between 4% and 43%, with an overall pooled prevalence of 14% worldwide (4). Among pregnant women, FoC is influenced by an interplay of sociodemographic, psychosocial, and obstetric factors, including younger age, lower educational attainment, nulliparity, previous obstetric complications, and lack of social support (6,9,10). While nulliparous women often report higher FoC due to inexperience and uncertainty, parous women may develop fears related to adverse past birth experiences (4,6).

Fear of childbirth remains a significant antenatal concern in sub-Saharan Africa, with prevalence studies from nearby countries reporting similar rates (9,11,12). However, direct prevalence and determinants data for Ghana are limited, and existing evidence suggests that cultural and mental health factors – such as anxiety and depression – and obstetric history significantly shape women’s childbirth experiences (11). Furthermore, the specific factors associated with FoC within parity groups remain underexplored. Although previous studies have highlighted the role of parity in shaping FoC (10,12), few have directly assessed parityspecific determinants in the region. Most of these studies either report prevalence separately or treat parity merely as a covariate in multivariable analyses (9,12–14), potentially masking unique risk profiles.

Notably, considering parity only as an adjustment variable may mask unique obstetric and psychosocial routes by which FoC emerges in first-time mothers compared with women with previous birth experience. This is because the factors contributing to FoC may differ across these parity groups, potentially limiting a more nuanced understanding of risk profiles and hindering the design of ~~patient-centered~~patient-centred antenatal counselling strategies. To the best of the authors’ knowledge, no study in Ghana has identified parity-stratified risk factors among nulliparous and parous women. This methodological approach provides an opportunity for identifying within-group risk factors to inform routine antenatal care (ANC) screening in resource-limited primary care settings.

Therefore, a stratified approach is essential to determine whether socio-demographic, obstetric, and psychosocial factors differ by parity, as first-time mothers and those with previous birth experiences may require different antenatal support or interventions. To address these gaps, we aimed to assess the prevalence of FoC and identify parity-specific determinants among ANC attendees in a primary healthcare facility in Kumasi, Ghana. Identifying parity-specific evidence provides guidance for targeted psychosocial care and structured screening within routine ANC to reduce the magnitude of FoC and improve maternal well-being.

**Methods**

# Study design, setting, and population

A facility-based cross-sectional study was conducted among pregnant women attending ANC at HopeXchange Medical Centre in Santasi, Kumasi, between 20^th^ January 2025 and 12^th^ March 2025. Santasi is a suburban area of Kumasi, the capital ~~city~~ of the Ashanti Region in Ghana. HopeXchange Medical Centre is a faith-based facility that provides comprehensive general and ~~specialized~~ specialised medical services. The ANC clinic operates Monday to Friday and is staffed by two doctors and ten midwives. Upon arrival, pregnant women present their ANC booklets and hospital cards at the records unit, after which they are assigned to a midwife for routine consultations. Physician consultations are provided as needed. The facility serves an antenatal population of approximately 5000 women and recorded over 600 deliveries in 2024.

The study population comprised all pregnant women who received ANC at the clinic during the study period. The inclusion criteria were pregnant women of any gestational age who were willing to provide informed consent. The exclusion criteria were pregnant women who were critically ill and required ~~hospitalization~~ hospitalisation at the time of data collection.

The study included all pregnant women irrespective of parity. The study aimed to identify factors associated with FoC in the overall population and to conduct a parity-stratified analysis. This approach enables the identification of both general and parity-specific risk factors to guide targeted ANC counselling and interventions.

# Sample size estimation

The sample size was estimated using the single population proportion formula (13), (𝑠 = (𝑍^2 (𝑃)(1 − 𝑃))/𝐸^2). Where *Z* represents the standard normal value at a 95% confidence interval (1.96), *E* is the error margin (0.05), and *P* is the estimated proportion of pregnant women with FoC (22%) from a previous study (14). This yielded an initial sample size of 264. After adjusting for a 10% non-response rate, the final required sample size was 290 pregnant women.

# Recruitment procedure

Participants were recruited using a simple random sampling technique from a daily attendance list of all pregnant women who had registered for ANC by 10:00 am at the records unit. This time cut-off was operationally realistic, as a feasibility assessment conducted prior to data collection indicated that over 90% of attendees had registered by then. Unique identifiers were assigned, and participants were randomly selected using a lottery method without replacement. Selected pregnant women were contacted individually by research staff to determine their eligibility for inclusion in the study. This process was repeated daily until the estimated sample size was achieved (over 24 days, averaging 12 participants per day).

# Data collection tools and procedures

Data collection was conducted by five research assistants, comprising three midwives and two physician assistants. Prior to data collection, the research assistants participated in a two-day training workshop facilitated by a biostatistician and the principal investigator. The training covered key areas, including quantitative data collection, the consent process, and ~~administering~~ the administration of the data collection tool.

An interviewer-administered questionnaire was used to collect ~~the study data~~data for the study. The investigators developed the questionnaire after reviewing relevant literature (15–21). It was structured into three parts: 1) demographic characteristics, 2) clinical factors, and 3) FoC. The demographic variables included age, marital status, educational level, occupation, religion, head of household, perceived family support, experiencing intimate partner violence (IPV), and history of abuse in the family. These three psychosocial variables were measured using singleitem self-report questions to reduce respondents’ burden and enhance clarity, given their sensitive nature in an ANC setting. Additionally, the clinical factors included ANC booking, whether the decision regarding ANC booking was influenced by another person, whether the current pregnancy was planned, parity, and history of abortion.

FoC, the primary outcome of the study, was measured with the English version of the Wijma Delivery Expectation/Experience Questionnaire (W-DEQ) (22). The W-DEQ is a validated 33item scale designed to measure women’s experiences or expectations of childbirth (22). Each item is rated on a 5-point Likert scale with minimum and maximum scores of 0 and 165, respectively. Although the W-DEQ is a continuous scale, prevalence was defined using a literature-based cut-off for the overall scores: low (≤ 37), moderate (38 – 65), high (66 – 84), or severe (≥ 85) levels of FoC (22). Participants who scored high or severe categories (≥66) were classified as FoC cases (23–25). The W-DEQ has been widely used in other settings to measure FoC among pregnant women (11,20,26,27). In the current study, the scale demonstrated high internal consistency (Cronbach's alpha = 0.975).

The questionnaire was pretested with 20 pregnant women at a similar health facility in another town. The data collected were ~~analyzed~~analysed and used to amend the questionnaire before it was used in the final study. Specifically, the amendment affected the order of the questions, the rewording of ambiguous items, and the inclusion of a preamble to ensure participants understood the questions.

# Statistical analysis and data quality management

Data were exported to Stata version 16 for quality checks, coding, and statistical analysis. Descriptive statistics, such as frequencies and percentages, were used to describe categorical variables, while means with standard deviations and medians with interquartile ranges were used to describe continuous data, where applicable. Parity-stratified comparisons between demographic and clinical characteristics were examined using the chi-square test and independent sample t-test, where necessary. For descriptive purposes, the W-DEQ score, measured as a continuous variable, was ~~categorized~~ categorised into three FoC levels. Severity categories were created and reported descriptively to provide a clinically meaningful context to evaluate the distribution of FoC levels, instead of being used for modelling or subgroup comparison. However, a multivariable linear regression analysis was used to identify factors associated with FoC using continuous W-DEQ scores. We adjusted for both significant (p < 0.2) and clinically relevant variables, based on the existing literature and theoretical effects on FoC, in the multivariable linear regression models. We adopted this approach to ensure that both statistically significant and contextually important variables were considered in the adjusted models. To determine whether the factors associated with FoC differed by parity, stratified multivariable linear regression models were fitted to identify factors that were independently associated with nulliparous and parous women. We set ~~statistical significance at~~ ~~p < 0.05 at~~the statistical significance threshold at p < 0.05, corresponding to a 95% confidence interval. The Variance Inflation Factor (VIF) was used to assess multicollinearity among independent variables for all the multivariable models (overall, nulliparous-specific, and parous-specific). The mean VIFs for the overall, nulliparous-specific, and parous-specific models were 2.44 (range: 1.17 to 4.80), 2.94 (range: 1.48 to 6.86), and 3.02 (range: 1.10 to 6.97), respectively. All mean VIFs were below the conventional threshold of 10, indicating no significant multicollinearity; however, some individual variables had high VIFs. Specifically, in the nulliparous-specific model, tertiary education had a VIF of 6.86, and in the parousspecific model, occupation (formal: 6.97; informal: 6.08) had high VIFs. These variables were retained in the models due to their theoretical significance and ~~because they had little to no~~ ~~effect on the~~ their minimal to no effect on estimates of other variables.

# Ethical approval

This study was conducted in accordance with the ethical principles outlined in the Declaration of Helsinki. The Committee on Human Research, Publications, and Ethics (CHRPE), School of Medicine and Dentistry, Kwame Nkrumah University of Science and Technology (KNUST), Kumasi (CHRPE/AP/025/25) granted ethical approval for this study. Written approval was sought from the study site's management. Before recruitment, all study participants went through a mandatory informed consent process and signed an informed consent form. As part of the consent process, all study participants were provided with information about the study's objectives, significance, and potential risks of participation.

**Results**

# Socio-demographic and clinical characteristics of the study participants stratified by parity status

A total of 282 of the 290 women recruited participated in the study, yielding a response rate of 97.2%. Of this, 35.1% were nulliparous, while the remaining 64.9% were parous. The overall mean age of the study participants was 28 (±5.5) years, with nulliparous women (23.9 ± 3.9 years) being significantly younger than parous women (30.2 ± 5.0 years). Approximately 45.5% of the nulliparous women were single, compared to 8.7% of the parous women. While both groups had relatively ~~higher levels of tertiary education, parous women had slightly higher~~ ~~levels~~high levels of tertiary education, parous women had a slightly higher level (48.1%) than nulliparous women (45.6%). A little over half (50.8%) of parous women had an informal occupation, compared to 24.2% of nulliparous women. The level of family support was similar across the two groups of women (Table 1).

# Table 1: Socio-demographic and clinical characteristics of the study participants stratified by parity

**Characteristics	Parity	Overall,	p-value Nulliparous,	Parous,**

|  | **N = 99 (35.1)** | **N = 183 (64.9)** | **N = 282** |  |
| --- | --- | --- | --- | --- |
| Age (mean ±SD) *^1^* | 23.9 (±3.9) | 30.2 (±5.0) | 28 (±5.5) | **<0.001** |
| **Marital status** |  |  |  | **<0.001** |
| Single | 45 (45.5) | 16 (8.7) | 61 (21.6) |  |
| Married | 38 (38.4) | 126 (68.9) | 164 (58.2) |  |
| Co-habiting | 16 (16.2) | 41 (22.4) | 57 (20.2) |  |
| **Education level** |  |  |  | **0.004** |
| Basic | 9 (9.1) | 41 (22.4) | 50 (17.7) |  |
| Secondary | 45 (45.5) | 54 (29.5) | 99 (35.1) |  |
| Tertiary | 45 (45.6) | 88 (48.1) | 133 (47.2) |  |
| **Occupation** |  |  |  | **<0.001** |
| Unemployed | 39 (39.4) | 9 (4.9) | 48 (17.0) |  |
| Formal | 36 (36.4) | 81 (44.3) | 117 (41.5) |  |
| Informal | 24 (24.2) | 93 (50.8) | 117 (41.5) |  |
| **Religion** |  |  |  | 0.397 |
| Christian | 77 (77.8) | 150 (82.0) | 227 (80.5) |  |
| Muslim | 22 (22.2) | 33 (18.0) | 55 (19.5) |  |
| **Head of household** |  |  |  | **<0.001** |
| Partner | 71 (71.7) | 177 (96.7) | 248 (87.9) |  |
| Self | 9 (9.1) | 6 (3.3) | 15 (5.3) |  |
| Mother/Father | 19 (19.2) | 0 (0.0) | 19 (6.7) |  |
| **Experienced intimate partner violence** |  |  |  | 0.056 |
| Yes | 9 (9.1) | 32 (17.5) | 41 (14.5) |  |
| No | 90 (90.9) | 151 (82.5) | 241 (85.5) |  |
| **History of abuse in the family** |  |  |  | 0.585 |
| Yes | 22 (22.2) | 46 (25.1) | 68 (24.1) |  |
| No | 77 (77.8) | 137 (74.9) | 214 (75.9) |  |
| **ANC booking (weeks)** |  |  |  | 0.384 |
| Early (1 – 12) | 71 (71.2) | 122 (66.7) | 193 (68.4) |  |
| Late (13+) | 28 (28.3) | 61 (33.3) | 89 (31.6) |  |
| **ANC booking depends on partner’s decision** |  |  |  | 0.260 |
| Yes | 21 (21.2) | 29 (15.9) | 50 (17.7) |  |
| No | 78 (78.8) | 154 (84.2) | 232 (82.3) |  |
| **Planned the current pregnancy** |  |  |  | 0.493 |
| Yes | 45 (45.5) | 91 (49.7) | 136 (48.2) |  |
| No | 54 (54.6) | 92 (50.3) | 146 (51.8) |  |

| **History of abortion** |  |  |  | **0.001** |
| --- | --- | --- | --- | --- |
| Yes | 36 (36.4) | 34 (18.6) | 70 (24.8) |  |
| No | 63 (63.6) | 149 (81.4) | 212 (75.2) |  |
| **Family’s support** |  |  |  | 0.746 |
| Poor | 25 (25.3) | 40 (21.9) | 65 (23.1) |  |
| Moderate | 33 (33.3) | 68 (37.2) | 101 (35.8) |  |
| Strong | 41 (41.4) | 75 (41.0) | 116 (41.1) |  |

233	*^1^*Continuous variables reported as mean (±SD); all others as n (%)

234

## 235 **Prevalence of FoC among study participants**

236 Overall, the prevalence of FoC among the study participants was 22.0% (≥66) (Figure 1). The 237 median W-DEQ score among the study participants was 41 (interquartile range [IQR]: 33 – 238 61). When stratified by parity, nulliparous women (median: 46.0, IQR: 37.0–66.0) reported 239 significantly higher median W-DEQ scores than parous women (median: 39.0, IQR: 32.0– 240 58.5). Regarding the severity of FoC, nulliparous women reported significantly higher levels 241 of FoC compared to parous women, with a greater proportion experiencing severe fears (16.2% 242 vs 10.4%) (Table 2).

243

244	**Table 2: Prevalence of FoC among study participants stratified by parity**

# Characteristics Nulliparous, Parous,

**Parity**

**Overall,**

**p**

**-**

**value**

|  | | **N = 99** | **N = 183** | **N = 282** |  |
| --- | --- | --- | --- | --- | --- |
| **W-DEQ** Median (IQR) | | 46.0 (37.0–66.0) | 39.0 (32.0–58.5) | (33 – 61) | **0.004** |
| **Levels of FoC, n (%)** Low | | (28.3) | (45.4) | (39.4) | **0.039** |
| Moderate | | (45.5) | (35.0) | (38.7) |  |
| High | | (10.1) | (9.3) | (9.6) |  |
| Severe | | (16.2) | (10.4) | (12.4) |  |
| IQR: Interquartile range |  |  |  |  |  |

## 245 246

## 247

Figure: Prevalence of FoC among study participants

# Factors associated with FoC among study participants

In the unadjusted model, factors such as age (β**:** -1.25, 95%CI: -1.74, 0.77), being married (β**:** -27.13, 95%CI: -33.27, -20.98), having formal employment (β**:** -25.34, 95%CI: -32.72, -17.95), experiencing intimate partner violence (β**:** 29.42, 95% CI: 22.26, 36.57), booking ANC late (β**:** 20.82, 95%CI: 15.32, 26.33), planning current pregnancy (β**:** 25.71, 95%CI: 20.99, 30.43) and strong family’s support (β**:** -36.27, 95%CI: -42.18, -30.36) were significantly associated with FoC. In the adjusted model, factors such as being in a cohabiting relationship (adj. β**:** -8.74, 95%CI: -15.23, -2.25), having secondary (adj. β**:** -7.58, 95%CI: -13.58, -1.58) and tertiary education (Adj. β**:** -9.44, 95%CI: -17.65, -1.23), having informal occupation (adj. β**:** -7.88, 95%CI: -14.83, -0.92), having history of abuse in the family (adj. β**:** 10.38, 95%CI: 4.47, 16.28), late ANC booking (adj. β**:** 6.32, 95%CI: 1.67, 10.96), not planning the current pregnancy (adj. β**:** 8.84, 95%CI: 3.72, 13.96) and having moderate (adj. β**:** -13.10, 95%CI: 18.70, -7.50): and strong (adj. β**:** -19.87, 95%CI: -26.53, -13.22) family’s support remained significantly associated with FoC after adjusting for other covariates (Table 3).

In the parity-stratified analysis, among nulliparous women, factors such as having a history of family abuse (adj. β**:** 16.32, 95%CI: 2.50, 30.14) and late ANC booking (adj. β**:** 13.13, 95%CI: 1.43, 24.84) were significantly associated with FoC. Among parous women, factors such as having secondary education (adj. β**:** -7.63, 95%CI: -14.88, -0.37), experiencing intimate partner violence (adj. β**:** 10.87, 95%CI: 0.69, 21.04), not planning current pregnancy (adj. β**:** 8.38, 95%CI: 2.51, 14.26), and having moderate (adj. β**:** -23.10 (-31.91, -14.30) and strong (adj. β**:**

-27.94, 95%CI: -38.77, -17.11) family support was significantly associated with FoC (Table 4).

| **Characteristics** | **β (95% CI)** | **p-value** | **Adj. β (95% CI)** | **p-value** |
| --- | --- | --- | --- | --- |
| Age | -1.25 (-1.74, 0.77) | **<0.001** | -0.35 (-0.86, 0.16) | 0.182 |
| Parity |  |  |  |  |
| Nulliparous | 1.00 |  | 1.00 |  |
| Parous | -7.78 (-13.80, -1.75) | **0.012** | -3.35 (-8.81, 2.11) | 0.228 |
| **Marital status** |  |  |  |  |
| Single | 1.00 |  | 1.00 |  |
| Married | -27.13 (-33.27, -20.98) | **<0.001** | 3.35 (-10.03, 3.33) | 0.324 |
| Co-habiting | -8.02 (-15.56, -0.47) | **0.038** | -8.74 (-15.23, -2.25) | **0.009** |
| **Education level** |  |  |  |  |
| Basic | 1.00 |  | 1.00 |  |
| Secondary | -11.94 (-19.08, -4.80) | **0.001** | -7.58 (-13.58, -1.58) | **0.014** |
| Tertiary | -29.65 (-36.48, -22.82) | **<0.001** | -9.44 (-17.65, -1.23) | **0.024** |
| **Occupation** |  |  |  |  |
| Unemployed | 1.00 |  | 1.00 |  |
| Formal | -25.34 (-32.72, -17.95) | **<0.001** | -5.65 (-12.61, 1.31) | 0.111 |
| Informal | -9.89 (-17.28, -2.51) | **0.009** | -7.88 (-14.83, -0.92) | **0.027** |
| **Religion** |  |  |  |  |
| Christian | 1.00 |  | 1.00 |  |
| Muslim | 4.39 (-2.66, 11.44) | 0.221 | -3.50 (-8.73, 1.74) | 0.190 |
| **Experienced intimate partner violence** |  |  |  |  |
| No | 1.00 |  | 1.00 |  |
| Yes | 29.42 (22.26, 36.57) | **<0.001** | 6.59 (-0.64, 13.82) | 0.074 |
| **History of abuse in the family** |  |  |  |  |
| No | 1.00 |  | 1.00 |  |
| Yes | 26.51 (20.75, 32.27) | **<0.001** | 10.38 (4.47, 16.28) | **0.001** |
| **ANC booking (weeks)** |  |  |  |  |
| Early (1 – 12 | 1.00 |  | 1.00 |  |
| Late (13+) | 20.82 (15.32, 26.33) | **<0.001** | 6.32 (1.67, 10.96) | **0.008** |
| **Planned the current pregnancy** |  |  |  |  |
| Yes | 1.00 |  | 1.00 |  |
| No | 25.71 (20.99, 30.43) | **<0.001** | 8.84 (3.72, 13.96) | **0.001** |
| **History of abortion** |  |  |  |  |
| No | 1.00 |  | 1.00 |  |
| Yes | 3.37 (-3.11, 9.84) | 0.307 | -2.14 (-6.82, 2.54) | 0.369 |
| **Family’s support** |  |  |  |  |
| Poor | 1.00 |  | 1.00 |  |
| Moderate | -20.44 (-26.50, -14.38) | **<0.001** | -13.10 (-18.70, -7.50) | **<0.001** |
| Strong | -36.27 (-42.18, -30.36) | **<0.001** | -19.87 (-26.53, -13.22) | **<0.001** |

# Table 3: Linear regression analysis of factors associated with FoC among study participants

275 β = unstandardized regression coefficient; CI = confidence interval; 1:00 = reference category;
276 Adj. β: adjusted regression coefficient
277 278

279 **Table 4: Stratified multivariable linear regression analysis of factors associated with FoC** 280 **by parity status**

# Characteristics Nulliparous Parous

**Adj. β (95% CI)	p-value	Adj. β (95% CI)	p-value**

Age	-0.69 (-3.00, 1.62)	0.556	-0.10 (-0.62, 0.42)	0.714

# Marital status

Single	1.00	1.00

Married	-8.06 (-18.61, 2.49)	0.133	-0.96 (-10.94, 9.02)	0.850

Co-habiting	-4.34 (-14.40, 5.71)	0.393	-7.36 (-17.72, 3.01)	0.163

# Education level

Basic	1.00	1.00

Secondary	-12.16 (-32.65, 8.33)	0.241	-7.63 (-14.88, -0.37)	**0.039**

Tertiary	-16.91 (-40.83, 7.01)	0.164	-8.54 (-20.72, 3.17)	0.152

# Occupation

Unemployed 1.00 1.00 Formal -1.24 (-10.59, 8.10) 0.792 -4.41 (-13.14, 4.33) 0.321

Informal	-3.27 (-13.79, 7.25)	0.538	-7.48 (-15.24, 0.29)	0.059

# Experienced intimate partner violence

No	1.00	1.00

Yes	4.83 (-14.56, 24.22)	0.622	10.87 (0.69, 21.04)	**0.037**

# History of abuse in the family

No	1.00	1.00

Yes	16.32 (2.50, 30.14)	**0.021**	6.22 (-1.67, 14.11)	0.121

# ANC booking (weeks)

Early (1 – 12)	1.00	1.00

Late (13+)	13.13 (1.43, 24.84)	**0.028**	3.20 (-2.17, 8.58)	0.241

# Planned the current pregnancy

Yes	1.00	1.00

No	-3.69 (-15.07, 7.69)	0.521	8.38 (2.51, 14.26)	**0.005**

# History of abortion

No	1.00	1.00

Yes	-4.05 (-12.36, 4.26)	0.335	4.56 (-1.75, 10.87)	0.155

# Family’s support

Poor	1.00	1.00

Moderate	5.06 (-6.32, 16.44)	0.379	-23.10 (-31.91, -14.30)	**<0.001**

## Strong -9.06 (-19.52, 1.39) 0.088 -27.94 (-38.77, -17.11) **<0.001**

281 β = unstandardized regression coefficient; CI = confidence interval; 1:00 = reference category;
282 Adj. β: adjusted regression coefficient
283 284	285

# Discussions

This study sought to examine the prevalence and determinants of FoC among pregnant women attending ANC in a primary healthcare facility in Kumasi, Ghana, with a specific focus on parity (i.e., differences between nulliparous and parous women). We found that FoC is relatively common, with nulliparous women reporting higher fears than parous women, and approximately 22% experiencing high to severe FoC. Our findings underpin a critical need to screen for FoC during routine ANC, especially in settings with limited psychological support.

We found that FoC was substantial in this cohort. Our finding that approximately 22% of the participants experienced high to severe FoC (W-DEQ scores ≥66) is consistent with studies from Malawi (20%) and Australia (24%) (28,29), but lower than the rates reported in Kenya (30.1%), China (35.8%), and the Netherlands (33.9%) (10,24,30). Additionally, the median WDEQ score in our study was 41, comparable to a similar survey in Malawi (mean: 47), but lower than what has been documented in high-income countries (21,31,32). This variability may reflect differences in population characteristics, healthcare systems, cultural perceptions of childbirth, availability of psychosocial support, and methodological disparities, including skewness in the distribution of W-DEQ scores. This suggests that the FoC experience is not uniform and varies across populations.

Nulliparous women experienced significantly higher fears than parous women, consistent with a previous study conducted in Finland and other low- and middle-income countries (33–35). A plausible explanation could be that first-time mothers lack previous birth experience, leading to uncertainty and perceived higher pain risk. In contrast, parous women, having experienced childbirth, may develop coping mechanisms that reduce the fear.

Consistent with previous research, our findings demonstrate that nulliparous women were more likely to be younger, single, and experiencing childbirth for the first time (36). These characteristics have been consistently associated with increased FoC. For instance, Duong *et al.* found that nulliparous women tend to have greater FoC due to the unpredictability and novelty of the experience (37). Additionally, the association between a history of family abuse and higher FoC observed in our nulliparous subgroup is supported by earlier studies by Goldstein *et al.* and Van Parys *et al*. (38,39). Addressing abuse-related trauma through antenatal counselling represents a critical intervention point to mitigate FoC. Another key finding in nulliparous women was the association between late ANC booking and increased FoC. Late ANC booking may limit access to early interventions, including education and support that could alleviate these fears. Research suggests that nulliparous women who engage in timely ANC are better prepared for childbirth, which subsequently helps to reduce their fear (40).

Among parous women, our findings revealed that higher educational attainment, particularly completion of secondary education, is associated with lower levels of FoC than a basic education, consistent with studies from Iran and Turkey (41,42). This trend suggests that education enhances knowledge, confidence, and facilitates communication with healthcare providers (43–45). The observed association between IPV, unplanned pregnancy, and FoC among parous women is well-established (46–49), with significant implications for maternal health. Our study contributes to this body of knowledge by demonstrating that this relationship persists even after adjusting for other factors, underscoring the need for psychosocial support and reproductive counselling.

Another critical factor was the inverse association between family support and FoC, underscoring the broader literature emphasis~~z~~ing the protective role of social support on maternal anxiety and childbirth-related stress (50,51). Women with strong support systems tend to feel more empowered, informed, and emotionally secure, which often results in reduced levels of fear and anxiety (52). This finding ~~underpins the importance of social networks in~~ ~~improving childbirth experiences in Ghana and advances knowledge of the psychosocial~~ ~~factors that influence FoC in a limited-resource setting~~underscores the importance of social networks in improving childbirth experiences in Ghana and advances understanding of the psychosocial factors that influence FoC in resource-limited settings. The protective role of family support increases the understanding of how social variables shape FoC. This highlights the importance of integrating family-oriented approaches into ANC services to reduce FoC and improve maternal well-being*.* This finding is particularly relevant in the Ghanaian context, where extended family structures and community networks play a crucial role in shaping women’s health experiences. Encouraging family involvement in ANC and advocating for supportive environments may be essential to reducing FoC.

# Implications for Practice and Research

The findings of this study have important implications for maternal healthcare delivery in Ghana and similar low-resource settings. Our findings highlight the need for parity-specific interventions. Specifically, nulliparous women may benefit from structured antenatal education and early ANC booking, while psychosocial counselling, IPV screening, and family support may be helpful in parous mothers.

Our findings suggest that, in addition to routine ANC programs that screen for FoC, they should also integrate ~~patient-centered~~patient-centred strategies that account for the unique risk profiles within each parity group. ~~There is a need for healthcare managers~~ Healthcare managers need to train professionals to identify at-risk populations, provide ~~patient-centered~~patient-centred education, and involve family members when necessary to promote social support. Incorporating FoC screening tools into routine ANC guidelines could help identify vulnerable women who could benefit from additional psychological or social support strategies, thereby enhancing maternal satisfaction and childbirth preparedness.

Future research should investigate the effectiveness of culturally tailored FoC-reduction interventions, including peer support groups, mobile-based health education tools, and traumainformed care. Longitudinal studies are also needed to track changes in FoC across pregnancy and postpartum, and its influence on maternal outcomes.

# Strengths and Limitations of the Study

A key strength of this study is its stratified analytical approach, which enabled an in-depth comparison of FoC determinants across parity groups. This methodological decision enhances the utility of the findings for developing tailored interventions. The study also employed the W-DEQ, a widely validated tool for assessing FoC, thereby strengthening the study's internal validity.

However, several limitations must be noted. First, the study was conducted in a single primary healthcare facility, which may limit the generalizability of the findings to other regions or facility types in Ghana. Second, some variables, such as IPV, family abuse, and family support, were measured using a single-item self-report, which may reduce measurement breadth and precision that could have been examined with multiple items. Again, participants' gestational age could have influenced their FoC. However, gestational age was not collected as a study variable and therefore could not be included in the model. Consequently, we acknowledge it as a limitation of the study. Finally, although the sample size was adequate for the primary analyses, it may not have been powered to detect minor effects or subgroup interactions.

Despite these limitations, the study provides valuable insights into the psychological experiences of pregnant women in a resource-constrained setting and identifies clear intervention points to address FoC.

# Conclusion

The current study highlights that FoC is a significant psychological burden among pregnant women in Kumasi, Ghana, with approximately one in five women reporting high or severe levels of fear. The determinants of FoC varied by parity: nulliparous women were more affected by abuse and late ANC booking, while parous women’s fear was influenced by education level, intimate partner violence, unplanned pregnancy, and perceived family support.

Our findings contribute to understanding FoC in low-resource settings by identifying parityspecific risk profiles that have previously gone unexplored in Ghana. These findings underscore the need for parity-specific strategies in addressing FoC during ANC. Routine screening for FoC, early ANC engagement, psychosocial support, and family-inclusive care models could collectively reduce childbirth-related fear and promote better maternal health outcomes. Future research should explore these strategies using longitudinal, interventional, and qualitative designs to guide policy and practice in Ghana and similar settings.

# Acknowledgement

For their important contributions to this study, we would especially like to thank the ~~HopeXchange Medical Centre's management and employees~~management and staff of the HopeXchange Medical Centre, the ~~study~~ participants, and our research assistants.

# Author Contributions

~~Conceptualization~~Conceptualisation: JOA, DAO & EAB

Data curation: JOA, DAO, MA, EK, FKK, & JKK

Formal analysis: DAO, MA, EK, & JKK

Methodology: DAO, EAB, JOA, MK, OKOA, & FKK

Project administration: JOA, DAO, MK, OKOA, & EAB

Supervision: EAB

Validation: DAO, MA, EK, MK, JOA, OKOA, FKK, EAB & JKK

Writing – original draft: DAO, MA, EK, & JKK

Writing – review and editing: JOA, DAO, MK, OKOA, JKK, MA, EK, FKK & EAB

**Competing interest**

The authors have declared that no competing interests exist.

**Funding**

The authors received no specific funding for this work.

# References

1. Chen C, Hussein SZB, Nasri NWM, Yao J, Qin Y, Zhao Z, et al. Fear of childbirth among pregnant women: A concept analysis. J Adv Nurs. 2024;80(11):4476–87.
2. Wigert H, Nilsson C, Dencker A, Begley C, Jangsten E, Sparud-Lundin C, et al. Women’s experiences of fear of childbirth: a metasynthesis of qualitative studies. Int J Qual Stud Health Well-being. 2020;15(1).
3. Hofberg K, Ward MR. Fear of pregnancy and childbirth. Postgrad Med J. 2003;79(935):505–10.
4. O’Connell MA, Leahy-Warren P, Khashan AS, Kenny LC, O’Neill SM. Worldwide prevalence of tocophobia in pregnant women: systematic review and meta-analysis. Acta Obstet Gynecol Scand. 2017;96(8):907–20.
5. Li B, Liu T, Ma D, Sun J, Liu J. Association of fear of childbirth and postpartum depression with perceived partner response during pregnancy. BMC Pregnancy Childbirth. 2025;25(1):1–9.
6. Ali MH, Seif SA, Kibusi SM. The Influence of Fear During Pregnancy, Labour and Delivery on Birth Outcome Among Post-Delivery Women: A Case Control Study in Zanzibar. East African Heal Res J. 2022;6(2):147–54.
7. Elgzar WT, Alshahrani MS, Ibrahim HAF. Mode of delivery preferences: the role of childbirth fear among nulliparous women. Front Psychol. 2023;14(November):1–10.
8. Rúger-Navarrete, A., Vázquez-Lara, J.M., Antúnez-Calvente, I., Rodríguez-Díaz, L., Riesco-González, F.J., Palomo-Gómez, R., Gómez-Salgado, J. and Fernández-Carrasco, FJ. Antenatal fear of childbirth as a risk factor for a bad childbirth experience. Healthcare. 2023;11(3):1–13.
9. Massae AF, Larsson M, Pembe AB, Mbekenga C, Svanberg AS. Patterns and predictors of fear of childbirth and depressive symptoms over time in a cohort of women in the Pwani region, Tanzania. PLoS One. 2022;17(11 November).
10. Onchonga D, MoghaddamHosseini V, Keraka M, Várnagy Á. Prevalence of fear of childbirth in a sample of gravida women in Kenya. Sex Reprod Healthc. 2020;24:100510.
11. Khwepeya M, Lee GT, Chen SR, Kuo SY. Childbirth fear and related factors among pregnant and postpartum women in Malawi. BMC Pregnancy Childbirth.

2018;18(1):391.

12. Berhanu RD, Abathun AD, Negessa EH, Amosa LG. The magnitude and associated factors of childbirth fear among pregnant women attending antenatal care at public hospitals in Ethiopia: a cross-sectional study. BMC Pregnancy Childbirth. 2022;22(1).
13. Charan J, Biswas T. How to calculate sample size for different study designs in medical research? Vol. 35, Indian Journal of Psychological Medicine. 2013. p. 121–6.
14. Hildingsson I, Haines H, Karlström A, Nystedt A. Presence and process of fear of birth during pregnancy — Findings from a longitudinal cohort study. Women and Birth. 2017;(2016):2–7.
15. Phunyammalee M, Buayaem T, Boriboonhirunsarn D. Fear of childbirth and associated factors among low-risk pregnant women. J Obstet Gynaecol (Lahore). 2019;39(6):763–

7.

16. O’Connell MA, Leahy‐Warren P, Kenny LC, O’Neill SM, Khashan AS. The prevalence and risk factors of fear of childbirth among pregnant women: A cross‐sectional study in Ireland. Acta Obstet Gynecol Scand. 2019;98(8):1014–23.
17. Dencker A, Nilsson C, Begley C, Jangsten E, Mollberg M, Patel H, et al. Causes and outcomes in studies of fear of childbirth: A systematic review. Women and Birth. 2019;32(2):99–111.
18. Räisänen S, Lehto SM, Nielsen HS, Gissler M, Kramer MR, Heinonen S. Fear of childbirth in nulliparous and multiparous women: a population‐based analysis of all singleton births in Finland in 1997–2010. BJOG An Int J Obstet Gynaecol. 2014;121(8):965–70.
19. He D, Zhang J, Wang G, Huang Y, Li N, Zhu M, et al. Prevalence and factors associated with fear of childbirth in late pregnancy: a cross-sectional study. Front Public Heal.

2025;13:1589568.

20. Dal Moro APM, Soecki G, de Fraga FS, Petterle RR, Rückl SZ. Fear of childbirth:

prevalence and associated factors in pregnant women of a maternity hospital in southern Brazil. BMC Pregnancy Childbirth. 2023;23(1):1–12.

21. Lukasse M, Schei B, El R. Prevalence and associated factors of fear of childbirth in six European countries. Sex Reprod Healthc. 2014;5(3):99–106.
22. Wjma K. Psychometric aspects of the W-DEQ ; a new questionnaire for the measurement of fear of childbirth. J Psychosom Obstet Gynecol. 1998;19(2):84–97.
23. Massae AF, Larsson M, Leshabari S, Mbekenga C, Pembe AB, Svanberg AS. Predictors of fear of childbirth and depressive symptoms among pregnant women: a cross-sectional survey in Pwani region, Tanzania. BMC Pregnancy Childbirth. 2021;21(1):1–13.
24. Fang W, Jiang H, Chen A. The impact of fear of childbirth on mode of delivery, postpartum mental health and breastfeeding: A prospective cohort study in Shanghai , China. J Affect Disord. 2024;347(May 2023):183–91.
25. Jing Q, Jie J, Ke X, Liu Y, Xiumin D, Xiuchuan L, et al. Developmental trajectories of maternal fear of childbirth at different times: a prospective longitudinal study. BMC ~~Womens~~ Women's Health. 2025;25:325.
26. Adams SS, Eberhard-Gran M, Eskild A. Fear of childbirth and duration of labour: A study of 2206 women with intended vaginal delivery. BJOG An Int J Obstet Gynaecol.

2012;119(10):1238–46.

27. Alehagen S, Wijma B, Wijma K. Fear of childbirth before, during, and after childbirth. Acta Obstet Gynecol Scand. 2006;85(1):56–62.
28. Khwepeya M, Lee GT, Chen SRSR, Kuo SYSY. Childbirth fear and related factors among pregnant and postpartum women in Malawi. BMC Pregnancy Childbirth. 2018;18(1):391.
29. Toohill J, Fenwick J, Gamble J, Creedy DK. Prevalence of childbirth fear in an Australian sample of pregnant women. BMC Pregnancy Childbirth. 2014;14:275.
30. Veringa-Skiba IK, Bruin EI De, Mooren B, Steensel FJA Van, Bögels SM. Can a simple assessment of fear of childbirth in pregnant women predict requests and use of nonurgent obstetric interventions during labour?. Midwifery. 2021;97(May 2020):102969.
31. Hall WA, Hauck YL, Carty EM, Hutton EK, Fenwick J, Stoll K. Childbirth fear, anxiety, fatigue, and sleep deprivation in pregnant women. J Obstet Gynecol Neonatal Nurs. 2009;38(5):567–76.
32. Fenwick J, Gamble J, Nathan E, Bayes S, Hauck Y. Pre-and postpartum levels of childbirth fear and the relationship to birth outcomes in a cohort of Australian women. J Clin Nurs. 2009;18(5):667–77.
33. Oladele OC, Akinwaare MO, Ogbeye GB, Oluseye O. Childbirth fear and birth-related mindset of first-time mothers who registered for antenatal care in public hospitals in Nigeria. Int J Childbirth. 2024;14(4).
34. Mandar O, Idrees MB, Ahmed A, ALhabardi N, Hassan B, Adam I. Prevalence and associated factors of fear for childbirth among pregnant women in eastern Sudan. J Reprod Infant Psychol. 2023;41:319–29.
35. Rouhe H, Salmela-Aro K, Halmesmäki E, Saisto T. Fear of childbirth according to parity, gestational age, and obstetric history. BJOG An Int J Obstet Gynaecol. 2009;116(1):67–73.
36. Geidam A. Pregnancy outcome among nulliparous women at the University of Maiduguri Teaching Hospital, North Eastern Nigeria: a retrospective cohort study. Br J Med Med Res. 2014;4:3893–901.
37. Duong HT, Hoyt AT, Carmichael SL, Gilboa SM, Canfield MA, Case A, et al. Is maternal parity an independent risk factor for birth defects? Birth Defects Res. 2012;94:230–6.
38. Goldstein BL, Briggs-Gowan MJ, Grasso DJ. The effects of intimate partner violence and a history of childhood abuse on mental health and stress during pregnancy. J Fam Violence. 2021;36(3):337–46.
39. Van Parys AS, Deschepper E, Michielsen K, Galle A, Roelens K, Temmerman M, et al. Intimate partner violence and psychosocial health, a cross-sectional study in a pregnant population. BMC Pregnancy Childbirth. 2015;15:278.
40. Bektas Pardes B, Guvenc G. The effects of antenatal education and telephone counseling on childbirth fear of nulliparous women and their attitudes toward childbirth: a randomized controlled trial. Rev Assoc Med Bras. 2025;71:e20241147.
41. Soysal C, Ulaş Ö, Işıkalan MM, Bıyık İ, Taşçı Y, Keskin N. The changes in fear of childbirth in pregnancy during and before the COVID-19 pandemic. Sci Rep. 2024;14:11067.
42. Barat S, Kordinejad B, Faramarzi M, Khafri S, Bouzari Z, Ebrahim E. Prevalence of fear of childbirth and its effective factors in pregnant women in Babol, Iran (2019-2020): a cross-sectional study. Int J Women’s Heal Reprod Sci. 2023;11(1):25–32.
43. Ketema DB, Leshargie CT, Kibret GD, Assemie MA, Petrucka P, Alebel A. Effects of maternal education on birth preparedness and complication readiness among Ethiopian pregnant women: A systematic review and meta-analysis. BMC Pregnancy Childbirth. 2020;20(1):1–9.
44. Amponsah E, Fusheini A, Adam A. Influence of information, education and communication on prenatal and skilled delivery in the Tano North District, Ghana: A cross-sectional study. Heliyon. 2021;7(6):e07245.
45. Greenaway ES, Leon J, Baker DP. Understanding the association between maternal education and use of health services in Ghana: Exploring the role of health knowledge. J Biosoc Sci. 2012;44(6):733–47.
46. Adu-Bonsaffoh K, Tamma E, Seffah JD. Prevalence and determinants of unintended pregnancy among women receiving antenatal care services: a facility-based crosssectional study in Ghana. Glob J Health Sci. 2021;13(9):100.
47. Alain DC, Nidelle TA, Evrard KN, Franck KF, Armand NNW, Sadrack A, et al. Domestic violence during pregnancy and risk of obstetrical complications. African Clin Res Innov. 2024;24:142–55.
48. Eliason S, Baiden F, Yankey BA, Awusabo-Asare K. Determinants of unintended pregnancies in rural Ghana. BMC Pregnancy and Childbirth. 2014;14(1). 261&partnerID=40&md5=32f6a261400d8df1c8fb50cd1fad5f96
49. Oğurlu M, Erbil N. The effect of intimate partner violence on fear of childbirth among pregnant women. J Interpers Violence. 2023;38(3–4):3737–55.
50. Pinar SE, Aksoy OD, Cesur B, Bilgic D, Daglar G, Guler E. Investigation of the relationship between fear of childbirth and social supports of pregnant women in the third trimester in Turkey. Clin Exp Obstet Gynecol. 2017;44:767–71.
564 51.	Baheiraei A, Mirghafourvand M, Mohammadi E, Charandabi SMA, Nedjat S. Social
565 support for women of reproductive age and its predictors: a population-based study.
566 BMC Women's Health. 2012;12:30.
567 52.	Acoba EF. Social support and mental health: the mediating role of perceived stress.
568 Front Psychol. 2024;15(February):1–12.

569

## 570 **Supporting information**

571	Datasets used for the analysis.

Response to Reviewers

# AUTHORS' RESPONSES TO REVIEWER’S COMMENTS

| **Reviewer’s comments** | **Authors’ response** |
| --- | --- |
| **Reviewer 4** | |
| The authors have thoroughly addressed the previous reviewers’ comments and successfully resolved the technical and methodological issues in the revised manuscript. However some minor typographical errors might have introduced or missed during the revision process and hence suggesting some minor corrections. | We are grateful to you, the other reviewer and the editor for ensuring that the quality and rigour of our manuscript is improved. We strongly believe that your comments have been extremely helpful in improving the quality of the manuscript, and we appreciate them. |
| **Page 2, Line 34:** using hyphen (-) between identifying and parity-specific determinants “identifying-parity-specific determinants” **Suggested correction:** identifying parityspecific determinants | We are grateful to the reviewer for drawing our attention to this. We statement in reference has been revised and it now reads as;    *Previous studies on Fear of Childbirth (FoC) across sub-Saharan Africa have typically reported prevalence separately by parity or treated it as a covariate in multivariable analyses, with little attention given to identifying parityspecific determinants* |
| **Line 34:** Misspelling of “Howerver” | This has been corrected in the revised manuscript, and it now reads as;    *However, first-time mothers and those with previous birth experiences may require different forms of antenatal support* |
| **Page 3, Line 67:** worry, and psychological distress related to pregnancy, labor, and delivery.  **Suggested correction:** Please replace the word “delivery” with “childbirth” to more accurately reflect the clinical context discussed in this section. | We are grateful to the reviewer for this comment, and in line with that, the statement in reference has been revised to read as;    *It involves persistent feelings of anxiety, worry, and psychological distress related to pregnancy, labour, and childbirth* |
| **Page 6, Line 170:** spacing between the word “analyzedand” | This has been done as recommended. It now reads as;    *The data collected were analysed and used to amend the questionnaire before it was used in the final study.* |
