# Supplementary material for: Fear of childbirth among antenatal care attendees in a primary care setting in Kumasi, Ghana: Prevalence and parity-stratified determinants
Source: PLoS One. 2026 Sep 21;21(9):e0358457. doi: 10.1371/journal.pone.0358457 (PMC13592637; doi:10.1371/journal.pone.0358457)
Supplement: Supplementary file 11 [file pone.0358457.s011.docx]

**Reviewer comments**

Line 68-69

emotional symptoms such as mood swings and somatisation, as well as cognitive effects,

69 including impaired decision-making (2–4).

Suggestion

emotional symptoms (e.g., mood swings, somatisation) and cognitive effects including impaired decision-making (2–4).

Line 117

Complete the description of HopeXchange Medical Centre. 600 deliveries represent 12% of the population and around 50 per month.

How many women were received during ANC1 in 2024.? How many cases for curative consultation?

This Private not-for-profit surely attracts women outside the estimated population.

Are there some reasons why this health facility was selected?

Line 131 … a 10% non-response rate, the final required sample size was 290 pregnant women.

Replace with :

…a 10% non-response rate, the final required sample size was 290 pregnant women (Figure 1).

Ethical approval

Do some researchers work in the HopeXchange Medical Centre facility as health care provider? If yes, the authors should indicate.

**Page 10**, Figure Prevalence of FoC among study participants

- Indicate the number of the Figure (e.g **Figure 1**)
- Cite in t
- As there are only two items, Remove the legend and insert directly Cases and Non-cases directly in the Figure

Page 29

Family support was inversely associated with FoC, consistent with evidence highlighting the protective role of social support against maternal anxiety and childbirth-related stress (50,51). Women with strong support systems tend to feel more empowered, informed, and emotionally secure, which may reduce fears and anxiety (52). This finding underscores the importance of psychosocial supports in shaping childbirth experiences and suggests that integrating family oriented approaches into ANC services could help reduce FoC and improve maternal…

Comment

This means that by providing family support, women will be more empowered, informed, and emotionally secure, which may reduce fears and anxiety.

This is Iin contradiction with the results showed that women with family support have more FoC.

Maybe, there is a bias that family support is mostly provided to women who have already FoC. Therefore, family support will not be considered as an associated factor.

Could the authors explain and discuss more this factor?

Page 17

Line 10369 : generalizability

This is US and the authors used the British English. Therefore, the spelling should be **generalisability**
