# Supplementary material for: Fear of childbirth among antenatal care attendees in a primary care setting in Kumasi, Ghana: Prevalence and parity-stratified determinants
Source: PLoS One. 2026 Sep 21;21(9):e0358457. doi: 10.1371/journal.pone.0358457 (PMC13592637; doi:10.1371/journal.pone.0358457)
Supplement: Peer review history

# Peer review: Decision Letter 0

**DOI:** 10.1371/journal.pone.0358457.r001

**Authors:** Ilse Bloom

28 Nov 2025

Fear of childbirth in Ghana: Prevalence and within parity-specific determinants among antenatal care attendees in a health facility in Kumasi, Ghana

PLOS ONE

Dear Dr. Opoku,

Thank you for submitting your manuscript to PLOS ONE. After careful consideration, we feel that it has merit but does not fully meet PLOS ONE’s publication criteria as it currently stands. Therefore, we invite you to submit a revised version of the manuscript that addresses the points raised during the review process.

Please submit your revised manuscript by Jan 11 2026 11:59PM. If you will need more time than this to complete your revisions, please reply to this message or contact the journal office at plosone@plos.org. When you're ready to submit your revision, log on to https://www.editorialmanager.com/pone/ and select the 'Submissions Needing Revision' folder to locate your manuscript file.

- A rebuttal letter that responds to each point raised by the academic editor and reviewer(s). You should upload this letter as a separate file labeled 'Response to Reviewers'.
- A marked-up copy of your manuscript that highlights changes made to the original version. You should upload this as a separate file labeled 'Revised Manuscript with Track Changes'.
- An unmarked version of your revised paper without tracked changes. You should upload this as a separate file labeled 'Manuscript'.

If you would like to make changes to your financial disclosure, please include your updated statement in your cover letter. Guidelines for resubmitting your figure files are available below the reviewer comments at the end of this letter.

If applicable, we recommend that you deposit your laboratory protocols in protocols.io to enhance the reproducibility of your results. Protocols.io assigns your protocol its own identifier (DOI) so that it can be cited independently in the future. For instructions see: https://journals.plos.org/plosone/s/submission-guidelines#loc-laboratory-protocols. Additionally, PLOS ONE offers an option for publishing peer-reviewed Lab Protocol articles, which describe protocols hosted on protocols.io. Read more information on sharing protocols at https://plos.org/protocols?utm_medium=editorial-email&utm_source=authorletters&utm_campaign=protocols.

We look forward to receiving your revised manuscript.

Kind regards,

Ilse Bloom

Staff Editor

PLOS ONE

Journal Requirements:

When submitting your revision, we need you to address these additional requirements.

1.Please ensure that your manuscript meets PLOS ONE's style requirements, including those for file naming. The PLOS ONE style templates can be found at

https://journals.plos.org/plosone/s/file?id=wjVg/PLOSOne_formatting_sample_main_body.pdf and

https://journals.plos.org/plosone/s/file?id=ba62/PLOSOne_formatting_sample_title_authors_affiliations.pdf

2. Please include captions for your Supporting Information files at the end of your manuscript, and update any in-text citations to match accordingly. Please see our Supporting Information guidelines for more information: http://journals.plos.org/plosone/s/supporting-information.

3. We are unable to open your Supporting Information file “FOC_DETERMINANTS.dta”. Please kindly revise as necessary and re-upload.

4. If the reviewer comments include a recommendation to cite specific previously published works, please review and evaluate these publications to determine whether they are relevant and should be cited. There is no requirement to cite these works unless the editor has indicated otherwise.

Additional Editor Comments:

Please note that we have only been able to secure a single reviewer to assess your manuscript. We are issuing a decision on your manuscript at this point to prevent further delays in the evaluation of your manuscript. Please be aware that the editor who handles your revised manuscript might find it necessary to invite additional reviewers to assess this work once the revised manuscript is submitted. However, we will aim to proceed on the basis of this single review if possible.

The comments from the reviewer can be found below. Please review these comments and make the appropriate revisions to your manuscript alongside a point-by-point response to the concerns raised

[Note: HTML markup is below. Please do not edit.]

Reviewers' comments:

Reviewer's Responses to Questions

**Comments to the Author**

1. Is the manuscript technically sound, and do the data support the conclusions?

Reviewer #1: Yes

**********

2. Has the statistical analysis been performed appropriately and rigorously?

Reviewer #1: Yes

**********

3. Have the authors made all data underlying the findings in their manuscript fully available?

The PLOS Data policy

Reviewer #1: Yes

**********

4. Is the manuscript presented in an intelligible fashion and written in standard English?

Reviewer #1: Yes

**********

Reviewer #1: Overall Evaluation

The manuscript addresses an important maternal-health issue and adds value by examining parity-specific determinants, an angle that is genuinely under-explored in sub-Saharan Africa.

The study is generally well-structured, and the authors make good use of a validated instrument (W-DEQ). The analysis is adequate for a cross-sectional design, and the results are presented clearly.

However, there are several significant issues in methodology, clarity of writing, conceptual framing, organization, and interpretation that need attention before the manuscript reaches publication quality.

Major Issues

Abstract

• Method- what is the interest of the researcher? Is it to compare between parties? If so, does the researcher consider it in the design stage? It seems at the analysis level. If the researcher thinks that there is a difference in FoC based on parity, why not use a comparative sample?

• Conclusion- the recommendation is not aligned with the result. The determinants were not considered in the recommendation.

1. Introduction

• The introduction repeats similar ideas and includes citations that are not always well contextualized.

• The “gap” section is too long and still doesn’t clearly justify why parity-specific analysis is essential for Ghana or how it connects to previous evidence.

2. Methods

Study design, setting, and population

• Line 103-Satasi vs line 104-Santasi----check it.

Sample size estimation

• The sampling frame is unclear. Were all women attending ANC during the study days eligible? What about their time? Are they expected to stay in the clinic until 10:00 am? Is it feasible? It requires strong justification, but it is not convincing.

• Using a list “as of 10:00 a.m.” may introduce selection bias.

• Sample size calculation- the researcher uses proportion, but it is about the mean of Foc. What is the reason for choosing proportion rather than mean?

• It is not clear how many samples were calculated for nullipara and multipara.

Data collection tools and procedures

• The researchers mention that the tool was pretested on 20 samples, but didn’t indicate the justification for selecting 20 samples, as most suggested using at least 30 individuals for pretest. There is no information on which aspect of the tool was amended after the preset.

Measurement

• IPV, family support, and family abuse are measured with single-item questions. This should be acknowledged and justified earlier.

Statistical analysis

• The outcome variable is categorized into three, but the researcher used multivariable linear regression. Why not multinomial logistic regression or ordinal logistic regression?

• Authors say they included “significant and clinically relevant variables,” but the criteria are not defined.

• It's unclear whether multicollinearity was checked.

• Important variables like gestational age are missing from the model.

W-DEQ scoring

• Severity levels are stated, but the manuscript uses both continuous and categorical outputs. The rationale for mixed reporting should be clarified.

3. Results

• Response rate is not indicated in either the abstract or in the document.

• In several places, interpretation borders on causal language despite the cross-sectional design.

• What does the P-value in Table 1 and Table 2 indicate, and what is its importance?

4. Discussion

• The discussion is long, sometimes repetitive, and occasionally drifts into general maternal-health topics not directly tied to the findings.

• Some evidence is misinterpreted (e.g., the claim that parous women often report higher FoC contradicts the study’s results and global literature).

5. Writing & Language

There are recurring language and style issues:

• Long, multi-clause sentences reduce clarity.

• Occasional typos (e.g., “Wima” instead of “Wijma,” “Satasi” vs “Santasi” variation).

• Inconsistent tense and inconsistent formatting.

• Occasional grammar issues.

**********

what does this mean?). If published, this will include your full peer review and any attached files.

If you choose “no”, your identity will remain anonymous but your review may still be made public.

**Do you want your identity to be public for this peer review?**  For information about this choice, including consent withdrawal, please see our Privacy Policy

Reviewer #1: **Yes:** Teklemariam Gultie

**********

[NOTE: If reviewer comments were submitted as an attachment file, they will be attached to this email and accessible via the submission site. Please log into your account, locate the manuscript record, and check for the action link "View Attachments". If this link does not appear, there are no attachment files.]

To ensure your figures meet our technical requirements, please review our figure guidelines: https://journals.plos.org/plosone/s/figures

You may also use PLOS’s free figure tool, NAAS, to help you prepare publication quality figures: https://journals.plos.org/plosone/s/figures#loc-tools-for-figure-preparation.

NAAS will assess whether your figures meet our technical requirements by comparing each figure against our figure specifications.

Attachment Submitted filename: Reviewer comments.docx

# Author comment: Author response to Decision Letter 1

**DOI:** 10.1371/journal.pone.0358457.r002

5 Dec 2025

Overall Evaluation

Reviewer’s comment: The manuscript addresses an important maternal-health issue and adds value by examining parity-specific determinants, an angle that is genuinely under-explored in sub-Saharan Africa. The study is generally well-structured, and the authors make good use of a validated instrument (W-DEQ). The analysis is adequate for a cross-sectional design, and the results are presented clearly.However, there are several significant issues in methodology, clarity of writing, conceptual framing, organization, and interpretation that need attention before the manuscript reaches publication quality.

Authors’ response: We are very grateful to the reviewer for investing his time to review this work. It is worth noting that the reviewer has provided insightful comments that will enhance the quality of the work. We have addressed all issues raised by the reviewer in the revised manuscript. We have provided a point-by-point response to each issue raised by the reviewer below.

Major Issues

Abstract

Reviewer’s comment: Method- what is the interest of the researcher? Is it to compare between parties? If so, does the researcher consider it in the design stage? It seems at the analysis level. If the researcher thinks that there is a difference in FoC based on parity, why not use a comparative sample?

Authors’ response: We are grateful to the reviewer for this key observation. Our main aim was to explore the prevalence of FoC and identify within parity-specific determinants among antenatal care attendees. The aim of the stratification was not to compare FoC between the two groups, but rather to identify the factors associated with FoC within each parity group. The rationale for this analysis was that the general interventions which are currently practised at antenatal care may not be generally effective across all parity groups. Hence, understanding the factors associated with FoC within each parity group can inform targeted or specific interventions at antenatal care. We have provided clarity under the Study Design, Setting And Population in the revised manuscript.

The study population consisted of all pregnant women who accessed ANC at the clinic during the study period. Pregnant women who looked critically ill and required hospitalization were excluded from the study. Though the study included all women irrespective of their parity status, the rationale was not to compare the factors associated with FoC between parity groups. This method enables the identification of factors that may vary depending on parity and provides guidance for focused ANC interventions.

Reviewer’s comment: Conclusion- the recommendation is not aligned with the result. The determinants were not considered in the recommendation.

Authors’ response: We appreciate the reviewer's careful observation. The conclusion has been revised to include the determinants, as recommended by the reviewer.

FoC is common among this cohort of pregnant women, and its determinants differ by parity status. The parity-stratified determinants underscore the need for parity-tailored psychosocial care at ANC, incorporating targeted screening and support procedures aligned with parity-associated risk pathways to enhance maternal well-being.

1. Introduction

Reviewer’s comment: The introduction repeats similar ideas and includes citations that are not always well contextualized.

Authors’ response: We appreciate the reviewer for his insightful comments on the introduction. We have revised the introduction by avoiding the repetition of similar ideas and also ensured that all citations are well contextualized, as recommended by the reviewer.

Reviewer’s comment: The “gap” section is too long and still doesn’t clearly justify why parity-specific analysis is essential for Ghana or how it connects to previous evidence.

Authors’ response: The gap section of the manuscript has been revised to clarify the need for the parity-specific analysis and how it connects to the literature. The introduction has been improved and now highlights the evidence gap and rationale for the study.

2. Methods

Reviewer’s comment: Study design, setting, and population Line 103-Satasi vs line 104-Santasi----check it.

Authors’ response: We appreciate the reviewer's attention to detail. This is a typo, and we have corrected it in the revised manuscript. The correct spelling is “Santasi”

Reviewer’s comments: Sample size estimation - The sampling frame is unclear. Were all women attending ANC during the study days eligible? What about their time? Are they expected to stay in the clinic until 10:00 am? Is it feasible? It requires strong justification, but it is not convincing. Using a list “as of 10:00 a.m.” may introduce selection bias. Sample size calculation- the researcher uses proportion, but it is about the mean of Foc. What is the reason for choosing proportion rather than mean? It is not clear how many samples were calculated for nullipara and multipara.

Authors’ response: We are grateful to the reviewer for this observation. We have provided clarity in the revised manuscript. https://drive.google.com/drive/folders/13YKud2GspuPrVqwQqj038R-Fv7393bsp?usp=drive_link

On each data collection day, a list of all pregnant women who had presented and registered for ANC by 10:00 am was retrieved from the records units. A feasibility assessment conducted prior to data collection found that over 90% of pregnant women attending the clinic had registered and were already in the care process, indicating that the cut-off time was operationally feasible. All attendees typically report to the clinic between 7:00 am and 7:30 am, with clinical services starting at 8:30 am. ANC services usually last between 3 – 4 hours, covering registration, triage, midwife/clinical review, ultrasound scanning, laboratory investigation, and drug dispensing. As a result, by 10:00 am, most attendees were still progressing through the care process. The 10:00 am list was therefore used solely for daily recruitment, after which participants were randomly selected from it. This approach ensured equitable selection of both early and late arrivers across the clinic flow.

Again, we aimed to estimate the prevalence and parity-specific determinants of FoC. Hence, the single population proportion formula was used to estimate the required sample size. Although the W-DEQ is a continuous scale, prevalence was defined using a literature-based cut-off for the overall scores: low (≤ 37), moderate (38 – 65), high (66 – 84), or severe (≥ 85) levels of FoC (22). Participants who scored high or severe categories (≥66) were classified as FoC cases (23–25).

We have provided clarity in the revised manuscript. We have also provided clarity on how many samples were calculated for nullipara and multipara in the revised manuscript. Below are the revised sections in the manuscript.

The sampling frame comprised all pregnant women who accessed ANC services at the clinic during the study period. On each data collection day, a list of all pregnant women who had presented and registered for ANC by 10:00 am was retrieved from the records units. This cut-off was selected based on prior feasibility assessment showing that over 90% of pregnant women typically report to the clinic by this time, ensuring that the majority of the population is included while handling day-to-day recruitment logistics.

Despite measuring FoC as a continuous variable, this formula provides a conservative estimate adequate for prevalence and parity-stratified analysis.

We estimated the sample size for the whole cohort, without pre-specifying for nulliparous and parous women. The stratification was done at the analysis stage, enabling parity-specific examination among the recruited sample.

Reviewer’s comment: Data collection tools and procedures -The researchers mention that the tool was pretested on 20 samples, but didn’t indicate the justification for selecting 20 samples, as most suggested using at least 30 individuals for pretest. There is no information on which aspect of the tool was amended after the preset.

Authors’ response: We pretested the tool on 20 pregnant women in another facility, with the decision guided by the limited number of pregnant women within the time frame for the pretest. While in the literature some recommend 30 individuals, pretesting on 20 participants is deemed acceptable for identifying issues related to clarity, comprehension and the flow of the tool. We have also provided in the revised manuscript where the amendment was affected in the tool.

Reviewer’s comment: Measurement-IPV, family support, and family abuse are measured with single-item questions. This should be acknowledged and justified earlier.

Authors’ response: We thank the reviewer for these insightful comments. We acknowledge the use of single-item self-report questions to measure IPV, family support, and family abuse. We adopted this approach to reduce the burden on respondents while ensuring that sensitive issues like these could be addressed quickly and clearly at the ANC setting. We also acknowledged it as a limitation in the study.

These three psychosocial variables were measured using single-item self-report questions to reduce respondents’ burden and enhance clarity, particularly given their sensitive nature in an ANC setting.

Second, some variables, such as IPV, family abuse and family support, were measured using a single-item self-report, which may reduce measurement breadth and precision that could have been examined with multiple items

Statistical analysis

Reviewer’s comment: The outcome variable is categorized into three, but the researcher used multivariable linear regression. Why not multinomial logistic regression or ordinal logistic regression?

Authors’ response: We appreciate the reviewer’s careful observation. We categorized the W-DEQ scores into three levels for descriptive purposes and used the continuous W-DEQ scores for the multivariable analysis and not the categorical variable. Multivariable linear regression analysis was therefore the most suitable as it maintains the full variability and statistical power of the continuous measure.

Particularly in a moderately small sample, categorizing the result for analysis—for example, by applying multinomial or ordinal logistic regression—can result in information loss and decreased power. This has been provided in the revised manuscript.

For descriptive purposes, the primary outcome, W-DEQ scores, measured as a continuous variable, was categorized into three levels of FoC. However, a multivariable linear regression analysis was used to identify factors associated with FoC using continuous W-DEQ scores

Reviewer’s comment: Authors say they included “significant and clinically relevant variables,” but the criteria are not defined.

Authors’ response: We appreciate the reviewer for this comment. We adjusted for both significant (p < 0.2) and clinically relevant variables, based on the existing literature and theoretical effects on FoC, in the multivariable linear regression models. We adopted this approach to ensure that both statistically significant and contextually important variables were considered in the adjusted models. This has been provided in the methods of the revised manuscript. Specifically under statistical analysis

Reviewer’s comment: It's unclear whether multicollinearity was checked.

Authors’ response: We checked for multicollinearity using the Variance Inflation Factor. We have reported it under statistical analysis in the revised manuscript.

The Variance Inflation Factor (VIF) was used to assess multicollinearity among independent variables for all the multivariable models (overall, nulliparous-specific and parous-specific). The mean VIF for the overall, nulliparous-specific and parous-specific models were 2.44 (range: 1.17 to 4.80), 2.94 (range: 1.48 to 6.86) and 3.02 (range: 1.10 to 6.97), respectively. All the mean VIF values were below the conventional threshold of 10, indicating no significant multicollinearity; however, some individual variables had higher VIFs. Specifically, in the nulliparous-specific model, tertiary education had a VIF of 6.86, and in the parous-specific model, occupation (formal: 6.97; informal: 6.08) had high VIFs. These variables were retained in the models due to their theoretical significance and because they had little to no effect on the estimates of other variables.

Reviewer’s comment: Important variables like gestational age are missing from the model.

Authors’ response: We thank the reviewer for this observation and we agree with the reviewer on this. It is possible that this variable could have impacted participants FoC. However, gestational age was not collected as a study variable. We have thus acknowledged it as a limitation of the study.

Reviewer’s comment: W-DEQ scoring - Severity levels are stated, but the manuscript uses both continuous and categorical outputs. The rationale for mixed reporting should be clarified.

Authors’ response: We appreciate the reviewer for this observation. Clarity has been provided in the revised manuscript.

Severity categories were created and reported descriptively to provide clinically meaningful context to evaluate the distribution of FoC levels, instead of being used for modelling or sub-group comparison.

3. Results

Reviewer’s comment: Response rate is not indicated in either the abstract or in the document.

Authors response: We have reported it in the revised manuscript in the results of the main document.

A total of 282 of the 290 women recruited participated in the study, yielding a response rate of 97.2%

Reviewer’s comment: In several places, interpretation borders on causal language despite the cross-sectional design.

Authors’ response: We appreciate the reviewer’s comment on this. We have revised phrases like ‘independently associated with’, which could be interpreted as a causal link, to ‘significantly associated with’, which conveys statistical associations throughout the results.

Reviewer’s comment: What does the P-value in Table 1 and Table 2 indicate, and what is its importance?

Authors’ response: We are grateful to the reviewer for this observation. The p-values in Tables 1 and 2 were used to determine whether participants’ characteristics and W-DEQ scores differed significantly between the two parity groups.

4. Discussion

Reviewer’s comment: The discussion is long, sometimes repetitive, and occasionally drifts into general maternal-health topics not directly tied to the findings.

Authors’ response: We are grateful to the reviewer for his/her careful review of the discussion. We have revised the discussion to avoid repetition and to avoid drifting into general maternal health topics. We ensured that all the discussions were tied to the study findings.

Reviewer’s comment: Some evidence is misinterpreted (e.g., the claim that parous women often report higher FoC contradicts the study’s results and global literature).

Authors’ response: Again, we are grateful to the reviewer for his/her attention to detail. We have corrected the misinterpretation that parous women often report higher FoC as it was instead the opposite. The revised manuscript now interpret nulliparous women as having higher levels of FoC than parous women.

5. Writing & Language

Reviewer’s comment: There are recurring language and style issues: Long, multi-clause sentences reduce clarity. Occasional typos (e.g., “Wima” instead of “Wijma,” “Satasi” vs “Santasi” variation). Inconsistent tense and inconsistent formatting. Occasional grammar issues.

Authors’ response: Again, we are very grateful to the reviewer for his/her attention to detail. We have corrected all language and style issues. ‘Satasi’ has now been corrected to ‘Santasi’.

Attachment Submitted filename: AUTHORS RESPONSES.docx

# Peer review: Decision Letter 1

**DOI:** 10.1371/journal.pone.0358457.r003

**Authors:** Abera Mersha

27 Jan 2026

Dear Dr. Opoku,

Thank you for submitting your manuscript to PLOS ONE. After careful consideration, we feel that it has merit but does not fully meet PLOS ONE’s publication criteria as it currently stands. Therefore, we invite you to submit a revised version of the manuscript that addresses the points raised during the review process.

==============================

To be considered for publication in this journal, the methodology section requires critical revision or justification as also noted by the reviewers and the data presentation must be re-examined.

==============================

Please submit your revised manuscript by Mar 13 2026 11:59PM. If you will need more time than this to complete your revisions, please reply to this message or contact the journal office at plosone@plos.org. When you're ready to submit your revision, log on to https://www.editorialmanager.com/pone/ and select the 'Submissions Needing Revision' folder to locate your manuscript file.

- A letter that responds to each point raised by the academic editor and reviewer(s). You should upload this letter as a separate file labeled 'Response to Reviewers'.
- A marked-up copy of your manuscript that highlights changes made to the original version. You should upload this as a separate file labeled 'Revised Manuscript with Track Changes'.
- An unmarked version of your revised paper without tracked changes. You should upload this as a separate file labeled 'Manuscript'.

If you would like to make changes to your financial disclosure, please include your updated statement in your cover letter. Guidelines for resubmitting your figure files are available below the reviewer comments at the end of this letter.

If applicable, we recommend that you deposit your laboratory protocols in protocols.io to enhance the reproducibility of your results. Protocols.io assigns your protocol its own identifier (DOI) so that it can be cited independently in the future. For instructions see: https://journals.plos.org/plosone/s/submission-guidelines#loc-laboratory-protocols. Additionally, PLOS ONE offers an option for publishing peer-reviewed Lab Protocol articles, which describe protocols hosted on protocols.io. Read more information on sharing protocols at https://plos.org/protocols?utm_medium=editorial-email&utm_source=authorletters&utm_campaign=protocols.

We look forward to receiving your revised manuscript.

Kind regards,

Abera Mersha, MSc.

Academic Editor

PLOS One

Journal Requirements:

If the reviewer comments include a recommendation to cite specific previously published works, please review and evaluate these publications to determine whether they are relevant and should be cited. There is no requirement to cite these works unless the editor has indicated otherwise.

[Note: HTML markup is below. Please do not edit.]

Reviewers' comments:

Reviewer's Responses to Questions

**Comments to the Author**

Reviewer #2: All comments have been addressed

Reviewer #3: All comments have been addressed

**********

2. Is the manuscript technically sound, and do the data support the conclusions?

Reviewer #2: Partly

Reviewer #3: Yes

**********

3. Has the statistical analysis been performed appropriately and rigorously?

Reviewer #2: No

Reviewer #3: Yes

**********

4. Have the authors made all data underlying the findings in their manuscript fully available?

The PLOS Data policy

Reviewer #2: Yes

Reviewer #3: Yes

**********

5. Is the manuscript presented in an intelligible fashion and written in standard English?

Reviewer #2: No

Reviewer #3: Yes

**********

Reviewer #2: 1. The title of study is not suitable with the substances of the manuscript

2. The novelty of study need to be explained detail

3. Keywords: please add them from a term mesh, put them, and locate them alphabetically.

4. The study context is not well described, and the gap or state of the art also is not clear as well as the method

5. The suggestion is that it is better if method of this study is in qualitative design

6. The sample size calculation is unclear. What are the criteria for inclusion and exclusion in the sample study? How the researchers recruit the respondents?

7. It is unusual to find p-values in tables 1 and 2, especially on the characteristic data

8. The research relates the findings to previous work is so simple, need more references as well as their implications for practice need to be explore more

9. Need more explain significance of results, and how they contribute to the overall state of knowledge or how they advance knowledge as well

Reviewer #3: The manuscript explores a relevant topic and has potential scientific value. However, important revisions are required, particularly concerning methodological clarity, data presentation, and language quality. Addressing the comments below will help strengthen the manuscript and improve its suitability for publication

**********

what does this mean?). If published, this will include your full peer review and any attached files.

If you choose “no”, your identity will remain anonymous but your review may still be made public.

**Do you want your identity to be public for this peer review?**  For information about this choice, including consent withdrawal, please see our Privacy Policy

Reviewer #2: No

Reviewer #3: No

**********

[NOTE: If reviewer comments were submitted as an attachment file, they will be attached to this email and accessible via the submission site. Please log into your account, locate the manuscript record, and check for the action link "View Attachments". If this link does not appear, there are no attachment files.]

To ensure your figures meet our technical requirements, please review our figure guidelines: https://journals.plos.org/plosone/s/figures

You may also use PLOS’s free figure tool, NAAS, to help you prepare publication quality figures: https://journals.plos.org/plosone/s/figures#loc-tools-for-figure-preparation.

NAAS will assess whether your figures meet our technical requirements by comparing each figure against our figure specifications.

Attachment Submitted filename: pols one 1212.docx

# Author comment: Author response to Decision Letter 2

**DOI:** 10.1371/journal.pone.0358457.r004

21 Feb 2026

AUTHORS' RESPONSES TO REVIEWER’S COMMENTS

Reviewer’s comments Authors’ response

Reviewer 2

The title of study is not suitable with the substances of the manuscript We are grateful to the reviewer for this thoughtful comment. The title of the study has been revised to read as;

Fear of childbirth among antenatal care attendees in a primary care setting in Kumasi, Ghana: Prevalence and parity-stratified determinants

The novelty of study need to be explained detail We are grateful to the reviewer for drawing our attention to this. The novelty of the study has been explained in detail in the revised manuscript. It now reads as;

Notably, considering parity only as an adjustment variable may mask unique obstetric and psychosocial routes by which FoC emerges in first-time mothers compared with women with previous birth experience. This is because the factors contributing to FoC may differ across these parity groups, potentially limiting a more nuanced understanding of risk profiles and hindering the design of patient-centered antenatal counselling strategies. To the best of the authors’ knowledge, no study in Ghana has identified parity-stratified risk factors among nulliparous and parous women. This methodological approach provides an opportunity for identifying within-group risk factors to inform routine antenatal care (ANC) screening in resource-limited primary care settings.

Keywords: please add them from a term mesh, put them, and locate them alphabetically. We are grateful to the reviewer for this comment and in line with that the keywords have been revised as follows;

Keywords: Antenatal care; Fear; Ghana; Parity; Pregnancy; Risk factors

The study context is not well described, and the gap or state of the art also is not clear as well as the method We are grateful to the reviewer for this observation. The introduction has been revised to clearly describe the study context, existing knowledge gap, and methodological rationale. Specifically, we indicated that no study in Ghana has identified parity-stratified risk factors among nulliparous and parous women, providing insight into parity-specific risk profiles, which can guide patient-centered antenatal intervention in low-resource settings

The suggestion is that it is better if method of this study is in qualitative design We are grateful to the reviewer for this comment. While qualitative design could have offered the opportunity to explore their subjective experiences of childbirth fear in detail, our aim was to quantify the prevalence and also identify parity-specific determinants among antenatal care attendees in a primary care setting. Given this, a cross-sectional design was therefore more appropriate. Future studies could complement our findings using a qualitative study approach. We have included it as a study recommendation in the revised manuscript.

Future research should explore these strategies using longitudinal, interventional, and qualitative designs to guide policy and practice in Ghana and similar settings.

The sample size calculation is unclear. What are the criteria for inclusion and exclusion in the sample study? How the researchers recruit the respondents? We are grateful to the reviewer for these observations. In response, we have revised the sample size calculation to improve clarity in the manuscript. Statements on the inclusion and exclusion criteria have been added under the subheading “Study design, setting, and population.” Additionally, we created a separate subheading, “Participants’ recruitment,” to clearly describe the recruitment process, rather than including it within the “Sample size estimation and sampling technique” section as in the original submission. The recruitment procedure has been elaborated to ensure transparency and reproducibility.

It is unusual to find p-values in tables 1 and 2, especially on the characteristic data We appreciate the reviewer's comment. We understand that it is unusual to find p-values in Tables 1 and 2, especially on the characteristics data, we prefer to maintain it in its original form with the p-values. This is because the p-values provide useful insights into statistically significant differences between nulliparous and parous women, which are directly relevant to our study’s objectives. For example, they highlight where socio-demographic or obstetric characteristics differ by parity, helping to contextualize subsequent analyses and interpretation of fear of childbirth (FoC) outcomes.

As illustrated in our discussion, one notable finding among nulliparous women was the association between late ANC booking and increased FoC. Late ANC booking may limit access to early interventions, including education and psychosocial support, that can reduce fear. Evidence suggests that nulliparous women who engage in timely ANC are better prepared for childbirth, which subsequently helps to reduce FoC (41). Retaining p-values in these tables allows readers to see the statistical basis for such parity-specific observations and supports the narrative in the discussion section

The research relates the findings to previous work is so simple, need more references as well as their implications for practice need to be explore more We are grateful to the reviewer for this observation. We have provided one or two additional references in the discussion and also improved the study's implications for practice. Specifically, we have included the statement below in the section on “implications for practice and research.”

Our findings suggest that, in addition to routine ANC programs that screen for FoC, they should also integrate patient-centered strategies that account for the unique risk profiles within each parity group. There is a need for healthcare managers to train professionals to identify at-risk populations, provide patient-centered education, and involve family members when necessary to promote social support. Incorporating FoC screening tools into routine ANC guidelines could help identify vulnerable women who could benefit from additional psychological or social support strategies, thereby enhancing maternal satisfaction and childbirth preparedness.

Need more explain significance of results, and how they contribute to the overall state of knowledge or how they advance knowledge as well We appreciate the reviewer for these comments. We have revised the manuscript to reflect this.

Another critical factor was the inverse association between family support and FoC, underscoring the broader literature emphasizing the protective role of social support on maternal anxiety and childbirth-related stress (50,51). Women with strong support systems tend to feel more empowered, informed, and emotionally secure, which often results in reduced levels of fear and anxiety (52). This finding underpins the importance of social networks in improving childbirth experiences in Ghana and advances knowledge of the psychosocial factors that influence FoC in a limited-resource setting. The protective role of family support increases the understanding of how social variables shape FoC. This highlights the importance of integrating family-oriented approaches into ANC services to reduce FoC and improve maternal well-being. This finding is particularly relevant in the Ghanaian context, where extended family structures and community networks play a crucial role in shaping women’s health experiences. Encouraging family involvement in ANC and advocating for supportive environments may be essential to reducing FoC.

Reviewer 3

The study addresses a relevant research question within the scope of PLOS ONE and presents data of potential interest. However, several issues related to methodological clarity, data analysis, reporting, and language require attention to ensure transparency, reproducibility, and adherence to PLOS ONE standards. We are very grateful to the reviewer for investing his/her time to review this work. It is worth noting that the reviewer has provided insightful comments that will enhance the quality of the work.

We have addressed all issues raised by the reviewer in the revised manuscript.

We have provided a point-by-point response to each issue raised by the reviewer below.

TITLE

line 1: Fear of childbirth in Ghana: Prevalence and within parity-specific determinants among antenatal care attendees in a health facility in Kumasi, Ghana (“within parity-specific determinants” is grammatically awkward).

Suggested correction:

Fear of childbirth in Ghana: Prevalence and parity-specific determinants among antenatal care attendees in a health facility in Kumasi We are grateful to the reviewer for this observation. The title has been revised to remove redundancy and improve clarity. It now reads:

Fear of childbirth among antenatal care attendees in a primary care setting in Kumasi, Ghana: Prevalence and parity-stratified determinants

ABSTRACT

Line 34: Previous studies on Fear of Childbirth (FoC) across sub-Saharan Africa have typically reported prevalence separately by parity or treated it as a covariate in multivariable analyses, with little attention given to identifying within-parity-specific determinants (“within-parity-specific” is repetitive and awkward)

Suggested correction:

with little attention given to identifying parity-specific determinants. We are grateful to the reviewer for this observation. This phrase has been corrected in the revised manuscript

line 34-35 : Yet, first-time mothers and those with previous birth experiences may require different forms of antenatal support (“Yet,” at the beginning is acceptable but slightly informal).

Suggested correction:

However, first-time mothers and those with previous birth experiences may require different forms of antenatal support Following the reviewer’s suggestion, “Yet” has been replaced with “However” to improve academic tone.

line 43 : Stratified multivariable linear regression models were applied separately to identify parity-specific determinants among the participants(Clear, but “applied separately” is redundant)

Suggested correction:

Stratified multivariable linear regression models were applied to identify parity-specific determinants. The phrase has been revised. The sentence now reads:

“Stratified multivariable linear regression models were applied to identify parity-specific determinants.”

line 51: independently associated with FOC (Inconsistent capitalization (FoC vs FOC). Suggested correction:

independently associated with FoC. (Apply this correction throughout the manuscript.) Capitalization has been standardized to “FoC” throughout the manuscript

Conclusion

differed by parity and within parity-specific factors.

Comment:

line 57 : Redundant and grammatically awkward.

Suggested correction:

differed by parity and across parity-specific factors. This has been revised to “across parity-specific factors” to improve grammatical accuracy.

INTRODUCTION

line 67 : worry and psychological distress related to pregnancy, labour and delivery(comma consistency)

Suggested correction:

worry, and psychological distress related to pregnancy, labour, and delivery. The sentence has been revised to ensure consistent punctuation: “worry, and psychological distress related to pregnancy, labour, and delivery.”

Line 68 somatizations (British spelling is acceptable, but be consistent).

Suggested correction (optional):

somatization Spelling has been standardized to “somatization” throughout the manuscript.

line 76 : An interplay of sociodemographic, psychosocial and obstetric factors( Missing comma).

Suggested correction:

sociodemographic, psychosocial, and obstetric factors The sentence has been revised to read: “sociodemographic, psychosocial, and obstetric factors.”

Line 88-89 : few of have directly assessed the parity-specific determinants(Grammatical error or missing word).

Suggested correction:

few have directly assessed parity-specific determinants

This has been corrected to “few have directly assessed parity-specific determinants in the region

METHODS

Line 113 : Pregnant women who looked critically ill(“looked” is subjective and informal). Suggested correction:

Pregnant women who were critically ill We appreciate the reviewer's attention to detail. The phrase has been revised to “were critically ill” to ensure objective and professional language.

By adjusting for a contingency rate of 10%

Comment:

line 120 : “contingency rate” is unclear.

Suggested correction:

By adjusting for a 10% non-response rate We are grateful for this comment. This has been revised to “10% non-response rate

records units

line 125: records units (Plural unnecessary).

Suggested correction:

records unit This has been corrected to “records unit.”

Line 159 : The data collected was analyzed(Subject–verb agreement.)

Suggested correction:

The data collected were analyzed We are grateful to the reviewer. The sentence has been revised to reflect the reviewer’s recommendation.

Line 147 : the decision of ANC booking is influenced by who(Sentence fragment / unclear phrasing.)

Suggested correction:

whether the decision regarding ANC booking was influenced by another person We thank the reviewer for these comments. The sentence has been revised to clarify whether the decision regarding ANC booking was influenced by another person. It now reads as;

Additionally, the clinical factors included ANC booking, whether the decision regarding ANC booking was influenced by another person....

W-DEQ description

Line 150-151: 33- item scale(Spacing error)

Suggested correction:

33-item scale This has been corrected to “33-item scale.”

STATISTICAL ANALYSIS

(Data analysis): Subject–verb agreement error (“data was”). We appreciate the reviewer’s careful observation. This has been corrected to “The data collected were analyzed.”

Line 165-166 : Parity-stratified comparison between demographic and clinical characteristics(“comparison” should be plural or rephrased)

Suggested correction:

Parity-stratified comparisons of demographic and clinical characteristics We are grateful to the reviewer for his/her attention to detail. This correction has been effected in the revised manuscript.

Line 171 : factors that each independently associated(Missing verb).

Suggested correction:

factors that were independently associated We appreciate the reviewer's comment. This has been corrected to “factors that were independently associated.” In the revised manuscript

RESULTS

Line 190-191 : parous women (23.9 ± 3.9 years) being significantly older than nulliparous women (30.2 ± 5.0 years) ; (Values are reversed (logical error).

Suggested correction:

nulliparous women (23.9 ± 3.9 years) being significantly younger than parous women (30.2 ± 5.0 years) We thank the reviewer for identifying this error. The values have been corrected, with nulliparous women now accurately reported as younger than parous women.

ANC booking (weeks) Early (1 – 12 (Missing closing parenthesis.

Suggested correction:

Early (1–12)

38.70 (Inconsistent decimal places and missing parenthesis).

Suggested correction:

38.7) We are grateful to the reviewer for this observation. The values in the table have been revised to correct missing parentheses and standardize decimal formatting.

Discussion

Line 277 : skewedness in the distribution(Nonstandard word)

Suggested correction:

skewness in the distribution This has been corrected to the standard statistical term “skewness.”

Line 292-293 : Compared to nulliparous women, parous women often report higher levels of FoC(This contradicts earlier statements)

Suggested correction:

Compared to parous women, nulliparous women often report higher levels of FoC We are grateful to the reviewer for his/her attention to detail. We have corrected the misinterpretation that parous women often report higher FoC, as it was, instead, the opposite. The revised manuscript now interprets nulliparous women as having higher levels of FoC than parous women.

Line 332 : prevalence of fear FOC(Typographical error)

.Suggested correction:

prevalence of FoC This has been corrected to “FoC.”

Line 377 : Routine screening for FOC(Capitalization inconsistency)

Suggested correction:

Routine screening for FoC Capitalization has been standardized to “FoC” throughout the manuscript

ACKNOWLEDGEMENT

Line 384 : Medical Center's management(Spelling consistency with earlier “Centre”)

Suggested correction:

Medical Centre’s management Spelling has been standardized to “Medical Centre’s management,” consistent with earlier usage.

Attachment Submitted filename: AUTHORS RESPONSES TO REVIEWERS COMMENTS.docx

# Peer review: Decision Letter 2

**DOI:** 10.1371/journal.pone.0358457.r005

**Authors:** Paridhi Jha

27 Apr 2026

Dear Dr. Opoku,

Thank you for submitting your manuscript to PLOS ONE. After careful consideration, we feel that it has merit but does not fully meet PLOS ONE’s publication criteria as it currently stands. Therefore, we invite you to submit a revised version of the manuscript that addresses the points raised during the review process.

Please submit your revised manuscript by Jun 11 2026 11:59PM. If you will need more time than this to complete your revisions, please reply to this message or contact the journal office at plosone@plos.org. When you're ready to submit your revision, log on to https://www.editorialmanager.com/pone/ and select the 'Submissions Needing Revision' folder to locate your manuscript file.

- A letter that responds to each point raised by the academic editor and reviewer(s). You should upload this letter as a separate file labeled 'Response to Reviewers'.
- A marked-up copy of your manuscript that highlights changes made to the original version. You should upload this as a separate file labeled 'Revised Manuscript with Track Changes'.
- An unmarked version of your revised paper without tracked changes. You should upload this as a separate file labeled 'Manuscript'.

If you would like to make changes to your financial disclosure, please include your updated statement in your cover letter. Guidelines for resubmitting your figure files are available below the reviewer comments at the end of this letter.

If applicable, we recommend that you deposit your laboratory protocols in protocols.io to enhance the reproducibility of your results. Protocols.io assigns your protocol its own identifier (DOI) so that it can be cited independently in the future. For instructions see: https://journals.plos.org/plosone/s/submission-guidelines#loc-laboratory-protocols. Additionally, PLOS ONE offers an option for publishing peer-reviewed Lab Protocol articles, which describe protocols hosted on protocols.io. Read more information on sharing protocols at https://plos.org/protocols?utm_medium=editorial-email&utm_source=authorletters&utm_campaign=protocols.

As the corresponding author, your ORCID iD is verified in the submission system and will appear in the published article. PLOS supports the use of ORCID, and we encourage all coauthors to register for an ORCID iD and use it as well. Please encourage your coauthors to verify their ORCID iD within the submission system before final acceptance, as unverified ORCID iDs will not appear in the published article. *Only*  the individual author can complete the verification step; PLOS staff *cannot*  verify ORCID iDs on behalf of authors.

We look forward to receiving your revised manuscript.

Kind regards,

Paridhi Jha, PhD

Academic Editor

PLOS One

**Journal Requirements:**

1. If the reviewer comments include a recommendation to cite specific previously published works, please review and evaluate these publications to determine whether they are relevant and should be cited. There is no requirement to cite these works unless the editor has indicated otherwise.

2. Please review your reference list to ensure that it is complete and correct. If you have cited papers that have been retracted, please include the rationale for doing so in the manuscript text, or remove these references and replace them with relevant current references. Any changes to the reference list should be mentioned in the rebuttal letter that accompanies your revised manuscript. If you need to cite a retracted article, indicate the article’s retracted status in the References list and also include a citation and full reference for the retraction notice.

[Note: HTML markup is below. Please do not edit.]

Reviewers' comments:

Reviewer's Responses to Questions

**Comments to the Author**

Reviewer #3: All comments have been addressed

Reviewer #4: All comments have been addressed

**********

2. Is the manuscript technically sound, and do the data support the conclusions?

Reviewer #3: Yes

Reviewer #4: Yes

**********

3. Has the statistical analysis been performed appropriately and rigorously?

Reviewer #3: Yes

Reviewer #4: Yes

**********

4. Have the authors made all data underlying the findings in their manuscript fully available?

The PLOS Data policy

Reviewer #3: Yes

Reviewer #4: Yes

**********

5. Is the manuscript presented in an intelligible fashion and written in standard English?

Reviewer #3: Yes

Reviewer #4: Yes

**********

Reviewer #3: The manuscript is well written and addresses an important topic. The objectives are clearly stated, and the study contributes valuable information to the field. The methodology is generally appropriate, and the results are presented in a logical and organized manner.

Reviewer #4: As I am viewing an already revised manuscript, I feel the author has addressed all the reviewers' concerns/ comments given in the initial reviews except for some minor typographical errors which might have introduced or missed during the revision.

**********

what does this mean?). If published, this will include your full peer review and any attached files.

If you choose “no”, your identity will remain anonymous but your review may still be made public.

**Do you want your identity to be public for this peer review?**  For information about this choice, including consent withdrawal, please see our Privacy Policy

Reviewer #3: **Yes:** Hadis Derbew Hagos

Reviewer #4: **Yes:** Dr. Konthoujam Ushakiran Devi

**********

[NOTE: If reviewer comments were submitted as an attachment file, they will be attached to this email and accessible via the submission site. Please log into your account, locate the manuscript record, and check for the action link "View Attachments". If this link does not appear, there are no attachment files.]

To ensure your figures meet our technical requirements, please review our figure guidelines: https://journals.plos.org/plosone/s/figures

You may also use PLOS’s free figure tool, NAAS, to help you prepare publication quality figures: https://journals.plos.org/plosone/s/figures#loc-tools-for-figure-preparation.

NAAS will assess whether your figures meet our technical requirements by comparing each figure against our figure specifications.

Attachment Submitted filename: PLOSONE_review on FoC.docx

# Author comment: Author response to Decision Letter 3

**DOI:** 10.1371/journal.pone.0358457.r006

28 Apr 2026

Comment: The authors have thoroughly addressed the previous reviewers’ comments and successfully resolved the technical and methodological issues in the revised manuscript. However some minor typographical errors might have introduced or missed during the revision process and hence suggesting some minor corrections.

Response: We are grateful to you, the other reviewer and the editor for ensuring that the quality and rigour of our manuscript is improved. We strongly believe that your comments have been extremely helpful in improving the quality of the manuscript, and we appreciate them.

Comment: Page 2, Line 34: using hyphen (-) between identifying and parity-specific determinants “identifying-parity-specific determinants”

Suggested correction: identifying parity-specific determinants

Response: We are grateful to the reviewer for drawing our attention to this. We statement in reference has been revised and it now reads as;

Previous studies on Fear of Childbirth (FoC) across sub-Saharan Africa have typically reported prevalence separately by parity or treated it as a covariate in multivariable analyses, with little attention given to identifying parity-specific determinants

Comment: Line 34: Misspelling of “Howerver”

Response: This has been corrected in the revised manuscript, and it now reads as;

However, first-time mothers and those with previous birth experiences may require different forms of antenatal support

Comment: Page 3, Line 67: worry, and psychological distress related to pregnancy, labor, and delivery.

Suggested correction: Please replace the word “delivery” with “childbirth” to more accurately reflect the clinical context discussed in this section.

Response: We are grateful to the reviewer for this comment, and in line with that, the statement in reference has been revised to read as;

It involves persistent feelings of anxiety, worry, and psychological distress related to pregnancy, labour, and childbirth

Comment: Page 6, Line 170: spacing between the word “analyzedand”

Response: This has been done as recommended. It now reads as;

The data collected were analysed and used to amend the questionnaire before it was used in the final study.

Attachment Submitted filename: AUTHORS RESPONSES TO REVIEWERS COMMENTS.pdf

# Peer review: Decision Letter 3

**DOI:** 10.1371/journal.pone.0358457.r007

**Authors:** Jen Edwards

15 May 2026

Dear Dr. Opoku,

Thank you for submitting your manuscript to PLOS ONE. After careful consideration, we feel that it has merit but does not fully meet PLOS ONE’s publication criteria as it currently stands. Therefore, we invite you to submit a revised version of the manuscript that addresses the points raised during the review process.

The manuscript has been evaluated by two reviewers and their comments are available below. The reviewers describe a number of formatting and typographical errors which must be corrected to ensure clarity throughout the manuscript. Could you please revise the manuscript to carefully address the concerns raised?

Please submit your revised manuscript by Jun 28 2026 11:59PM. If you will need more time than this to complete your revisions, please reply to this message or contact the journal office at plosone@plos.org. When you're ready to submit your revision, log on to https://www.editorialmanager.com/pone/ and select the 'Submissions Needing Revision' folder to locate your manuscript file.

- A letter that responds to each point raised by the academic editor and reviewer(s). You should upload this letter as a separate file labeled 'Response to Reviewers'.
- A marked-up copy of your manuscript that highlights changes made to the original version. You should upload this as a separate file labeled 'Revised Manuscript with Track Changes'.
- An unmarked version of your revised paper without tracked changes. You should upload this as a separate file labeled 'Manuscript'.

If you would like to make changes to your financial disclosure, please include your updated statement in your cover letter. Guidelines for resubmitting your figure files are available below the reviewer comments at the end of this letter.

If applicable, we recommend that you deposit your laboratory protocols in protocols.io to enhance the reproducibility of your results. Protocols.io assigns your protocol its own identifier (DOI) so that it can be cited independently in the future. For instructions see: https://journals.plos.org/plosone/s/submission-guidelines#loc-laboratory-protocols. Additionally, PLOS ONE offers an option for publishing peer-reviewed Lab Protocol articles, which describe protocols hosted on protocols.io. Read more information on sharing protocols at https://plos.org/protocols?utm_medium=editorial-email&utm_source=authorletters&utm_campaign=protocols.

As the corresponding author, your ORCID iD is verified in the submission system and will appear in the published article. PLOS supports the use of ORCID, and we encourage all coauthors to register for an ORCID iD and use it as well. Please encourage your coauthors to verify their ORCID iD within the submission system before final acceptance, as unverified ORCID iDs will not appear in the published article. *Only*  the individual author can complete the verification step; PLOS staff *cannot*  verify ORCID iDs on behalf of authors.

We look forward to receiving your revised manuscript.

Kind regards,

Jen Edwards

Staff Editor

PLOS One

Journal Requirements:

If the reviewer comments include a recommendation to cite specific previously published works, please review and evaluate these publications to determine whether they are relevant and should be cited. There is no requirement to cite these works unless the editor has indicated otherwise.

Please review your reference list to ensure that it is complete and correct. If you have cited papers that have been retracted, please include the rationale for doing so in the manuscript text, or remove these references and replace them with relevant current references. Any changes to the reference list should be mentioned in the rebuttal letter that accompanies your revised manuscript. If you need to cite a retracted article, indicate the article’s retracted status in the References list and also include a citation and full reference for the retraction notice.

[Note: HTML markup is below. Please do not edit.]

Reviewers' comments:

Reviewer's Responses to Questions

**Comments to the Author**

Reviewer #3: All comments have been addressed

Reviewer #4: All comments have been addressed

**********

2. Is the manuscript technically sound, and do the data support the conclusions?

Reviewer #3: Yes

Reviewer #4: Yes

**********

3. Has the statistical analysis been performed appropriately and rigorously?

Reviewer #3: Yes

Reviewer #4: Yes

**********

4. Have the authors made all data underlying the findings in their manuscript fully available?

The PLOS Data policy

Reviewer #3: Yes

Reviewer #4: Yes

**********

5. Is the manuscript presented in an intelligible fashion and written in standard English?

Reviewer #3: Yes

Reviewer #4: Yes

**********

Reviewer #3: Reviewer Comment to Authors

The manuscript addresses an important and relevant maternal health issue by examining the prevalence and parity-specific determinants of fear of childbirth among pregnant women attending antenatal care in Ghana. The study is well structured, methodologically sound, and contributes valuable evidence from a low-resource setting where limited data currently exist. The parity-stratified analytical approach is a notable strength of the study, as it provides a more nuanced understanding of the psychosocial and obstetric factors associated with fear of childbirth among nulliparous and parous women. The manuscript is generally clear, and the findings have potential implications for targeted antenatal interventions and maternal mental health services.

However, several issues should be addressed before the manuscript is considered for publication. These include language and grammatical inconsistencies, formatting problems within the tables and references, clarification of some methodological and statistical reporting issues, and improvement in consistency of terminology and presentation style throughout the manuscript. Additionally, a few interpretations in the discussion section require refinement to avoid overstatement of causal inference due to the cross-sectional design.

Overall, this is a valuable and potentially publishable study. Addressing the comments and recommendations provided below would substantially improve the scientific clarity, methodological transparency, and overall quality of the manuscript.

Main Corrections Made in abstract

• Corrected “firsttime” → “first-time”

• Improved flow and academic wording

• Standardized statistical reporting:

• “adj. β” → “adjusted β”

• “95%CI” → “95% CI”

• Corrected punctuation and spacing

• Standardized W-DEQ formatting

• Reduced repetition in the conclusion

• Improved readability and journal-style tone

Major Issues to Correct

1. Spacing and Formatting Errors

There are several missing spaces throughout the manuscript.

Examples

• “parityspecific” → parity-specific

• “singleitem” → single-item

• “33item” → 33-item

• “nulliparousspecific” → nulliparous-specific

• “parousspecific” → parous-specific

These should be corrected throughout the document.

Section-by-Section Review

INTRODUCTION

Paragraph 1

Original

It can manifest through emotional symptoms such as mood swings, somatisation, and cognitive effects like impaired decision-making

Suggestion

It may manifest through emotional symptoms such as mood swings and somatisation, as well as cognitive effects including impaired decision-making.

Reason:

• More formal academic wording

• Better sentence rhythm

Paragraph 2

Original

However, direct prevalence and determinants data for Ghana are limited

Suggestion

However, data on the prevalence and determinants of FoC in Ghana remain limited.

More natural scientific expression.

Original

cultural and mental health factors – such as anxiety and depression – and obstetric history

Suggestion

cultural, mental health (e.g., anxiety and depression), and obstetric factors

Cleaner academic structure.

Paragraph 3

Original

may mask unique obstetric and psychosocial routes

Suggestion

may mask unique obstetric and psychosocial pathways

“Pathways” is more appropriate in epidemiological writing.

Original

women with previous birth experience

Suggestion

women with previous birth experiences

Grammatically smoother.

Final Paragraph

Original

Identifying parity-specific evidence provides guidance

Suggestion

Identifying parity-specific determinants may provide guidance

More precise scientifically.

METHODS

Study Design Section

Original

between 20th January 2025 and 12th March 2025

Suggested journal style

between January 20, 2025, and March 12, 2025

Most international journals prefer this format.

Original

operates Monday to Friday

Suggestion

operates from Monday to Friday

Minor grammar improvement.

Sample Size Estimation

Important Statistical Issue

Original

( = (^2 ()(1 − ))/^2)

Problem

The formula notation is not standard.

Recommended Version

Use:

n=Z2P(1−P)E2n=E2Z2P(1−P)

Or in plain text:

The sample size was estimated using the single population proportion formula:

n = Z²P(1−P)/E²

Also:

• “s” should be “n”

• Better mathematical presentation needed for journal publication

Recruitment Procedure

Excellent overall.

Small improvement

averaging 12 participants per day

Could become:

with an average recruitment of 12 participants per day

Data Collection Tools and Procedures

Original

The investigators developed the questionnaire after reviewing relevant literature

Suggestion

The questionnaire was developed after a review of relevant literature

More concise and formal.

Important Consistency Issue

Original

experiencing intimate partner violence (IPV)

Later you use:

intimate partner violence

Better to define abbreviation once and consistently use:

• intimate partner violence (IPV)

• then IPV afterward

Original

measured using singleitem self-report questions

Correct

measured using single-item self-report questions

W-DEQ Section

Very strong.

Minor Improvement

Original

with minimum and maximum scores of 0 and 165

Suggested

with total scores ranging from 0 to 165

Cleaner wording.

Original

Participants who scored high or severe categories

Suggested

Participants classified within the high or severe categories

More grammatical.

Statistical Analysis Section

This section is strong statistically.

Important Clarity Improvement

Original

We adjusted for both significant (p < 0.2) and clinically relevant variables

Suggested

Variables with p-values < 0.20 in bivariable analyses, together with clinically relevant variables identified from existing literature, were included in the multivariable models.

This is more standard in epidemiological reporting.

Original

theoretical effects on FoC

Suggested

theoretical relevance to FoC

More appropriate wording.

VIF Section

This is detailed and transparent, which reviewers usually appreciate.

However, this sentence is too long.

Original

All mean VIFs were below the conventional threshold...

Break into two sentences.

Suggested

All mean VIFs were below the conventional threshold of 10, indicating no substantial multicollinearity. However, some individual variables demonstrated relatively high VIF values.

Then continue with the examples.

Ethical Approval

Strong section.

Minor Style Improvement

Original

Written approval was sought from the study site's management.

Suggested

Written permission was obtained from the management of the study site.

More formal.

Important Journal-Level Recommendations

1. Add Subheading Consistency

Ensure all subheadings follow one style consistently:

• Sentence case OR title case

Currently mixed.

Example:

• “Study design, setting, and population”

• “Sample size estimation”

These are sentence case (good). Keep all the same.

2. Clarify ANC Booking Definition

You mention:

late ANC booking

But no operational definition is given.

Reviewers may ask:

• What gestational age defines “late”?

You should define this in Methods.

Example:

Late ANC booking was defined as initiation of ANC after the first trimester (>12 weeks gestation).

(Only if that matches your study definition.)

3. Consider Mentioning Model Assumptions

Since you used linear regression, reviewers may ask about:

• normality

• homoscedasticity

• residual checks

Even one sentence helps:

Linear regression assumptions were assessed using residual diagnostics and were adequately satisfied.

IMPORTANT ISSUES TO CORRECT

1. Remove Line Numbers from the Main Text

Examples:

• “229”

• “230”

• “271”

• “275”

These should not appear inside the manuscript text unless journal submission format specifically requires tracked line numbering.

2. Table Formatting Problems

Several table rows lost formatting.

Example:

Current

Unemployed 1.00 1.00 Formal -1.24...

Correct

This should appear as separate rows:

Occupation Nulliparous Adj. β (95% CI) p-value Parous Adj. β (95% CI) p-value

Unemployed 1.00 1.00

Formal -1.24 (-10.59, 8.10) 0.792 -4.41 (-13.14, 4.33) 0.321

Informal -3.27 (-13.79, 7.25) 0.538 -7.48 (-15.24, 0.29) 0.059

This formatting issue must be corrected before submission.

RESULTS SECTION REVIEW

Opening Paragraph

Original

A total of 282 of the 290 women recruited participated in the study

Suggested

Of the 290 women recruited, 282 participated in the study

Cleaner journal style.

Original

Of this, 35.1% were nulliparous

Suggested

Of these participants, 35.1% were nulliparous

Better grammar.

Original

A little over half (50.8%)

Suggested

Approximately half (50.8%)

More academic wording.

Prevalence Section

Original

Overall, the prevalence of FoC among the study participants was 22.0% (≥66)

Suggested

Overall, 22.0% of the participants had high-to-severe FoC (W-DEQ score ≥66).

Much clearer.

Original

significantly higher median W-DEQ scores

Good.

However, you should specify the statistical test.

Example:

...reported significantly higher median W-DEQ scores than parous women (p = 0.004).

Table 2 Problems

The percentages are incomplete.

Example:

(28.3)

(45.4)

The frequencies are missing.

Should be:

• 28 (28.3%)

• etc.

Reviewers will definitely notice this.

Regression Results Section

IMPORTANT STATISTICAL REPORTING ISSUE

Current

β: -1.25, 95%CI: -1.74, 0.77

This is incorrect because:

• CI needs “to” or parentheses format

Correct format:

β = -1.25, 95% CI: -1.74 to -0.77

OR

β = -1.25 (95% CI: -1.74, -0.77)

Currently the upper limit appears positive because of punctuation confusion.

This issue appears multiple times.

Another Important Error

Current

planning current pregnancy (β: 25.71...)

But later:

not planning the current pregnancy (adj. β: 8.84...)

These contradict each other.

Likely correction:

• unadjusted model should refer to:

not planning the current pregnancy

This inconsistency must be corrected.

Family Support Result

Current

having moderate (adj. β: -13.10, 95%CI: 18.70, -7.50):

Incorrect CI order.

Should be:

adjusted β = -13.10, 95% CI: -18.70 to -7.50

Another Grammar Issue

Current

family’s support

Use:family support

throughout manuscript.

Avoid apostrophe.

Table 3 Review

Very strong overall.

Small consistency fixes

Use one reporting style consistently:

Either:

• Adj. β

OR

• adjusted β

Choose one style.

Current

1:00 = reference category

Correct:

1.00 = reference category

Table 4 Review

Strong scientifically.

Grammar issue

Current

family support was significantly associated

Plural subject problem.

Correct:

family support were significantly associated

OR better:

moderate and strong family support were significantly associated with FoC.

DISCUSSION SECTION REVIEW

This section is strong and thoughtful.

Opening Paragraph

Excellent summary.

Small improvement:

Current

underpin a critical need

Suggested:

highlight a critical need

More natural wording.

Paragraph Comparing Prevalence

Current

median WDEQ score

Should be:

median W-DEQ score

Consistency issue.

Important Statistical Issue

Current

median W-DEQ score in our study was 41, comparable to Malawi (mean: 47)

Median and mean are not directly comparable.

Suggested:

...although direct comparison should be interpreted cautiously because the Malawi study reported mean rather than median scores.

Very important for reviewers/statisticians.

Nulliparous Explanation Paragraph

Very good.

IPV Paragraph

Excellent interpretation.

Family Support Paragraph

Scientifically strong but repetitive.

You mention:

• importance of family support

• social networks

• supportive environments

several times.

You can shorten by ~25% without losing meaning.

IMPLICATIONS SECTION

Strong practical implications.

Grammar Issue

Current

they should also integrate patient-centred strategies

Problem:

Who is “they”?

Suggested:

routine ANC programs should also integrate patient-centred strategies

LIMITATIONS SECTION

Very good and transparent.

CONCLUSION REVIEW

Your conclusion is scientifically sound and appropriately balanced.

Main Strength

You avoided overstating causality, which is excellent for a cross-sectional study.

IMPORTANT ISSUES TO CORRECT

1. Typographical Error

Current

parityspecific risk profiles

Correct

parity-specific risk profiles

2. Repetitive Wording

Current

These findings underscore the need for parity-specific strategies in addressing FoC during ANC.

Suggested

These findings underscore the need for parity-tailored strategies to address FoC during ANC.

More concise and smoother.

3. Slight Overstatement

Current

could collectively reduce childbirth-related fear and promote better maternal health outcomes

Since your study is observational, avoid implying intervention effectiveness too strongly.

Better

may help reduce childbirth-related fear and improve maternal well-being.

ACKNOWLEDGEMENT REVIEW

Major Typographical Error

Current

employeesmanagement and staff

This appears to be a copy-paste duplication error.

Correct Version

We would like to thank the management and staff of HopeXchange Medical Centre, the study participants, and the research assistants for their valuable contributions to this study.

REFERENCES REVIEW

Your references are strong overall, current, and relevant.

However, several important formatting and consistency issues need correction.

MAJOR REFERENCE ISSUES

1. Duplicate Reference

Reference 11 and 28 are duplicates

Both are:

Khwepeya M, Lee GT...

You must remove one duplicate reference and renumber accordingly.

2. Typographical Error in Reference 22

Current

Wjma K.

Correct

Wijma K.

Very important because this is the main W-DEQ reference.

3. Broken Reference

Reference 48

Current

261&partnerID=40&md5=32f6a261400d8df1c8fb50cd1fad5f96

This is clearly accidental database export text and must be deleted.

4. Broken Supporting Information Section

Current

Datasets used f

Incomplete sentence.

Correct or remove.

Example:

Supporting information

The datasets used and/or analysed during the current study are available from the corresponding author upon reasonable request.

(Only if true.)

5. Inconsistent Journal Formatting

Examples:

• “BMC Womens Women's Health”

• “Front Public Heal.”

• “Women and Birth.”

• “J Obstet Gynaecol (Lahore).”

Journal abbreviations should follow one indexing style consistently (usually PubMed/NLM).

6. Inconsistent Use of Page Ranges

Examples:

• “763– 7”

• “121–6”

Need consistent formatting.

Example:

• 763–767

• 121–126

depending on journal style.

7. Capitalization Inconsistency

Example:

The Influence of Fear During Pregnancy

Some references use sentence case, others title case.

Most journals require sentence case.

Example:

Correct:

The influence of fear during pregnancy, labour and delivery on birth outcomes among post-delivery women.

3. W-DEQ Formatting

Always use:

• W-DEQ

Not:

• WDEQ

Reviewer #4: I would like to thank the authors for their response and the effort they have put in to revise the manuscript and also they have addressed all the concerns raised in the review.

**********

what does this mean?). If published, this will include your full peer review and any attached files.

If you choose “no”, your identity will remain anonymous but your review may still be made public.

**Do you want your identity to be public for this peer review?**  For information about this choice, including consent withdrawal, please see our Privacy Policy

Reviewer #3: **Yes:** Hadis Derbew Hagos

Reviewer #4: **Yes:** Dr. Konthoujam Ushakiran Devi

**********

[NOTE: If reviewer comments were submitted as an attachment file, they will be attached to this email and accessible via the submission site. Please log into your account, locate the manuscript record, and check for the action link "View Attachments". If this link does not appear, there are no attachment files.]

To ensure your figures meet our technical requirements, please review our figure guidelines: https://journals.plos.org/plosone/s/figures

You may also use PLOS’s free figure tool, NAAS, to help you prepare publication quality figures: https://journals.plos.org/plosone/s/figures#loc-tools-for-figure-preparation.

NAAS will assess whether your figures meet our technical requirements by comparing each figure against our figure specifications.

Attachment Submitted filename: PLOSONE_review.docx

Attachment Submitted filename: PONE-D-25-51554_R3 1111.docx

# Author comment: Author response to Decision Letter 4

**DOI:** 10.1371/journal.pone.0358457.r008

19 May 2026

Reviewer 3

Comment: Overall, this is a valuable and potentially publishable study. Addressing the comments and recommendations provided below would substantially improve the scientific clarity, methodological transparency, and overall quality of the manuscript.

Response: We are grateful to the reviewers for their time, and we believe that their comments were extremely valuable to improving the rigour of the manuscript. We have addressed all comments raised by the reviewers and provided point-by-point responses

Comment: Main Corrections Made in Abstract. • Corrected “firsttime” → “first-time”

Improved flow and academic wording, Standardized statistical reporting: “adj. β” → “adjusted β” “95%CI” → “95% CI” Corrected punctuation and spacing Standardized W-DEQ formatting Reduced repetition in the conclusion, Improved readability and journal-style tone

Response: We are grateful to the reviewer for these important observations. We have addressed all these comments in the revised manuscript to improve the readability and journal-style tone.

Comment: Major Issues to Correct

Spacing and Formatting Errors. There are several missing spaces throughout the manuscript.

Examples

•“parityspecific” → parity-specific

•“singleitem” → single-item

•“33item” → 33-item

•“nulliparousspecific” → nulliparous-specific

•“parousspecific” → parous-specific

These should be corrected throughout the document.

Response: We are grateful to the reviewer for these insightful comments. We respectfully note that we corrected all the cited spacing in the previous revision and that it has already been consistently applied throughout the manuscript. We have rechecked the manuscript again to ensure that these corrections remain correctly implemented.

Comment: Introduction. Paragraph 1. Original- It can manifest through emotional symptoms such as mood swings, somatisation, and cognitive effects like impaired decision-making

Suggestion: It may manifest through emotional symptoms such as mood swings and somatisation, as well as cognitive effects including impaired decision-making.

Reason: More formal academic wording, Better sentence rhythm

Response: We are grateful to the reviewer for these important observations. These sentences have been revised to reflect his/her suggestions.

Comment: paragraph 2. Original_ However, direct prevalence and determinants data for Ghana are limited. Suggestion - However, data on the prevalence and determinants of FoC in Ghana remain limited. More natural scientific expression.

Original-cultural and mental health factors – such as anxiety and depression – and obstetric history. Suggestion- cultural, mental health (e.g., anxiety and depression), and obstetric factors

Cleaner academic structure.

Response: We are grateful to the reviewer for these important observations. These sentences have been revised to reflect his/her suggestions.

Comment: Paragraph 3. Original- may mask unique obstetric and psychosocial routes Suggestion- may mask unique obstetric and psychosocial pathways

“Pathways” is more appropriate in epidemiological writing.

Original- women with previous birth experience Suggestion- women with previous birth experiences. Grammatically smoother.

Response: We are grateful to the reviewer for these important observations. These phrases have been corrected in the revised manuscript

Comment: Final Paragraph -Original - Identifying parity-specific evidence provides guidance Suggestion- Identifying parity-specific determinants may provide guidance More precise, scientifically.

Response: We are grateful to the reviewer for this important observation. This phrase have been corrected in the revised manuscript

Comment: Study Design Section. Original - between 20th January 2025 and 12th March 2025 Suggested - journal style between January 20, 2025, and March 12, 2025

Most international journals prefer this format. Original operates Monday to Friday

Suggestion -operates from Monday to Friday. Minor grammar improvement.

Response: We appreciate the reviewer's observations, and in line with them, the sentences in question have been revised as suggested. Thank you

Comment: Sample Size Estimation Important Statistical Issue. Original (= (^2 ()(1 − ))/^2) Problem -The formula notation is not standard.

Recommended Version. Use: n=Z2P(1−P)E2n=E2Z2P(1−P) Or in plain text:

The sample size was estimated using the single population proportion formula: n = Z²P(1−P)/E²

Also: “s” should be “n” Better mathematical presentation needed for journal publication

Recruitment Procedure. Excellent overall.

Response: We appreciate the reviewer's observations, and in line with them, the formula for the sample size estimation has been formatted as suggested. Thank you

Comment: Small improvement averaging 12 participants per day

Could become: with an average recruitment of 12 participants per day

Response: We appreciate the reviewer's observation, and in line with it, the sentence in question has been revised as suggested. Thank you

Comment: Data Collection Tools and Procedures. Original -The investigators developed the questionnaire after reviewing relevant literature Suggestion- The questionnaire was developed after a review of relevant literature. More concise and formal.

Response: We appreciate the reviewer's observation, and in line with it, the sentence in question has been revised as suggested. Thank you

Comment: Important Consistency Issue Original experiencing intimate partner violence (IPV)

Later you use: intimate partner violence. Better to define abbreviation once and consistently use: intimate partner violence (IPV) then IPV afterward

Response: We appreciate the reviewer's observation. We have used ‘IPV’ consistently throughout the manuscript after first defining the abbreviation in the revised manuscript.

Comment: Original measured using singleitem self-report questions. Correct - measured using single-item self-report questions

Response: We are grateful to the reviewer for this observation. We respectfully note that we corrected all the cited spacing in the previous revision and that it has already been consistently applied throughout the manuscript. We have rechecked the manuscript again to ensure that this correction remains correctly implemented.

Comment: W-DEQ Section. Very strong. Minor Improvement. Original

with minimum and maximum scores of 0 and 165 Suggested - with total scores ranging from 0 to 165. Cleaner wording.

Response: We appreciate the reviewer's observations, and in line with them, the sentences in question have been revised as suggested. Thank you

Comment: Original. Participants who scored high or severe categories

Suggested- Participants classified within the high or severe categories

Response: We appreciate the reviewer's observations, and in line with them, the sentences in question have been revised as suggested. Thank you

Comment: Statistical Analysis Section This section is strong statistically.

Important Clarity Improvement. Original. We adjusted for both significant (p < 0.2) and clinically relevant variables. Suggested - Variables with p-values < 0.20 in bivariable analyses, together with clinically relevant variables identified from existing literature, were included in the multivariable models. This is more standard in epidemiological reporting.

Response: We appreciate the reviewer's important observation. We have revised the statement in question based on the reviewer’s suggestion.

Comment: Original theoretical effects on FoC

Suggested - theoretical relevance to FoC. More appropriate wording.

Response: We appreciate the reviewer for this comment. The sentence in question has been revised as;

Variables with p-values < 0.20 in bivariable analyses, together with clinically and theoretically relevant variables identified from existing literature, were included in the multivariable models

Comment: VIF Section This is detailed and transparent, which reviewers usually appreciate. However, this sentence is too long. Original All mean VIFs were below the conventional threshold... Break into two sentences. Suggested -All mean VIFs were below the conventional threshold of 10, indicating no substantial multicollinearity. Then continue with the examples.

Response: We appreciate the reviewer's observations, and in line with them, the sentences in question have been revised as suggested. Thank you

Comment: Ethical Approval Strong section. Minor Style Improvement

Original Written approval was sought from the study site's management.

Suggested- Written permission was obtained from the management of the study site. More formal.

Response: We appreciate the reviewer's important observation. We have revised the statement in question based on the reviewer’s suggestion.

Comment: Important Journal-Level Recommendations. Add Subheading Consistency

Ensure all subheadings follow one style consistently: Sentence case OR title case Currently mixed. Example: “Study design, setting, and population” “Sample size estimation”

These are sentence case (good). Keep all the same

Response: We are grateful to the reviewer for these observations, and in line with that, we have ensured that all subheadings follow one style consistently

Comment: . Clarify ANC Booking Definition You mention: late ANC booking

But no operational definition is given. Reviewers may ask: What gestational age defines “late”?

You should define this in Methods. Example: Late ANC booking was defined as initiation of ANC after the first trimester (>12 weeks gestation). (Only if that matches your study definition.)

Response: We are grateful to the reviewer for this comment. We have provided an operational definition for late ANC booking in the revised manuscript. It reads as;

ANC booking was categorised as late and early bookings, with late booking defined as initiation of ANC after the first trimester (>12 weeks of gestation) (22).

Comment: Consider Mentioning Model Assumptions. Since you used linear regression, reviewers may ask about: normality homoscedasticity residual checks. Even one sentence helps: Linear regression assumptions were assessed using residual diagnostics and were adequately satisfied.

Response: We are grateful to the reviewer for these insightful comments. We checked these assumptions and have included a statement in the statistical analysis in the results. It reads as;

Linear regression assumptions were assessed using residual diagnostics and were found to be adequately satisfied

Comment: IMPORTANT ISSUES TO CORRECT. Remove Line Numbers from the Main Text. Examples: “229”, “230”, “271”, “275”. These should not appear inside the manuscript text unless journal submission format specifically requires tracked line numbering.

Response: We appreciate the reviewer’s comment. However, the inclusion of continuous line numbers in the manuscript follows the journal’s submission requirements. According to the PLOS ONE author guidelines, manuscripts should include both page numbers and continuous line numbering to facilitate the peer-review process. Therefore, the line numbers have been retained in the revised manuscript. Thank you.

Comment: Table Formatting Problems. Several table rows lost formatting. Example: Current Unemployed 1.00 1.00 Formal -1.24... Correct. This should appear as separate rows: Occupation Nulliparous Adj. β (95% CI) p-value Parous Adj. β (95% CI) p-value. Unemployed 1.00 1.00

Formal -1.24 (-10.59, 8.10) 0.792 -4.41 (-13.14, 4.33) 0.321

Informal -3.27 (-13.79, 7.25) 0.538 -7.48 (-15.24, 0.29) 0.059

This formatting issue must be corrected before submission.

Response: We are grateful to the reviewer for these comments. We note that the affected variables were already presented on separate rows in the submitted tables. We have reformatted the tables to ensure alignment in the revised manuscript to improve clarity.

Comment: Opening Paragraph Original A total of 282 of the 290 women recruited participated in the study. Suggested- Of the 290 women recruited, 282 participated in the study Cleaner journal style.

Response: We appreciate the reviewer's important observation. We have revised the statement in question based on the reviewer’s suggestion.

Comment: Original - Of this, 35.1% were nulliparous Suggested Of these participants, 35.1% were nulliparous Better grammar.

Response: We appreciate the reviewer's important observation. We have revised the statement in question based on the reviewer’s suggestion.

Comment: Original A little over half (50.8%). Suggested Approximately half (50.8%). More academic wording. Prevalence Section

Response: We appreciate the reviewer's important observation. We have revised the statement in question based on the reviewer’s suggestion.

Comment: Original Overall, the prevalence of FoC among the study participants was 22.0% (≥66). Suggested - Overall, 22.0% of the participants had high-to-severe FoC (W-DEQ score ≥66). Much clearer.

Response: We appreciate the reviewer's important observation. We have revised the statement in question based on the reviewer’s suggestion.

Comment: Original significantly higher median W-DEQ scores

Good. However, you should specify the statistical test.

Example: ...reported significantly higher median W-DEQ scores than parous women (p = 0.004).

Response: We appreciate the reviewer's important observation. We have revised the statement in question based on the reviewer’s suggestion.

Comment: Table 2 Problems. The percentages are incomplete. Example: (28.3), (45.4) The frequencies are missing. Should be: 28 (28.3%) etc. Reviewers will definitely notice this.

Response: We thank the reviewer for this observation. We clarify that Table 2 presents categorical variables as n (%), where the frequencies are provided first followed by the corresponding percentages in brackets. We note that this format has been consistently applied throughout the table (e.g., “28 (28.3)”), with the header indicating “n (%)” to guide interpretation. To improve clarity, we have reviewed the table formatting to ensure that both frequencies and percentages are presented clearly and consistently aligned across all variables.

Comment: Regression Results Section. IMPORTANT STATISTICAL REPORTING ISSUE. Current β: -1.25, 95%CI: -1.74, 0.77. This is incorrect because: CI needs “to” or parentheses format. Correct format: β = -1.25, 95% CI: -1.74 to -0.77

OR β = -1.25 (95% CI: -1.74, -0.77). Currently the upper limit appears positive because of punctuation confusion. This issue appears multiple times.

Response: We are grateful to the reviewer for these observations. We have revised these sections of the manuscript based on the reviewer's suggestions and addressed all the issues and ensured that they are consistent throughout the work. Thank you.

Comment: Another Important Error. Current: planning current pregnancy (β: 25.71...) But later: not planning the current pregnancy (adj. β: 8.84...). These contradict each other. Likely correction: unadjusted model should refer to: not planning the current pregnancy. This inconsistency must be corrected. Family Support Result. Current having moderate (adj. β: -13.10, 95%CI: 18.70, -7.50): Incorrect CI order. Should be: adjusted β = -13.10, 95% CI: -18.70 to -7.50. Another Grammar Issue

Response: We are grateful to the reviewer for these observations. We have revised these sections of the manuscript based on the reviewer's suggestions.

Comment: Current: family’s support Use: family support throughout manuscript. Avoid apostrophe.

Response: We are grateful to the reviewer for this observation, and in line with it, we have revised it to "family support". Thank you.

Comment: Table 3 Review. Very strong overall. Small consistency fixes. Use one reporting style consistently: Either Adj. β OR adjusted β Choose one style. Current: 1:00 = reference category. Correct: 1.00 = reference category

Response: We are grateful to the reviewer for the comment, and in line with that, we have used ‘adjusted β’ consistently throughout the manuscript

Comment: Table 4 Review. Strong scientifically. Grammar issue. Current: family support was significantly associated. Plural subject problem. Correct: family support were significantly associated OR better: moderate and strong family support were significantly associated with FoC.

Response: We are grateful to the reviewer for these observations. We have revised these sections of the manuscript based on the reviewer's suggestions. It now reads as; moderate and strong family support were significantly associated with FoC.

Comment: Discussion - This section is strong and thoughtful. Opening Paragraph. Excellent summary. Small improvement. Current: underpin a critical need. Suggested: highlight a critical need. More natural wording.

Response: We are grateful to the reviewer for these observations. We have revised these sections of the manuscript based on the reviewer's suggestions.

Comment: Paragraph Comparing Prevalence. Current: median WDEQ score Should be: median W-DEQ score

Response: We appreciate the reviewer for this observation. WDEQ has been written as W-DEQ, but because the spelling extends to the next line, it is appearing as WDEQ. Thank you

Comment: Consistency issue. Important Statistical Issue. Current: median W-DEQ score in our study was 41, comparable to Malawi (mean: 47) Median and mean are not directly comparable.

Suggested: ...although direct comparison should be interpreted cautiously because the Malawi study reported mean rather than median scores. Very important for reviewers/statisticians.

Response: We appreciate the reviewer's important observation. We have added a caution in the revised manuscript regarding that comparison and note that it should be interpreted with caution, given that the Malawi study reported mean rather than median scores.

Comment: Nulliparous Explanation Paragraph Very good.. IPV Paragraph Excellent interpretation. Family Support Paragraph: Scientifically strong but repetitive. You mention: importance of family support, social networks, supportive environments several times. You can shorten by ~25% without losing meaning.

Response: We are grateful to the reviewers for these insightful comments. We have reduced the discussion on the family support paragraph and removed all repetitions without losing meaning.

Comment: IMPLICATIONS SECTION. Strong practical implications. Grammar Issue

Current: they should also integrate patient-centred strategies. Problem: Who is “they”? Suggested: routine ANC programs should also integrate patient-centred strategies

Response: We are grateful to the reviewer for this comment. We have revised the statement in question, and it now reads as; Our findings suggest that, in addition to screening for FoC, routine ANC programs should also integrate patient-centred strategies that account for the unique risk profiles within each parity group.

Comment: LIMITATIONS SECTION. Very good and transparent.

Response: Thank you for your comment

Comment: Your conclusion is scientifically sound and appropriately balanced. Main Strength: You avoided overstating causality, which is excellent for a cross-sectional study. IMPORTANT ISSUES TO CORRECT. Typographical Error. Current: parityspecific risk profiles. Correct: parity-specific risk profiles

Response: We appreciate the reviewer's comments. ‘Parity-specific’ was written as such, but it extends to the next line, and that is why it appears that there was no ‘–‘

Comment: Repetitive Wording. Current: These findings underscore the need for parity-specific strategies in addressing FoC during ANC. Suggested: These findings underscore the need for parity-tailored strategies to address FoC during ANC. More concise and smoother.

Response: We are grateful to the reviewer for this observation. We have revised the section of the manuscript based on the reviewer's suggestions and removed the repetition.

Comment: Slight Overstatement. Current: could collectively reduce childbirth-related fear and promote better maternal health outcomes. Since your study is observational, avoid implying intervention effectiveness too strongly. Better: may help reduce childbirth-related fear and improve maternal well-being

Response: We are grateful to the reviewer for this observation. We have revised the section of the manuscript based on the reviewer's suggestions and removed the repetition

Comment: ACKNOWLEDGEMENT REVIEW. Major Typographical Error. Current: employeesmanagement and staff. This appears to be a copy-paste duplication error. Correct Version: We would like to thank the management and staff of HopeXchange Medical Centre, the study participants, and the research assistants for their valuable contributions to this study.

Response: We are grateful to the reviewer for this observation. We have revised the section of the manuscript based on the reviewer's suggestions and removed the repetition

Comment: REFERENCES REVIEW. Your references are strong overall, current, and relevant. However, several important formatting and consistency issues need correction. MAJOR REFERENCE ISSUES. Duplicate Reference. References 11 and 28 are duplicates. Both are:

Khwepeya M, Lee GT... You must remove one duplicate reference and renumber accordingly. Typographical Error in Reference 22. Current: Wjma K. Correct: Wijma K.. Very important because this is the main W-DEQ reference. 3. Broken Reference. Reference 48. Current: 261&partnerID=40&md5=32f6a261400d8df1c8fb50cd1fad5f96. This is clearly accidental database export text and must be deleted. Broken Supporting Information Section

Current: Datasets used f Incomplete sentence. Correct or remove. Example: Supporting information. The datasets used and/or analysed during the current study are available from the corresponding author upon reasonable request. (Only if true.). Inconsistent Journal Formatting. Examples: “BMC Womens Women's Health” “Front Public Heal.” “Women and Birth.” “J Obstet Gynaecol (Lahore).” Journal abbreviations should follow one indexing style consistently (usually PubMed/NLM). Inconsistent Use of Page Ranges. Examples: “763– 7”, “121–6” Need consistent formatting. Example: 763–767, 121–126 depending on journal style. Capitalization Inconsistency. Example: The Influence of Fear During Pregnancy

Some references use sentence case, others title case. Most journals require sentence case. Example: Correct: The influence of fear during pregnancy, labour and delivery on birth outcomes among post-delivery women. W-DEQ Formatting Always use: W-DEQ. Not: WDEQ

Response: We are very grateful to the reviewer for these important observations. We have addressed all the issues that were raised by the reviewer, which include removing duplicates, ensuring consistency, capitalisation consistency, etc.

Regarding the supplementary requirement, it is a journal requirement. According to PLOS ONE, authors are expected to share the data used for the analysis either in a repository or by adding it to the files for submission.

Reviewer 4

Comment: I would like to thank the authors for their response and the effort they have put in to revise the manuscript and also they have addressed all the concerns raised in the review.

Response: We are grateful to you for your time and for providing us with comments we believe have strongly enhanced the rigour of our work.

Attachment Submitted filename: AUTHORS RESPONSES.pdf

# Peer review: Decision Letter 4

**DOI:** 10.1371/journal.pone.0358457.r009

**Authors:** Mesfin Abebe

5 Aug 2026

Dear Dr. Opoku,

Thank you for submitting your manuscript to PLOS ONE. After careful consideration, we feel that it has merit but does not fully meet PLOS ONE’s publication criteria as it currently stands. Therefore, we invite you to submit a revised version of the manuscript that addresses the points raised during the review process.

==============================

**ACADEMIC EDITOR:** Please note the following when preparing your revision:

- Clearly address all reviewer comments.
- Distinguish between changes required for acceptance and those recommended for improvement.
- Where reviewer feedback conflicts, follow the guidance provided here.
- Ensure that your revisions are justified based on PLOS ONE’s publication criteria, rather than novelty or perceived impact.

==============================

Please submit your revised manuscript by Sep 19 2026 11:59PM. If you will need more time than this to complete your revisions, please reply to this message or contact the journal office at plosone@plos.org. When you're ready to submit your revision, log on to https://www.editorialmanager.com/pone/ and select the 'Submissions Needing Revision' folder to locate your manuscript file.

- A letter that responds to each point raised by the academic editor and reviewer(s). You should upload this letter as a separate file labeled 'Response to Reviewers'.
- A marked-up copy of your manuscript that highlights changes made to the original version. You should upload this as a separate file labeled 'Revised Manuscript with Track Changes'.
- An unmarked version of your revised paper without tracked changes. You should upload this as a separate file labeled 'Manuscript'.

If you would like to make changes to your financial disclosure, please include your updated statement in your cover letter. Guidelines for resubmitting your figure files are available below the reviewer comments at the end of this letter.

If applicable, we recommend that you deposit your laboratory protocols in protocols.io to enhance the reproducibility of your results. Protocols.io assigns your protocol its own identifier (DOI) so that it can be cited independently in the future. For instructions see: https://journals.plos.org/plosone/s/submission-guidelines#loc-laboratory-protocols. Additionally, PLOS ONE offers an option for publishing peer-reviewed Lab Protocol articles, which describe protocols hosted on protocols.io. Read more information on sharing protocols at https://plos.org/protocols?utm_medium=editorial-email&utm_source=authorletters&utm_campaign=protocols.

As the corresponding author, your ORCID iD is verified in the submission system and will appear in the published article. PLOS supports the use of ORCID, and we encourage all coauthors to register for an ORCID iD and use it as well. Please encourage your coauthors to verify their ORCID iD within the submission system before final acceptance, as unverified ORCID iDs will not appear in the published article. *Only*  the individual author can complete the verification step; PLOS staff *cannot*  verify ORCID iDs on behalf of authors.

We look forward to receiving your revised manuscript.

Kind regards,

Mesfin Abebe

Academic Editor

PLOS One

Journal Requirements:

If the reviewer comments include a recommendation to cite specific previously published works, please review and evaluate these publications to determine whether they are relevant and should be cited. There is no requirement to cite these works unless the editor has indicated otherwise.

Please review your reference list to ensure that it is complete and correct. If you have cited papers that have been retracted, please include the rationale for doing so in the manuscript text, or remove these references and replace them with relevant current references. Any changes to the reference list should be mentioned in the rebuttal letter that accompanies your revised manuscript. If you need to cite a retracted article, indicate the article’s retracted status in the References list and also include a citation and full reference for the retraction notice.

[Note: HTML markup is below. Please do not edit.]

Reviewers' comments:

Reviewer's Responses to Questions

**Comments to the Author**

Reviewer #4: All comments have been addressed

Reviewer #5: (No Response)

**********

2. Is the manuscript technically sound, and do the data support the conclusions?

Reviewer #4: Yes

Reviewer #5: Partly

**********

3. Has the statistical analysis been performed appropriately and rigorously?

Reviewer #4: Yes

Reviewer #5: Yes

**********

4. Have the authors made all data underlying the findings in their manuscript fully available?

The PLOS Data policy

Reviewer #4: Yes

Reviewer #5: Yes

**********

5. Is the manuscript presented in an intelligible fashion and written in standard English?

Reviewer #4: Yes

Reviewer #5: Yes

**********

Reviewer #4: I have evaluated the clean copy of the revised manuscript and feel that the author have addressed all the issues/concerns raised by the reviewer/ reviewers. I would like to thank the authors for their constructive response and the effort they have put in to revise the manuscript. I feel that the manuscript meets the standards of the journal and hence, recommend it for acceptance.There are a few minor font size inconsistencies in line 69,133,134 and 176 (clean copy) which can be resolved in the final proof reading.

Reviewer #5: Dear Authors

The article provides evidence on associated factors of FoC in a resources-limited setting. The findings may help to improve health care to pregnant women and contribute to reduce maternal mortality.

I made some comments that need to be addressed to improve the quality of the manuscript. My main concern is the identification of family support as a risk factor for FoC.

Best regards

**********

what does this mean?). If published, this will include your full peer review and any attached files.

If you choose “no”, your identity will remain anonymous but your review may still be made public.

**Do you want your identity to be public for this peer review?**  For information about this choice, including consent withdrawal, please see our Privacy Policy

Reviewer #4: No

Reviewer #5: **Yes:** Dr KEUGOUNG Basile

**********

[NOTE: If reviewer comments were submitted as an attachment file, they will be attached to this email and accessible via the submission site. Please log into your account, locate the manuscript record, and check for the action link "View Attachments". If this link does not appear, there are no attachment files.]

To ensure your figures meet our technical requirements, please review our figure guidelines: https://journals.plos.org/plosone/s/figures

You may also use PLOS’s free figure tool, NAAS, to help you prepare publication quality figures: https://journals.plos.org/plosone/s/figures#loc-tools-for-figure-preparation.

NAAS will assess whether your figures meet our technical requirements by comparing each figure against our figure specifications.

Attachment Submitted filename: Reviewer comments.docx

# Author comment: Author response to Decision Letter 5

**DOI:** 10.1371/journal.pone.0358457.r010

9 Aug 2026

Reviewer 4

Comment: I have evaluated the clean copy of the revised manuscript and feel that the author have addressed all the issues/concerns raised by the reviewer/ reviewers. I would like to thank the authors for their constructive response and the effort they have put in to revise the manuscript. I feel that the manuscript meets the standards of the journal and hence recommend it for acceptance.

Response: We appreciate the reviewer for these remarks. We believe that the reviewers’ comments have helped improve the quality of the work.

Comment: There are a few minor font size inconsistencies in line 69,133,134 and 176 (clean copy) which can be resolved in the final proof reading.

Response: We are grateful to the reviewer for drawing our attention to this. We have corrected the inconsistencies in the font size in lines 69, 133, 134, and 176 as suggested.

Reviewer 5

Comment: The article provides evidence on associated factors of FoC in a resources-limited setting. The findings may help to improve health care to pregnant women and contribute to reduce maternal mortality. I made some comments that need to be addressed to improve the quality of the manuscript. My main concern is the identification of family support as a risk factor for FoC.

Response: We appreciate the reviewer for these remarks. Particularly, we are also grateful to all reviewers for making time to review this work, as we believe their comments have improved the quality of the manuscript.

We have addressed all comments raised, including the identification of family support as a risk factor for FoC.

Comment: Line 68-69- emotional symptoms such as mood swings and somatisation, as well as cognitive effects, 69 including impaired decision-making (2–4).

Suggestion - emotional symptoms (e.g., mood swings, somatisation) and cognitive effects including impaired decision-making (2–4).

Response: We are grateful to the reviewer for this suggestion. The sentence in reference has been revised to read as;

It may manifest through emotional symptoms (e.g., mood swings, somatisation) and cognitive effects, including impaired decision-making (2–4).

Comment: Line 117 - Complete the description of HopeXchange Medical Centre. 600 deliveries represent 12% of the population and around 50 per month.

How many women were received during ANC1 in 2024.? How many cases for curative consultation? This Private not-for-profit surely attracts women outside the estimated population. Are there some reasons why this health facility was selected?

Response: We thank the reviewer for this important comment. We have provided additional useful context for understanding the study setting. However, the facility records available to the research team did not provide a reliable figure for the number of women who registered for ANC1 or the number of curative consultations received in 2024. We have therefore not provided estimates for these indicators to avoid introducing potentially inaccurate information. The statement that portrays 5000 ANC utilisation in 2024 has also been revised as it only refers to estimates for deliveries in 2024

Comment: Line 131 … a 10% non-response rate, the final required sample size was 290 pregnant women.

Replace with : …a 10% non-response rate, the final required sample size was 290 pregnant women (Figure 1).

Response: e thank the reviewer for this helpful suggestion. Although the estimated sample size after allowing for a 10% non-response rate was 290 pregnant women, 282 participants were ultimately recruited and participated in the study. The final number of participants was reported separately in line 222 and is reflected in Figure 1. We therefore believe that adding the citation to Figure 1 to the sample size estimation statement may be misleading, as Figure 1 presents the participant flow based on the 282 women who participated in the study rather than the initially estimated sample size of 290. We have consequently retained the statement in its current form.

Comment: Ethical approval. Do some researchers work in the HopeXchange Medical Centre facility as health care provider? If yes, the authors should indicate

Response: We are grateful to the reviewer for this comment. Yes, one of the authors works at the study site, and this has been reported in the revised manuscript. It reads as;

One of the researchers (JOA) is employed at the study site. However, the researcher’s affiliation with the facility did not influence the conduct, analysis, interpretation or outcome of the study.

Comment: Page 10, Figure Prevalence of FoC among study participants

- Indicate the number of the Figure (e.g Figure 1)

- Cite in text

As there are only two items, Remove the legend and insert directly Cases and Non-cases directly in the Figure

Response: We thank the reviewer for this helpful comment. In accordance with the recommendation, the figure has been numbered as Figure 1 and is cited appropriately in the text. We also attempted to incorporate the labels “Cases” and “Non-cases” directly into the figure and remove the legend. However, the direct labelling reduced the clarity and readability of the figure. We have therefore retained the legend to ensure clear interpretation of the figure.

Comment: age 29 - Family support was inversely associated with FoC, consistent with evidence highlighting the protective role of social support against maternal anxiety and childbirth-related stress (50,51). Women with strong support systems tend to feel more empowered, informed, and emotionally secure, which may reduce fears and anxiety (52). This finding underscores the importance of psychosocial supports in shaping childbirth experiences and suggests that integrating family oriented approaches into ANC services could help reduce FoC and improve maternal…

Comment - This means that by providing family support, women will be more empowered, informed, and emotionally secure, which may reduce fears and anxiety.

This is Iin contradiction with the results showed that women with family support have more FoC.Maybe, there is a bias that family support is mostly provided to women who have already FoC. Therefore, family support will not be considered as an associated factor. Could the authors explain and discuss more this factor?

Response: We appreciate the reviewer for this comment. Looking at the results again, with poor family support used as the reference category, compared with women reporting poor family support, those reporting moderate family support had lower FoC scores (β = −23.10, 95% CI: −31.91 to −14.30, p < 0.001), while those reporting strong family support also had lower FoC scores (β = −27.94, 95% CI: −38.77 to −17.11, p < 0.001). Thus, our findings indicate an inverse association between family support and FoC, rather than higher FoC among women receiving family support. However, we also agree with the reviewer regarding the potential directionality of this relationship. Importantly, given the cross-sectional design of this study, we can establish that the exposure preceded the outcome. The discussion has been revised based on these. We have subsequently acknowledged them in the revised discussion.

Comment: Page 17 - Line 10369 : generalizability. This is US and the authors used the British English. Therefore, the spelling should be generalisability

Response: We are grateful to the reviewer for this comment. We have corrected the spelling as suggested.

Attachment Submitted filename: AUTHORS_RESPONSES_auresp_5.pdf

# Peer review: Decision Letter 5

**DOI:** 10.1371/journal.pone.0358457.r011

**Authors:** Mesfin Abebe

1 Sep 2026

<p>Fear of childbirth among antenatal care attendees in a primary care setting in Kumasi, Ghana: Prevalence and parity-stratified determinants

PONE-D-25-51554R5

Dear Dr. Opoku,

We’re pleased to inform you that your manuscript has been judged scientifically suitable for publication and will be formally accepted for publication once it meets all outstanding technical requirements.

Within one week, you’ll receive an e-mail detailing the required amendments. When these have been addressed, you’ll receive a formal acceptance letter and your manuscript will be scheduled for publication.

An invoice will be generated when your article is formally accepted. Please note, if your institution has a publishing partnership with PLOS and your article meets the relevant criteria, all or part of your publication costs will be covered. Please make sure your user information is up-to-date by logging into Editorial Manager at Editorial Manager® and clicking the ‘Update My Information' link at the top of the page. For questions related to billing, please contact billing support.

If your institution or institutions have a press office, please notify them about your upcoming paper to help maximize its impact. If they’ll be preparing press materials, please inform our press team as soon as possible -- no later than 48 hours after receiving the formal acceptance. Your manuscript will remain under strict press embargo until 2 pm Eastern Time on the date of publication. For more information, please contact onepress@plos.org.

Kind regards,

Mesfin Abebe

Academic Editor

PLOS One

Additional Editor Comments (optional):

Reviewers' comments:

Reviewer's Responses to Questions

**Comments to the Author**

Reviewer #4: All comments have been addressed

Reviewer #5: All comments have been addressed

**********

2. Is the manuscript technically sound, and do the data support the conclusions?

Reviewer #4: Yes

Reviewer #5: Yes

**********

3. Has the statistical analysis been performed appropriately and rigorously?

Reviewer #4: Yes

Reviewer #5: Yes

**********

4. Have the authors made all data underlying the findings in their manuscript fully available?

The PLOS Data policy

Reviewer #4: Yes

Reviewer #5: Yes

**********

5. Is the manuscript presented in an intelligible fashion and written in standard English?

Reviewer #4: Yes

Reviewer #5: Yes

**********

Reviewer #4: The authors have addressed all the concerns raised by the reviewers' and I feel that the manuscript meets the standard of the journal and hence recommend it for acceptance.

Reviewer #5: Dear Authors

Thank you for taking into consideration my comments and for providing a clear response.

This has improved the quality of the Manuscript.

Best regards

**********

what does this mean?). If published, this will include your full peer review and any attached files.

If you choose “no”, your identity will remain anonymous but your review may still be made public.

**Do you want your identity to be public for this peer review?**  For information about this choice, including consent withdrawal, please see our Privacy Policy

Reviewer #4: No

Reviewer #5: **Yes:** Basile KEUGOUBG

**********

# Editor report: Acceptance letter

**DOI:** 10.1371/journal.pone.0358457.r012

**Authors:** Mesfin Abebe

PONE-D-25-51554R5

PLOS One

Dear Dr. Opoku,

I'm pleased to inform you that your manuscript has been deemed suitable for publication in PLOS One. Congratulations! Your manuscript is now being handed over to our production team.

At this stage, our production department will prepare your paper for publication. This includes ensuring the following:

* All references, tables, and figures are properly cited

* All relevant supporting information is included in the manuscript submission,

* There are no issues that prevent the paper from being properly typeset

You will receive further instructions from the production team, including instructions on how to review your proof when it is ready. Please keep in mind that we are working through a large volume of accepted articles, so please give us a few days to review your paper and let you know the next and final steps.

Lastly, if your institution or institutions have a press office, please let them know about your upcoming paper now to help maximize its impact. If they'll be preparing press materials, please inform our press team within the next 48 hours. Your manuscript will remain under strict press embargo until 2 pm Eastern Time on the date of publication. For more information, please contact onepress@plos.org.

You will receive an invoice from PLOS for your publication fee after your manuscript has reached the completed accept phase. If you receive an email requesting payment before acceptance or for any other service, this may be a phishing scheme. Learn how to identify phishing emails and protect your accounts at https://explore.plos.org/phishing.

If we can help with anything else, please email us at customercare@plos.org.

Thank you for submitting your work to PLOS One and supporting open access.

Kind regards,

PLOS One Editorial Office Staff

on behalf of

Dr. Mesfin Abebe

Academic Editor

PLOS One
